# Cytotoxic and Antiviral Triterpenoids from the Mangrove Plant *Sonneratia paracaseolaris*

**DOI:** 10.3390/molecules22081319

**Published:** 2017-08-09

**Authors:** Kai-Kai Gong, Ping-Lin Li, Dan Qiao, Xing-Wang Zhang, Mei-Jun Chu, Guo-Fei Qin, Xu-Li Tang, Guo-Qiang Li

**Affiliations:** 1 Key Laboratory of Marine Drugs, Chinese Ministry of Education, School of Medicine and Pharmacy, Ocean University of China, Qingdao 266003, China; gongkaikai1005@163.com (K.-K.G.); lipinglin@ouc.edu.cn (P.-L.L.); hdqiaodan@126.com (D.Q.); zhangxw@qibebt.ac.cn (X.-W.Z.); chumjun@163.com (M.-J.C.); qguofei1314@163.com (G.-F.Q.); 2 College of Chemistry and Chemical Engineering, Ocean University of China, Songling Road 238, Qingdao 266100, China; 3 Cancer Research institute, Binzhou Medical University Hospital, Yellow river second Road 661, Binzhou 256603, China

**Keywords:** mangrove plant, *Sonneratia paracaseolaris*, triterpenoids, cytotoxicities, anti-H1N1 activities

## Abstract

A chemical investigation was conducted on the aerial parts of the mangrove plant *Sonneratia paracaseolaris*, yielding five new triterpenoid paracaseolins A–E (**1**–**4**, and **11**) together with twelve known analogues (**5**–**10**, **12**–**17**). Their structures were established by extensive spectroscopic methods and comparisons their spectroscopic data with those of the known related compounds. The cytotoxicities against P388, HeLa, A549, and K562 tumor cell lines and anti-H1N1 (Influenza A virus) activities for the isolates were evaluated. Compound **4** showed potent cytotoxicity against the A549 cell line with an IC_50_ value of 1.89 µM, and compound **1** exhibited significant anti-H1N1 virus activity with an IC_50_ value of 28.4 µg/mL. A preliminary structure activity relationship was discussed.

## 1. Introduction

Mangrove plants of the genus *Sonneratia* (family Sonneratiaceae) consist of nine species and are generally distributed in the tropical and subtropical regions in the world [1]. The fruit, bark, and leaves of several *Sonneratia* plants have been used as folk medicines for treatment of various diseases, such as asthma, febris, ulcers, hepatitis, piles, sprains, and hemorrhages [2]. So far, almost half of the species of the genus have been studied for their chemical constituents, yielding various types of compounds, such as triterpenoids, steroids, alkaloids, flavonoids, aromatics, and tannins, some of which exhibited cytotoxic [3,4,5], anti-HIV [5], and antioxidant [6] activities. 

**Figure 1:**
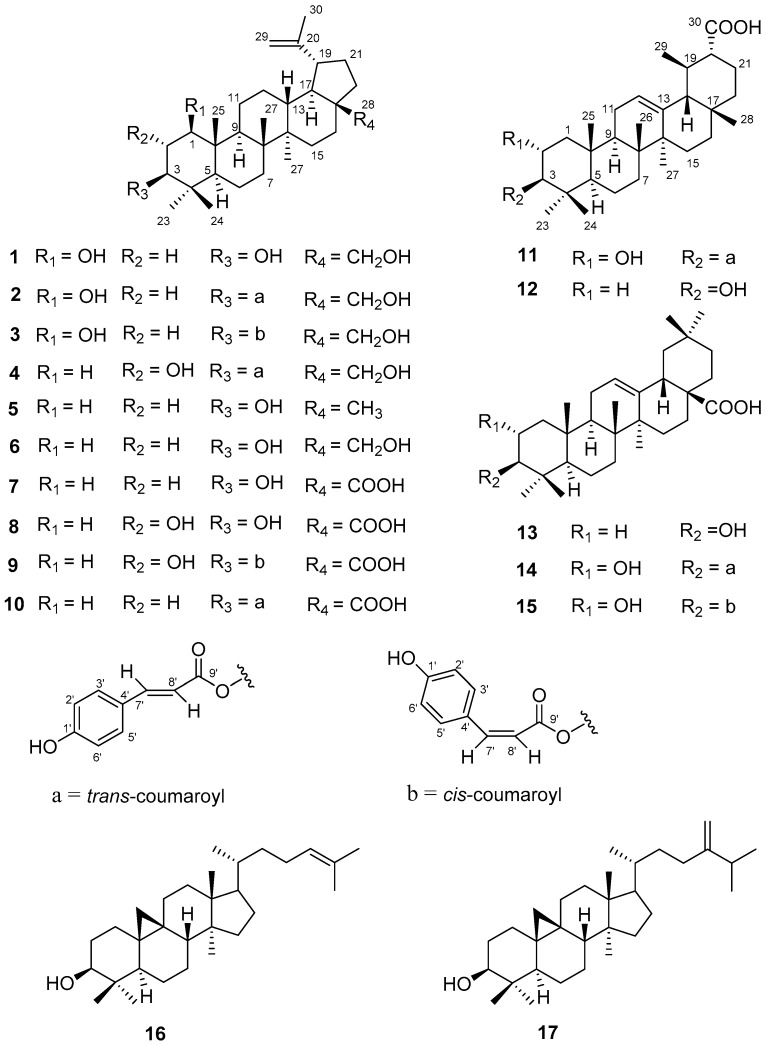
Structures of compounds **1**–**17**.

*Sonneratia paracaseolaris* is one of the six *Sonneratia* species in China [7] which was reported to be a natural hybrid of *Sonneratia alba* and *Sonneratia caseolaris* [8,9]. At present, only one report regarding the chemical study of *Sonneratia paracaseolaris* provided a novel α-alkylbutenolide dimer [10], triggering our interest to study the chemical profiles of *Sonneratia paracaseolaris*. In the previous chemical and bioassay experiments, we found that the methanol extract showed moderate cytotoxicities against the A549 cell line and was rich in triterpenoids. In order to find bioactive triterpenoids from this plant, combined chemistry and bioassay-guided quick isolation of triterpenoid-rich portions of the methanol extract yielded the five new triterpenoid paracaseolins A–E (**1**–**4** and **11**), together with twelve known ones (**5**–**10**, **12**–**17**) (Figure 1). These triterpenoid compounds could be divided into four carbon skeletons: lupane-, ursane-, oleanane-, and cycloartane-types. The major structure isolated from the species was characterized by lupane-type triterpenoids (**1**–**10**). All the isolates (**1**–**17**) were evaluated for cytotoxic activities against the selected P388, HeLa, A549, and K562 tumor cell lines, as well as anti-influenza A H1N1 virus activities. Herein, we describe the isolation, structural elucidation, and the cytotoxic and anti-H1N1 activities of all the isolates.

## 2. Results and Discussion

### 2.1. Structure Elucidation

Paracaseolin A (**1**) was isolated as irregular plates with a molecular formula C_30_H_50_O_3_ established by HR-ESI-MS (high resolution electrospray ionization mass spectrometry) ([M − H]^−^ at *m/z* = 457.3688; calcd. for 457.3676). The IR absorption bands at 3359, 1681, and 881 cm^−1^ were assigned to hydroxyl and olefinic groups. ^13^C-NMR (Table 1) and DEPT (distortionless enhancement by polarization transfer) spectra exhibited a total of 30 carbon signals, which were classified into six methyls, eleven methylenes (including one oxymethylene and one olefinic carbon), seven methines (including two oxymethines), and six quaternary carbons (including one olefinic carbon). The ^1^H-NMR spectrum (Table 1) showed five singlet methyls at δ_H_ 0.61 (3H, s, H-24), 0.76 (3H, s, H-25), 0.83 (3H, s, H-23), 0.93 (3H, s, H-27), and 0.98 (3H, s, H-26), indicating the presence of a triterpenoidal skeleton of compound **1**. Additionally, two terminal olefinic proton signals at δ_H_ 4.53 (1H, s, H-29) and 4.66 (1H, s, H-29), along with an olefinic methyl singlet at δ_H_ 1.63 (3H, s, H-30), indicated the presence of an isopropenyl residue possibly belonging to a lupane skeleton [11]. In fact, the NMR data of **1** were closely similar to the reported lup-20(29)-en-2α,3β,28-triol, except for those of C1–C3 in the A ring [12]. Connective ^1^H-^1^H COSY (hydrogen-hydrogen correlation spectroscopy) correlations from H-1 to H-3 and the HMBC (heteronuclear multiple bond correlation) cross peaks of H_3_-25 with C-1 (δ_C_ 77.9), C-5 (δ_C_ 52.6), and C-10 (δ_C_ 42.9), of H_3_-23 and H_3_-24 with C-3 (δ_C_ 74.0), C-4 (δ_C_ 38.0), and C-5 further suggesting compound **1** to be lup-20(29)-en-2,3,28-triol. The relative configurations of H-1 and H-3 were assigned as axial α-orientation by the half-peak-width data, 22.1 Hz for H-1 and 22.8 Hz for H-3 [13], together with the NOESY (nuclear overhauser effect spectroscopy) correlations between H-3 (δ_H_ 2.96, m) and H_3_-23 (δ_H_ 0.83, s) and between H-1 (δ_H_ 3.14, m) and H-5 (δ_H_ 0.46, d, *J* = 11.0 Hz), which was consistent with the stable cyclohexane conformation. Thus, compound **1** was determined as 1β,3β-dihydroxy betulin, named as paracaseolin A (**1**).

Paracaseolin B (**2**), isolated as fine needles, had the molecular formula C_39_H_56_O_5_ established by HR-ESI-MS data ([M − H]^−^ at *m*/*z* 603.4058; calcd. for 603.4044). The UV spectrum exhibited absorption maxima at 228 and 312 nm, suggesting the presence of aromatic rings in the molecule. The IR spectrum showed absorption bands for hydroxyl (3376 cm^−1^), α,β-unsaturated carbonyl (1682 cm^−1^), and aromatic (1604, 1513, 1451, and 831 cm^−1^) functionalities. In the ^1^H-NMR spectrum (Table 1) of **2**, the signals at δ_H_ 7.53 (2H, d, *J* = 8.6 Hz, H-2′, H-6′), 7.51 (1H, d, *J* = 16.0 Hz, H-7′), 6.77 (2H, d, *J* = 8.6 Hz, H-3′, H-5′), and 6.33 (1H, d, *J* = 16.0 Hz, H-8′) implied the presence of the partial structure, a *trans*-*p*-coumaroyl functionality. Additional 1D NMR data for **2** were almost identical to **1**, except for a distinct downfield shift of H-3 at δ_H_ 4.47 (1H, dd, *J* = 11.9, 4.7 Hz, H-3) in **2**, indicating the position of the *O*-*trans*-*p*-coumaroyl functionality at C-3. This speculation was further confirmed by HMBC correlations of H-3, H-7′ and H-8′ with C-9′ (δ_C_ 166.2). The large coupling constants of H-3 (*J* = 11.9 Hz) and H-1 (*J* = 10.8 Hz), as well as the NOESY correlations of both H-3 and H-1 with H-5 could assign their axial α-orientations. Compound **2** was then identified as 1β-hydroxy-3β-*O*-*trans*-*p*-coumaroyl betulin.

Paracaseolin C (**3**), a white amorphous powder, had the same molecular formula of C_39_H_56_O_5_ as compound **2** from the HR-ESI-MS data ([M − H]^−^ at *m*/*z* 603.4056; calcd. for 603.4044). A comparable study of 1D NMR data (Table 2) of **3** with **2** (Table 1) showed their closely structural similarity except for the small coupling constant of H-7′ and H-8′ (*J*_H-7′/H-8′_ = 13.0 Hz) in **3**, indicating the presence of a *cis*-*p*-coumaroyl group. Accordingly, compound **3** was determined as 1β-hydroxy-3β-*O*-*cis*-*p*-coumaroyl betulin.

Paracaseolin D (**4**), a white amorphous powder, had the molecular formula C_39_H_56_O_5_, as determined by HR-ESI-MS data ([M − H]^−^ at *m/z* 603.4056; calcd. for 603.4044). 1D NMR spectra of compound **4** were very similar to those of **2** apart from a slight difference in ring A. The split pattern of H-3 (δ_H_ 4.48, d, *J* = 10.0 Hz) in **4** showed double peak instead of multiple peak in **2** and the carbon signal at C-3 shifted from δ_C_ 76.7 in **2** to δ_C_ 83.4 in **4** (Table 2), suggesting the ring A of compound **4** to be 2, 3-dioxyged structure which was supported by ^1^H-^1^H COSY correlation between H-2 (δ_H_ 3.67) and H-3. The *O*-*trans*-*p*-coumaroyl group was also assigned at C-3 on the basis of the HMBC correlation of H-3 with C-9′ (δ_C_ 166.7). The half-peak-width value (26.6 Hz) of H-2 further indicated an axial β-orientation. Compound **4** was finally determined as 2α-hydroxy-3β-*O*-*trans*-*p*-coumaroyl betulin. 

**Figure 2:**
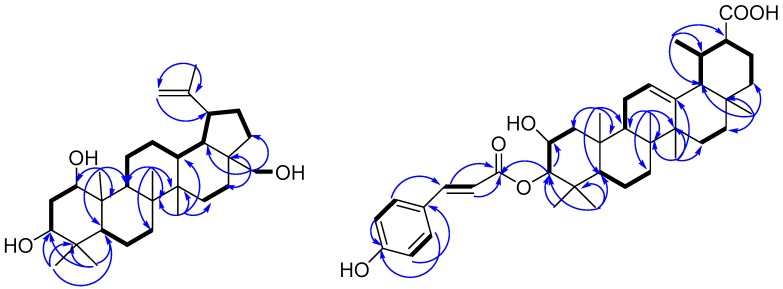
^1^H-^1^H COSY (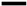), and selected HMBC (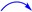) correlations of compounds **1** and **11**.

HR-ESI-MS data ([M − H]^−^ at *m*/*z* 617.3850; calcd. for 617.3837) showed the molecular formula of paracaseolin E (**11)** as C_39_H_54_O_6_. The IR spectrum displayed hydroxyl (3485 cm^−1^), carboxylic acid (1710 cm^−1^), α,β-unsaturated carbonyl (1682 cm^−1^) and phenyl (1603, 1510, 1457, and 832 cm^−1^) absorption bands. A *trans*-coumaroyl moiety was observed by comparison the 1D NMR data with those of **2** (Table 1). Apart from the *trans*-coumaroyl moiety, 30 skeleton carbon signals were observed in ^13^C-NMR spectrum. In its ^1^H-NMR spectrum, seven characteristic methyl signals including six singlet methyls (δ_H_ 0.76, 0.80, 0.85, 0.92, 0.97, 1.07) and one doublet (δ_H_ 0.83, d, *J* = 6.1 Hz) further suggested compound **11** could be a ursonic acid type triterpenoid similar to the known dulcioic acid, the co-isolated compound **12** (Figures S37–S40, Supporting Information) [14]. The two proton signals at δ_H_ 3.69 (1H, m, W_1/2)_ = 22.9 Hz, H-2) and 4.51 (1H, d, *J* = 9.9 Hz, H-3), as well as two carbon resonances at δc 64.8 (C-2) and 83.6 (C-3), and the additional *trans*-coumaroyl signals, indicated the ring A of **11** identical with **4**. This speculation was further confirmed by the combined analysis of ^1^H-^1^H COSY and HMBC spectra (Figure 2). The doublet methyl was assigned at C-19 rather than C-20 due to the ^1^H-^1^H COSY correlations of H-19 (δ_H_ 1.32, m) with H-18 (δ_H_ 2.12, d, *J* = 11.0 Hz) and H_3_-29 (δ_H_ 0.83, d, *J* = 6.1 Hz), and the HMBC correlations of H_3_-29 with C-18 (δc 52.4) and C-20 (δc 38.5). The substituted pattern of 2-hydroxy-3-*O*-*trans*-*p*-coumaroyl was also supported by the HMBC correlation of H-3 and C-9′ (δc 166.7), and the ^1^H-^1^H COSY correlation between H-3 and H-2. The relative configuration of 2α-hydroxy-3β-*O*-*trans*-*p*-coumaroyl was in agreement with compound **4** dependent on the half-peak-width value (26.9 Hz) for H-2 and the NOESY correlation of H-2 with H_3_-25, together with the large coupling constant of H-3 (*J*_H-3/H-2_ = 9.9 Hz). Accordingly, compound **11** was identified as 2α-hydroxy-3β-*O*-*trans*-*p*-coumaroyl dulcioic acid.

In addition to the new compounds, a spectroscopic data comparison with the literature allowed the known compounds to be identified as lupeol (**5**) [15,16], betulin (**6**) [11,17], betulinic acid (**7**) [11,18], alphitolic acid (**8**) [19], 3β-*O-cis*-*p*-coumaroyl alphitolic acid (**9**) [20,21], 3β-*O*-*trans*-*p*-coumaroyl betulinic acid (**10**) [22], dulcioic acid (**12**) [14], oleanolic acid (**13**) [23], 3β-*O-trans-p*-coumaroyl maslinic acid (**14**) [24], 3β-*O-cis-p*-coumaroyl maslinic acid (**15**) [25], cycloartenol (**16**) [26], and 24-methylenecycloartenol (**17**) [26], respectively.

### 2.2. Biological Evaluations

The cytotoxicities of the isolates were evaluated against the selected K562, P388, HeLa, and A549 tumor cell lines in vitro with adriamycin (ADM) as a positive control, and the results are summarized in Table 3. Almost all of the compounds were active against the P388 cell line and exhibited different levels of cytotoxicities against HeLa and A549 cell lines, but were not sensitive toward the K562 cell line. In particular, new compound **4** showed the strongest cytotoxicity against A549 cells with an IC_50_ value of 1.89 µM, and compounds **3** and **14** displayed low IC_50_ values, ranging from 11.04 to 13.10 µM, against A549, HeLa, and P388 cell lines, whereas only **15** showed cytotoxic activity against the K562 cell line with an IC_50_ value of 16.28 µM. Furthermore, a preliminary structure activity relationships were analyzed. Comparing the cytotoxic data of **1** and **3**, **8** and **9**, as well as **13** and **14** against HeLa and P388 cell lines, it is suggested that the introduction of an O-p-coumaroyl group at C-3 could increase the cytotoxicities no matter the *cis* or *trans* configuration, which is consistent with previous reports [21]. Another comparison between **2** and **4**, and **7** and **8** implied that 2-OH played significant roles on the cytotoxicities, whereas the OH group at C-1 and the substituent patterns at C-28 showed no effects, which could be predicted from a comparison between compounds **1** and **5**–**7**.

Antiviral activities of all the isolates against influenza A H1N1 virus (IAV) were also evaluated by CPE (the cytopathic effects) assay [27]. Only new compound **1** exhibited significant anti-H1N1 virus activity with an IC_50_ value of 28.4 µg/mL, very close to the positive control of Ribavirin with an IC_50_ value of 24.6 µg/mL. The other compounds showed no activity with inhibition rates less than 50% at 50 µg/mL (Table S2, Supporting Information).

## 3. Materials and Methods

### 3.1. General Experimental Procedures

Optical rotations were measured with a JASCO P-1020 polarimeter (JASCO, Tokyo, Japan). UV spectra were taken on a Beckman DU640 spectrophotometer (Beckman Coulter Inc., Brea, CA, USA). IR spectra were recorded on a NICOLET NEXUS 470 spectrophotometer (International Equipment Trading Ltd., Vernon Hills, IL, USA) in KBr discs, with υ in cm^−1^. ^1^H-, ^13^C-NMR, DEPT, and 2D NMR spectra were recorded on a JEOL JNM-ECP 600 (JEOL Ltd., Tokoyo, Japan) and Varian 500 spectrophotometers (Varian, Palo Alto, CA, USA), with δ in ppm with solvent residual signals as internal standards (DMSO: δ_H_ 2.50 ppm, δ_C_ 39.5 ppm), and *J* in Hz. Semi-preparative HPLC was performed using an ODS column (YMC-Pack ODS-A, 10 × 250 mm, 5 µm) (YMC Co. Ltd., Kyoto, Japan).; The ratios of solvent were described as a mixture by *v/v*. HR-ESI-MS was measured on a Thermo Scientific LTQ Orbitrap XL mass spectrometer (Thermo Fisher Scientific Inc., Waltham, MA, USA) in *m*/*z*. A Sephadex LH-20 (GE Healthcare, Uppsala, Sweden) and silica gel (SiO_2_; 100–200 mesh, 200–300 mesh, and 300–400 mesh; Qingdao Marine Chemical Inc., Qingdao, China) were used for column chromatography (CC), and TLC was carried out on silica gel GF_254_ (10–40 mm; Qingdao Marine Chemical Inc., Qingdao, China) plates; spots were visualized under UV light and by spraying with 5% H_2_SO_4_ in C_2_H_5_OH (*v/v*) followed by heating. 

### 3.2. Plant Material

The aerial parts of *Sonneratia paracaseolaris* were collected in Wenchang, Hainan Province, China, in October 2007, and was identified by Associate Prof. Cairong Zhong (Dongzhai Mangrove Forest National Nature Reserve). A voucher specimen (NO. WC-2007-10) was deposited at the State Key Laboratory of Marine Drugs, Ocean University of China, Qingdao, China.

### 3.3. Extraction and Isolation

The dried and powered aerial parts of *Sonneratia paracaseolaris* (11.5 kg) were extracted with methanol (30 L × 4 times, each, three days) at room temperature. The solvent was concentrated under reduced pressure to yield crude extract (490 g). After desalting with anhydrous methanol, the left residue (246 g) was subjected to vacuum liquid chromatography (VLC) on a silica gel column (100–200 mesh, 10 cm × 20 cm, 500 g) and eluted with a gradient of petroleum ether/acetone (*v/v* 100:1, 50:1, 25:1, 10:1, 5:1, 2:1, 1:1, 0:1, each 10 L) to yield ten fractions (Fr.1–Fr.10). Each fraction was detected by HPLC and was preliminarily bioassayed for cytotoxicities, and Fr. 4, 7–9 were found to be the most active fractions containing the main triterpenoids of this species. Thus, Fr.4 (1.017 g) was separated by silica gel CC (300–400 mesh, 2 cm × 20 cm, 20 g) eluted with petroleum ether/atone (*v/v* 25:1, 4 L) to afford three sub-fractions (Fr.4.1–Fr.4.3). Fr.4.2 (570.4 mg) was purified by reversed-phase silica gel CC (2 cm × 9 cm, 10 g) with a gradient of MeOH/H_2_O (*v/v* 60:40, 65:35, 70:30, 75:25, 80:20, 85:15, 90:10, 95:5, 100:0, each 200 mL) to yield Fr.4.2.1–Fr.4.2.8. Fr.4.2.8 (203 mg) was further separated by semi-preparative RP-HPLC (C18, MeOH, 100%, 1.5 mL/min) to obtain compounds **5** (15.3 mg, *t*_R_ 46.8 min), **16** (4.4 mg, *t*_R_ 75.6 min) and **17** (6.4 mg, *t*_R_ 80.0 min). Fr.7 (1.637 g) was subjected to silica gel CC (200–300 mesh, 3 cm × 20 cm, 50 g) using petroleum ether/acetone (*v/v* 20:1, 15:1, 10:1, 5:1, 2:1, each 1 L) as an eluent to yield four fractions (Fr.7.1–Fr.7.4). Fr.7.4 (1.264 g) was further chromatographed on silica gel CC (200–300 mesh, 2 cm × 20 cm, 30 g) eluted with CH_2_Cl_2_/EtOAc (*v/v* 50:1, 4 L) to afford compound **6** (200.3 mg, between 3 L and 4 L). Fr.8 (1.525 g) was chromatographed by a Sephadex LH-20 (3 cm × 75 cm, 500 g) eluted with MeOH to afford six sub-fractions (Fr.8.1–Fr.8.6). Fr.8.2 (125.3 mg) was further purified by semi-preparative RP-HPLC (C18, MeOH/H_2_O, 85:15, 1.5 mL/min) to yield compounds **7** (10.8 mg, *t*_R_ 40.0 min), **12** (3.4 mg, *t*_R_ 47.1 min,) and **13** (10.8 mg, *t*_R_ 45.1 min). Fr.9 (6.398 g) was submitted to Sephadex LH-20 column chromatography (3 cm × 75 cm, 500 g) with MeOH to remove the pigments and carbohydrates, and further purified by silica gel column (200–300 mesh, 4 cm × 20 cm, 130 g) eluted with petroleum ether/acetone (*v/v* 20:1, 10:1, 5:1, 2:1, each 2 L) to afford nine sub-fractions (Fr.9.1–Fr.9.9). Fr.9.1 (1.041 g) was further separated to six fractions (Fr.9.1.1–Fr.9.1.6) by silica gel CC (300–400 mesh, 2 cm × 20 cm, 25 g) with CH_2_Cl_2_/EtOAc (*v/v* 50:1, 40:1, 30:1, 20:1, 10:1, 5:1, 2:1, each 500 mL). Fr.9.1.3 was further purified by semi-preparative RP-HPLC (C18, MeOH/H_2_O, 85:15, 1.5 mL/min) to yield compound **11** (4.2 mg, *t*_R_ 69.5 min). Fr.9.1.5 (200 mg) was successively separated by reversed-phase silica gel CC (RP-18, 2 cm × 9 cm, 30 g) eluted with a gradient of MeOH/H_2_O (40:60, 50:50, 60:40, 70:30. 80:20, 90:10, 100:0, each 200 mL) to give Fr.9.1.5.1–Fr.9.1.5.8. Fr.9.1.5.6 (57.4 mg) was further purified by RP-HPLC (C18, MeOH/H_2_O, 80:20, 1.5 mL/min) to afford compounds **8** (9.8 mg, *t*_R_ 51.5 min) and **10** (1.3 mg, *t*_R_ 45.4 min), while Fr.9.1.5.7 (101.3 mg) was dealt with the same way by RP-HPLC (C8, MeOH/H_2_O, 84:16, 1.5 mL/min) to obtain compounds **9** (*t*_R_ 8.2 mg, 59.8 min,), **14** (6.4 mg, *t*_R_ 92.1 min), and **15** (20.9 mg, *t*_R_ 69.5 min). Fr.9.4 (664 mg) was chromatographed on silica gel CC (200–300 mesh, 1.5 cm × 20 cm, 13 g) with petroleum ether/acetone (*v/v* 60:1, 50:1, 40:1, 20:1, 10:1, 5:1, each 100 mL) as the eluent to give Fr.9.4.1–Fr.9.4.6. Fr.9.4.3 (244.6 mg) was further separated by reversed-phase silica gel CC (2 cm × 9 cm, 10 g) eluted with a gradient of MeOH/H_2_O (*v/v* 50:50, 60:40, 70:30, 80:20, 90:10, 100:0, each 100 mL) and then purified by HPLC (C8, MeOH/H_2_O, 79:21, 1.5 mL/min) to yield compounds **1** (8.4 mg, *t*_R_ 25.0 min), **2** (16.8 mg, *t*_R_ 52.3 min), **3** (2.1 mg, *t*_R_ 46.2 min), and **4** (5.5 mg, *t*_R_ 43.5 min).

*Paracaseolin A (1β,3β-dihydroxy betulin* (**1**): Irregular plates; mp 248–250 °C; ${\left[\alpha \right]}_{D}^{25}$ = 1.45 (*c* 0.46, CH_3_OH); IR (KBr): *ν*_max_ = 3359, 2925, 1681, 1457, 1373, 1180, 986, 881 cm^−1^; for ^1^H-NMR (DMSO, 500 MHz) and ^13^C-NMR (DMSO, 125 MHz) spectroscopic data, see Table 1; HR-ESI-MS (negative ion mode): *m*/*z* = 457.3688 [M − H]^−^ (calcd. for C_30_H_49_O_3_: 457.3676).

*Paracaseolin B (1β-hydroxy-3β-O-trans-p-coumaroyl betulin* (**2**): Fine needles (CHCl_3_-MeOH, 1:1); mp 264–266 °C; ${\left[\alpha \right]}_{D}^{25}$ = 28.03 (*c* 0.40, CH_3_OH); UV (CH_3_OH): *λ*_max_ (log ε) = 228 (2.58), 312 (2.85) nm; IR (KBr): *ν*_max_ = 3376, 2947, 1682, 1604, 1513, 1451, 1373, 1264, 1168, 1024, 831, 736 cm^−1^; for ^1^H-NMR (DMSO, 500 MHz) and ^13^C-NMR (DMSO, 125 MHz) spectroscopic data, see Table 1; HR-ESI-MS (negative ion mode): *m*/*z* = 603.4058 [M − H]^−^ (calcd. for C_39_H_55_O_5_: 603.4044).

*Paracaseolin C (1β-hydroxy-3β-O-cis-p-coumaroyl betulin* (**3**): White amorphous powder; mp 272–273 °C; ${\left[\alpha \right]}_{D}^{25}$ = 5.18 (*c* 0.09, CH_3_OH); UV (CH_3_OH): *λ*_max_ (log ε) = 228 (2.68), 310 (2.63) nm; IR (KBr): *ν*_max_ = 3287, 2927, 1695, 1602, 1512, 1452, 1372, 1165, 1023, 978, 881, 833, 736 cm^−1^; for ^1^H-NMR (DMSO, 500 MHz) and ^13^C-NMR (DMSO, 125 MHz) spectroscopic data, see Table 2; HR-ESI-MS (negative ion mode): *m*/*z* = 603.4056 [M − H]^−^ (calcd. for C_39_H_55_O_5_: 603.4044).

*Paracaseolin D (2α-hydroxy-3β-O-trans-p-coumaroyl betulin* (**4**): White amorphous powder; mp 280–281 °C; ${\left[\alpha \right]}_{D}^{25}$ = 4.00 (*c* 0.11, CH_3_OH); UV (CH_3_OH): *λ*_max_ (log ε) = 227 (2.70), 313 (2.78) nm; IR (KBr): *ν*_max_ = 3443, 2927, 1699, 1603, 1512, 1454, 1380, 1283, 1166, 1023, 960, 881, 831, 760 cm^−1^; for ^1^H-NMR (DMSO, 500 MHz) and ^13^C-NMR (DMSO, 125 MHz) spectroscopic data, see Table 2; HR-ESI-MS (negative ion mode): *m*/*z* = 603.4056 [M − H]^−^ (calcd. for C_39_H_55_O_5_: 603.4044).

*Paracaseolin E (2α-hydroxy-3β-O-trans-p-coumaroyl dulcioic acid* (**11**): White amorphous powder; mp 282–284 °C; ${\left[\alpha \right]}_{D}^{25}$= 14.77 (*c* 0.16, CH_3_OH); UV (CH_3_OH): *λ*_max_ (log ε) = 225 (2.60), 311 (2.74) nm; IR (KBr): *ν*_max_ = 3485, 2927, 1710, 1686, 1603, 1510, 1457, 1282, 1167, 1025, 962, 880, 832, 745 cm^−1^; for ^1^H-NMR (DMSO, 600 MHz) and ^13^C-NMR (DMSO, 150 MHz) spectroscopic data, see Table 2; HR-ESI-MS (negative ion mode): *m*/*z* = 617.3850 [M − H]^−^ (calcd. for C_39_H_55_O_5_: 617.3837).

### 3.4. Cytotoxicity Assay

All the cell lines were purchased from Shanghai Institute of Cell Biology (Shanghai, China). A549, P388, and K562 cell lines were grown in RPMI 1640 while HeLa cell line was maintained in DMEM. Each medium contained 10% fetal bovine serum (FBS) and 1% penicillin-streptomycin. These cell lines were incubated in a humidified atmosphere with 5% CO_2_ at 37 °C.

Well-growing carcinoma cells were collected and seeded in 96-well plates at 1 × 10^5^/mL density used for each sample. Samples diluted to a gradient concentration with DMSO were added into the cells at final concentrations of 0, 1.5625, 3.125, 6.25, 12.5, and 50 µM, respectively, with three duplicate wells for each group. To the control group was added DMSO. After incubation at 37 °C, 5% CO_2_ for 48 h, cytotoxicities were determined by MTT (3-(4,5-dimethylthiazol-2-yl)-2,5-diphenyltetrazolium bromide) colorimetric assay [28] against K562 (human leukemia cells) and P388 (mouse leukemia cells), and SRB (Sulforhodamine B) assay [29] against HeLa (human cervical carcinoma cells) and A549 (human lung carcinoma cells). Adriamycin (doxorubicin, ADM, purity >98%, LuKang Cisen, Jining, China) was used as a positive control, and IC_50_ values >50 µM were considered to be inactive in cytotoxic assays.

### 3.5. Anti-H1N1 Virus Assay

The antiviral activity against H1N1 was evaluated by the CPE inhibition assay [27]. Confluent MDCK cell monolayers were firstly incubated with influenza virus (A/Puerto Rico/8/34 (H1N1), PR/8) at 37 °C for 1 h. After removing the virus dilution, cells were maintained in infecting media (RPMI 1640, 4 µg/mL of trypsin) containing different concentrations of test compounds at 37 °C. After 48 h incubation at 37 °C, cells were fixed with 100 µL of 4% formaldehyde for 20 min at room temperature. After removal of the formaldehyde, the cells were stained with 0.1% crystal violet for 30 min. The plates were washed and dried, and the intensity of crystal violet staining for each well was measured in a microplate reader (Bio-Rad, Hercules, CA, USA) at 570 nm. The IC_50_ was calculated as the compound concentration required inhibiting CPE production at 48 h post-infection by 50% Ribavirin (LuKang Cisen, Jining, China, purity > 98%) was used as positive control, and compounds with an inhibition rate of >70%, >50%, and <30% at 50 µg/mL were, respectively, regarded as having strong, moderate, and weak activities.

## 4. Conclusions

Seventeen triterpenoids, including four new lupane derivatives (**1**–**4**), one new ursane derivative (**11**), as well as twelve known oleanane and cycloartane derivatives (**5**–**10**, **12**–**17**), were isolated from the aerial parts of *Sonneratia paracaseolaris* for the first time. Compound **4** showed potent cytotoxicity against the A549 cell line, and compound **1** exhibited significant anti-H1N1 virus activity. Furthermore, the preliminary structure activity relationship analysis suggested the importance of the *O-p*-coumaroyl group at C-3 with respect to cytotoxicities. This study indicates that the plant *Sonneratia paracaseolaris* is rich in structurally-diverse triterpenoids with potential activities and is worthy of further investigation.

## Figures and Tables

**Table 1:** ^1^H- and ^13^C-NMR spectroscopic data for compounds **1**–**4** ^a^ (δ in ppm, DMSO, *J* in Hz).

Compounds	1 ^b,c^			2 ^b^			3 ^b^			4 ^b^		
No.	δ_H_	δ_C_		δ_H_	δ_C_		δ_H_	δ_C_		δ_H_	δ_C_	
**1**	3.14 m (W_1/2_ 22.1)	77.9	d	3.30 dd (10.8, 4.7)	77.2	d	3.27 ^d^	77.2	d	1.87 m 0.84m	47.7	d
**2**	1.47 m	38.5	t	1.68 m	34.0	t	1.68 m	33.8	t	3.67 m (W_1/2_ 26.6 )	64.9	t
**3**	2.96 m (W_1/2_ 22.8)	74.0	d	4.47 dd (11.9, 4.7)	76.7	d	4.40 dd (12.5, 4.5)	76.9	d	4.48 d (10.0 )	83.4	d
**4**		38.0	s		37.6	s		37.4	s		39.1	s
**5**	0.46 d (11.0)	52.6	d	0.68 m	52.2	d	0.65 m	52.2	d	0.86 m	54.5	d
**6**	1.38 m	17.6	t	1.43 m	17.4	t	1.43 m	17.4	t	1.28 m	17.8	t
**7**	1.28 m	33.9	t	1.28 m	33.8	t	1.28 m	33.8	t	1.40 m 1.29 m	33.6	t
**8**		41.0	t		41.0	t		41.0	t		40.5	t
**9**	1.36 m	51.0	d	1.44 m	50.7	d	1.43 m	50.7	d	1.32 m	49.6	d
**10**		42.9	s		42.8	s		42.8	s		37.7	s
**11**	1.13 m 2.35 m	23.1	t	1.15 m 2.35 m	23.0	t	1.11 m 2.32 m	23.0	t	1.29 m 2.34 m	20.5	t
**12**	0.87 m 1.48 m	25.1	d	0.90 m 1.49 m	25.0	d	0.97 m 1.49 m	25.1	d	0.93 m 1.60 m	24.7	d
**13**	1.59 m	36.5	d	1.59 m	36.5	d	1.60 m	36.5	d	1.60 m	36.7	d
**14**		42.3	s		42.3	s		42.3	s		42.3	s
**15**	1.57 m	26.7	t	1.61 m	26.8	t	1.61 m	26.7	t	1.62 m	26.6	t
**16**	1.01 m 1.88 m	29.1	t	1.04 m 1.88 m	29.1	t	1.02 m 1.88 m	29.1	t	1.01 m 1.88 m	29.0	t
**17**		47.3	s		47.3	s		47.3	s		47.3	s
**18**	1.45 m	48.2	d	1.44 m	48.2	d	1.46 m	48.2	d	1.46 m	48.1	d
**19**	2.36 m	47.3	d	2.36 m	47.3	d	2.36 m	47.3	d	2.35 m	47.4	d
**20**		150.4	s		150.4	s		150.4	s		150.3	s
**21**	1.22 m 1.83 m	29.3	t	1.22 m 1.83 m	29.3	t	1.24 m 1.84 m	29.2	t	1.24 m 1.84 m	29.3	t
**22**	0.87 m 1.85 m	33.8	t	0.86 m 1.85 m	33.7	t	0.83 m1.85 m	33.7	t	0.84 m 1.85 m	33.8	t
**23**	0.83 s	28.0	q	0.77 s	27.6	q	0.76 s	27.5	q	0.78 s	28.3	q
**24**	0.61 s	15.4	q	0.84 s	16.0	q	0.71 s	16.0	q	0.83 s	17.6	q
**25**	0.76 s	12.2	q	0.84 s	12.2	q	0.81 s	12.1	q	0.87 s	17.0	q
**26**	0.98 s	16.0	q	1.00 s	16.2	q	0.99 s	16.1	q	0.99 s	15.7	q
**27**	0.93 s	14.5	q	0.96 s	14.5	q	0.95 s	14.5	q	0.96 s	14.4	q
**28**	3.07 d (10.8)	58.0	t	3.07 d (10.7)	58.0	t	3.07 d (10.8)	57.9	t	3.07 d (10.8)	57.9	t
	3.51 d (10.8)			3.51 d (10.7)			3.51 d (10.8)			3.51 d (10.8)		
**29**	4.53 s 4.66 s	109.6	t	4.54 s 4.67 s	109.5	t	4.54 s 4.67 s	109.6	t	4.55 s 4.68 s	109.6	t
**30**	1.63 s	18.7	q	1.64 s	18.7	q	1.64 s	18.7	q	1.65 s	18.8	q
**1′**					160.0	s		159.6	s		159.9	s
**2′,6′**				7.53 d (8.6)	130.3	d	7.60 d (8.6)	132.4	d	7.49 d (8.6)	130.1	d
**3′,5′**				6.77 d (8.6)	115.8	d	6.72 d (8.6)	115.0	d	6.74 d (8.6)	116.0	d
**4′**					124.9	s		125.1	s		124.3	s
**7′**				7.51 d (16.0)	144.5	d	6.82 d (13.0)	143.1	d	7.51 d (16.2)	144.2	d
**8′**				6.33 d (16.0)	114.4	d	5.73 d (13.0)	115.0	d	6.32 d (16.2)	114.4	d
**9′**					166.2	s		165.8	s		166.7	s

^a^ Assignments were based on 1D and 2D NMR experiments (COSY, HMBC, HSQC, and NOESY) and recorded in DMSO; ^b 1^H-, ^13^C-NMR, DEPT and 2D NMR spectra were recorded on a Varian 500 NMR; ^c^ 1-OH 4.02 d (5.3); 3-OH 4.28 d (5.0); 28-OH 4.20 br s; ^d^ overlapped.

**Table 2:** ^1^H- and ^13^C-NMR spectroscopic data for compound **11** ^a^ (δ in ppm, DMSO, *J* in Hz).

Compound	11 ^b^		
No.	δ_H_	δ_C_	
**1**	1.88 m 0.95 m	47.6	t
**2**	3.69 m (W_1/2_ 22.9)	64.8	d
**3**	4.51 d (9.9)	83.6	d
**4**		39.8	s
**5**	0.93 m	54.4	d
**6**	1.47 m 1.88 m	17.9	t
**7**	1.54 m	32.5	t
**8**		41.7	t
**9**	1.56 m	46.8	d
**10**		37.5	s
**11**	1.89 m	23.9	t
**12**	5.15 br s	125.1	d
**13**		138.4	s
**14**		41.7	s
**15**	1.80 m	27.5	t
**16**	1.53 m	23.0	t
**17**		30.3	s
**18**	2.12 d (11.0)	52.4	d
**19**	1.32 m	38.5	d
**20**	1.56 m	38.5	d
**21**	1.91 m	36.4	t
**22**	1.80 m	39.6	t
**23**	0.80 s	28.5	q
**24**	0.85 s	17.1	q
**25**	0.97 s	17.1	q
**26**	0.76 s	16.4	q
**27**	1.07 s	23.2	q
**28**	0.92 s	21.1	q
**29**	0.83 d (6.1)	23.2	q
**30**		181.4	s
**1′**		125.1	s
**2′,6′**	7.55 d (8.8)	130.2	d
**3′,5′**	6.79 d (8.8)	115.8	d
**4′**		159.8	s
**7′**	7.52 d (16.4)	144.1	d
**8′**	6.38 d (16.4)	115.0	d
**9′**		166.7	s

^a^ Assignments were based on 1D and 2D NMR experiments (COSY, HMBC, HSQC, and NOESY) and recorded in DMSO; ^b^ 1H-, 13C-NMR, DEPT and 2D NMR spectra were recorded on a JEOL JNM-ECP 600 NMR.

**Table 3:** Cytotoxic activities of compounds **1**–**17** (IC_50_, µM).

Compounds	P388 ^a^	HeLa ^b^	A549 ^b^	K562 ^a^
**1**	>50	>50	>50	>50
**2**	27.25	>50	>50	>50
**3**	22.39	33.20	14.43	>50
**4**	10.56	19.13	1.89	>50
**5**	>50	>50	>50	>50
**6**	44.40	42.46	>50	>50
**7**	41.97	>50	>50	>50
**8**	34.38	>50	27.49	>50
**9**	22.36	27.15	15.43	>50
**10**	39.03	>50	37.32	>50
**11**	>50	30.41	>50	>50
**12**	39.77	>50	18	>50
**13**	>50	>50	23.90	>50
**14**	11.04	13.10	>50	>50
**15**	23.04	24.90	>50	16.28
**16**	>50	>50	>50	>50
**17**	>50	>50	>50	>50
**ADM (Adriamycin) ^c^**	0.3	0.6	0.2	0.2

^a^ By MTT method. ^b^ By SRB method. ^c^ Positive control.

## Supplementary Materials

The 1D, 2D NMR, and MS spectra for compounds **1**–**4** and **11** (Figures S1–S49) are available online.
