# Supplementary material for: Cytotoxic and Antiviral Triterpenoids from the Mangrove Plant Sonneratia paracaseolaris
Source: Molecules. 2017 Aug 9;22(8):1319. doi: 10.3390/molecules22081319 (PMC6152125; doi:10.3390/molecules22081319)
Supplement: Supplementary file 1 [file molecules-22-01319-s001.pdf]

Supporting Information

Triterpenoids from the Mangrove Plant *Sonneratia Paracaseolaris* and Their Bioactivities

Kai-Kai Gong[1,3,†], Ping-Lin Li[1,†], Dan Qiao[1], Xing-Wang Zhang[1], Mei-Jun Chu[1], Guo-Fei Qin[1], Xu-Li Tang[2,*] and Guo-Qiang Li [1,*]

[1] Key Laboratory of Marine Drugs, Chinese Ministry of Education, School of Medicine and Pharmacy, Ocean University of China, Qingdao 266003, China; gongkaikai1005@163.com (K.-K.G.); lipinglin@ouc.edu.cn (P.-L.L.); hdqiaodan@126.com (D.Q.); 735888678@qq.com (X.-W.Z.); chumjun@163.com (M.-J.C.); 806722433@qq.com (G.-F.Q.);

[2] College of Chemistry and Chemical Engineering, Ocean University of China, Songling Road 238, Qingdao 266100, China;

[3] Cancer Research institute, Binzhou Medical University Hospital, Yellow river second Road 661, Binzhou 256603, China;

* Correspondence: tangxuli@ouc.edu.cn (X.-L.T.); liguoqiang@ouc.edu.cn (G.-Q.L.); Tel.: +86-532-8203-2323; fax: +86-532-8203-3054.

† These authors contributed equally to this work.

List of Supplemental Data

1. Experimental Section

2. 1D and 2D NMR data

No.	Contents	page
Figure 1S.	The negtive HRESIMS spectrum of compound **1**	7
Figure 2S.	^{1}H-NMR (500M, DMSO) spectrum of compound **1**	8
Figure 3S.	The amplificatory ^{1}H-NMR spectrum of compound **1**	9
Figure 4S.	^{13}C-NMR (500M, DMSO) spectrum of compound **1**	10
Figure 5S.	DEPT (500M, DMSO) spectrum of compound **1**	11
Figure 6S.	^{1}H -^{1}H COSY spectrum of compound **1**	12
Figure 7S.	HMQC spectrum of compound **1**	13
Figure 8S.	HMBC spectrum of compound **1**	14
Figure 9S.	NOESY spectrum of compound **1**	15
Figure 10S.	The negtive HRESIMS spectrum of compound **2**	16
Figure 11S.	^{1}H-NMR (500M, DMSO) spectrum of compound **2**	17
Figure 12S.	The amplificatory ^{1}H-NMR (500M, DMSO) spectrum of compound **2**	18
Figure 13S.	^{13}C-NMR (500M, DMSO) spectrum of compound **2**	19
Figure 14S.	DEPT (500M, DMSO) spectrum of compound **2**	20
Figure 15S.	^{1}H -^{1}H COSY spectrum of compound **2**	21
Figure 16S.	HMQC spectrum of compound **2**	22
Figure 17S.	HMBC spectrum of compound **2**	23
Figure 18S.	NOESY spectrum of compound **2**	24
Figure 19S.	The negtive HRESIMS spectrum of compound **3**	25
Figure 20S.	^{1}H-NMR (500M, DMSO) spectrum of compound **3**	26
Figure 21S.	The amplificatory ^{1}H-NMR (500M, DMSO) spectrum of compound **3**	27
Figure 22S.	^{13}C-NMR (500M, DMSO) spectrum of compound **3**	28
Figure 23S.	DEPT (500M, DMSO) spectrum of compound **3**	29
Figure 24S.	^{1}H -^{1}H COSY spectrum of compound **3**	30
Figure 25S.	HMQC spectrum of compound **3**	31
Figure 26S.	HMBC spectrum of compound **3**	32
Figure 27S.	NOESY spectrum of compound **3**	33

Figure 28S.	The negtive HRESIMS spectrum of compound **4**	34
Figure 29S.	^{1}H-NMR (500M, DMSO) spectrum of compound **4**	35
Figure 30S.	The amplificatory ^{1}H-NMR (500M, DMSO) spectrum of compound **4**	36
Figure 31S.	^{13}C-NMR (500M, DMSO) spectrum of compound **4**	37
Figure 32S.	DEPT (500M, DMSO) spectrum of compound **4**	38
Figure 33S.	^{1}H -^{1}H COSY spectrum of compound **4**	39
Figure 34S.	HMQC spectrum of compound **4**	40
Figure 35S.	HMBC spectrum of compound **4**	41
Figure 36S.	NOESY spectrum of compound **4**	42
Figure 37S.	^{1}H-NMR (500M, $CDCl_3$) spectrum of compound **12**	43
Figure 38S.	The amplificatory ^{1}H NMR (500M, $CDCl_3$) spectrum of compound **12**	44
Figure 39S.	^{1}H-NMR (600M, DMSO) spectrum of compound **12**	45
Figure 40S.	The amplificatory ^{1}H NMR (600M, DMSO) spectrum of compound **12**	46
Figure 41S.	The negtive HRESIMS spectrum of compound **11**	47
Figure 42S.	^{1}H-NMR (600M, DMSO) spectrum of compound **11**	48
Figure 43S.	The amplificatory ^{1}H-NMR (600M, DMSO) spectrum of compound **11**	49
Figure 44S.	^{13}C-NMR (600M, DMSO) spectrum of compound **11**	50
Figure 45S.	DEPT (600M, DMSO) spectrum of compound **11**	51
Figure 46S.	^{1}H -^{1}H COSY spectrum of compound **11**	52
Figure 47S.	HMQC spectrum of compound **11**	53
Figure 48S.	HMBC spectrum of compound **11**	54
Figure 49S.	NOESY spectrum of compound **11**	55

1. Experimental section

***Animal material*:**

The aerial parts of *Sonneratia Paracaseolaris* were collected in Wenchang, Hainan Province, China, in October 2007, and was identified by Associate Prof. Cairong Zhong (Dongzhai Mangrove Forest National Nature Reserve). The voucher specimen (NO. WC-2007-10) was deposited at State Key Laboratory of Marine Drugs, Ocean University of China, China.

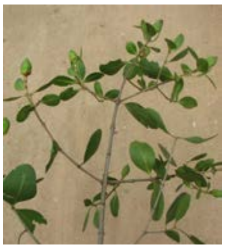

S. paracaseolaris

Biological assay:

Table 1. Inhibition rates of compounds **1–17** in preliminary cytotoxicity test.

Compounds	P388		HeLa		A549		K562	
	Inhibition ratio (%)	OD value	Inhibition ratio (%)	OD value	Inhibition ratio (%)	OD value	Inhibition ratio (%)	OD value
Adramycin	80.54 (1μM)	0.38±0.00	67.43 (1μM)	0.74±0.04	60.52 (0.5μM)	0.74±0.06	80.16 (0.5μM)	0.34±0.03
**1** (50μM)	59.48	0.76±0.00	29.34	1.54±0.06	42.71	1.04±0.05	6.04	1.63±0.05
**2** (50μM)	60.81	0.73±0.02	52.25	1.04±0.03	38.06	1.12±0.07	37.08	1.09±0.02
**3** (50μM)	80.00	0.37±0.02	51.72	1.05±0.03	43.76	1.02±0.05	36.08	1.11±0.02
**4** (50μM)	80.67	0.36±0.03	77.31	0.49±0.02	36.51	1.15±0.05	18.95	1.41±0.02
**5** (50μM)	-2.04	1.56±0.05	16.62	1.52±0.02	1.40	2.48±0.04	-5.46	1.83±0.02
**6** (50μM)	73.68	0.49±0.02	51.77	1.05±0.05	39.83	1.09±0.05	18.15	1.42±0.03
**7** (50μM)	64.85	0.66±0.01	20.83	1.72±0.03	55.70	0.80±0.07	-1.23	1.76±0.04
**8** (50μM)	72.27	0.52±0.01	51.36	1.06±0.01	62.00	0.69±0.04	47.02	0.92±0.02
**9** (50μM)	80.35	0.37±0.00	81.47	0.40±0.03	56.69	0.78±0.01	75.86	0.42±0.02
**10** (50μM)	77.37	0.42±0.02	44.05	1.22±0.06	66.46	0.61±0.04	0.54	1.72±0.05
**11** (50μM)	73.72	0.49±0.02	67.94	0.70±0.05	-3.99	1.88±0.05	8.40	1.59±0.05

**12** (50μM)	80.42	0.37±0.01	31.35	1.49±0.03	50.27	0.90±0.05	41.45	1.02±0.05
**13** (50μM)	52.36	0.89±0.04	17.13	1.80±0.05	30.29	1.26±0.03	2.23	1.7±0.04
**14** (50μM)	81.78	0.34±0.01	84.31	0.34±0.06	22.84	1.39±0.01	61.51	0.67±0.01
**15** (50μM)	80.89	0.36±0.01	77.33	0.49±0.00	28.68	1.29±0.04	86.93	0.23±0.01
**16** (50μM)	14.11	1.60±0.01	-1.31	2.21±0.04	45.98	0.98±0.01	-3.08	1.79±0.03
**17** (50μM)	3.59	1.80±0.03	51.72	1.05±0.03	3.69	1.74±0.03	-3.17	1.79±0.03

Table 2. Inhibition rates of compounds with anti-H1N1 virus activities in preliminary test.

Compounds	Concentration (μg/mL)	Inhibition ratio (%)
Ribavirin	50	72.4
**1**	50	61.3
**3**	50	7.0
**4**	50	44.9
**5**	50	45.7
**6**	50	10.1
**7**	50	27.6
**10**	50	33.7
**16**	50	28.2
**17**	50	42.5

2. 1D and 2D NMR data

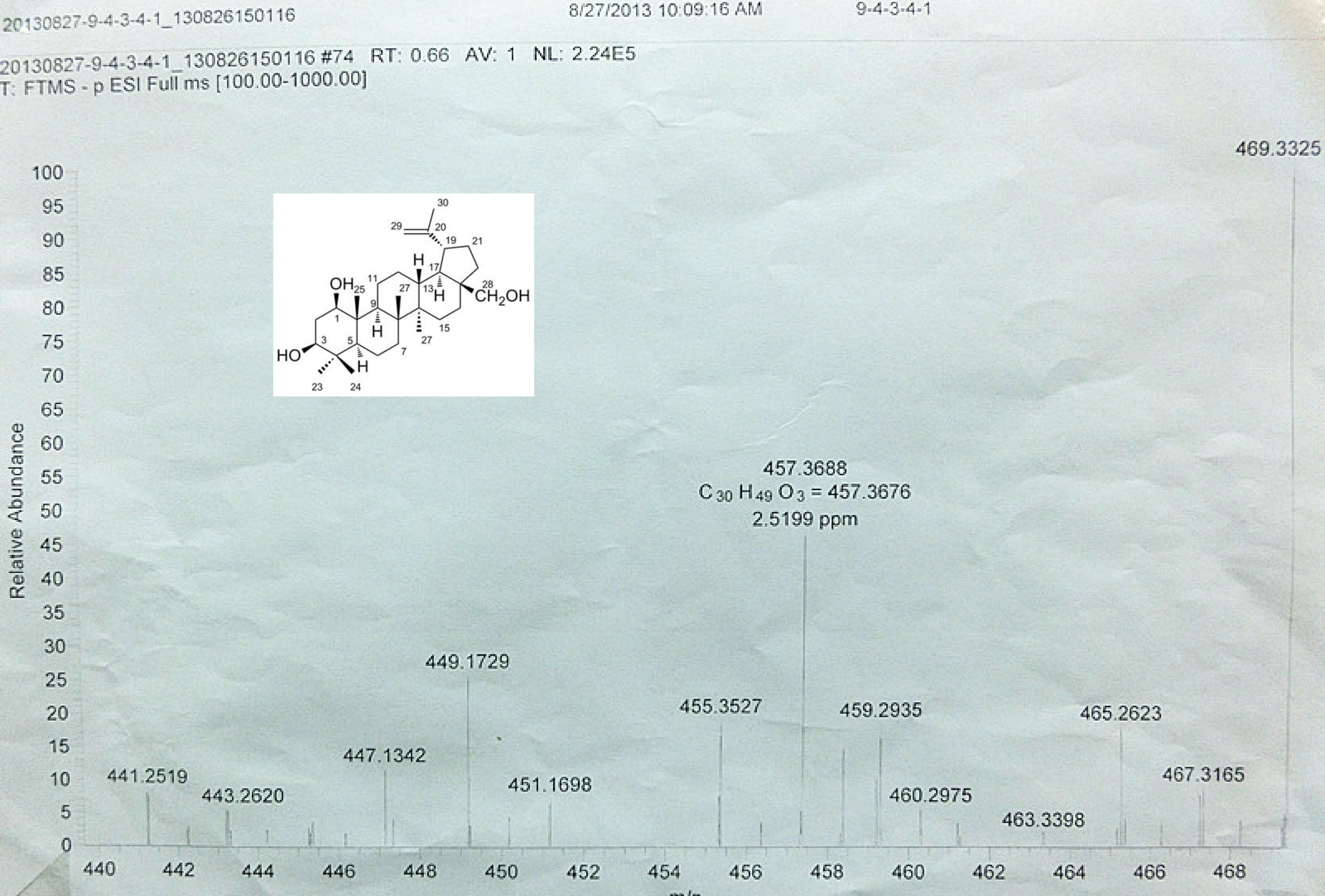

Figure 1S. The negtive HRESIMS spectrum of compound 1

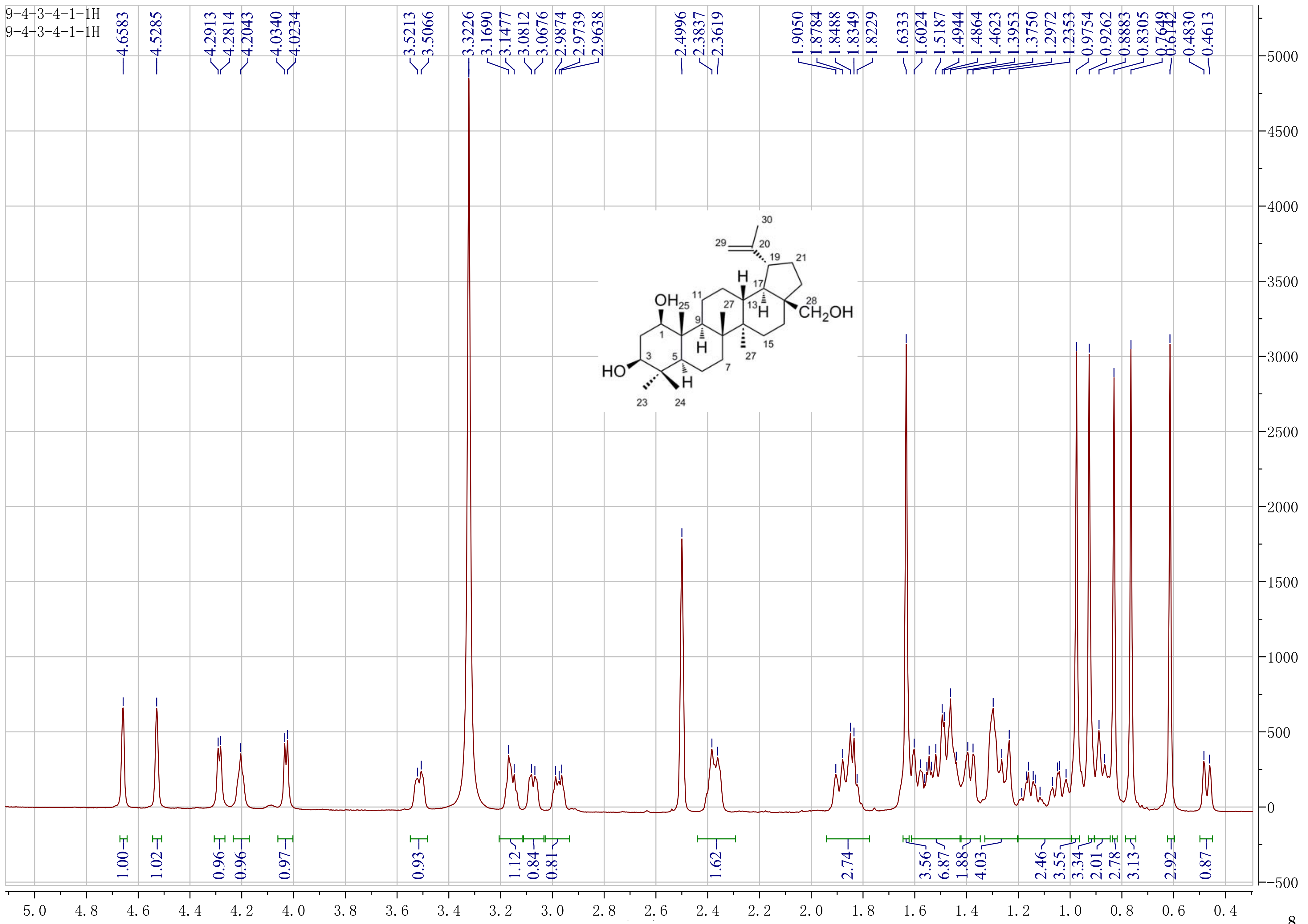

Figure 2S. ^{1}H-NMR (500M, DMSO) spectrum of compound **1**

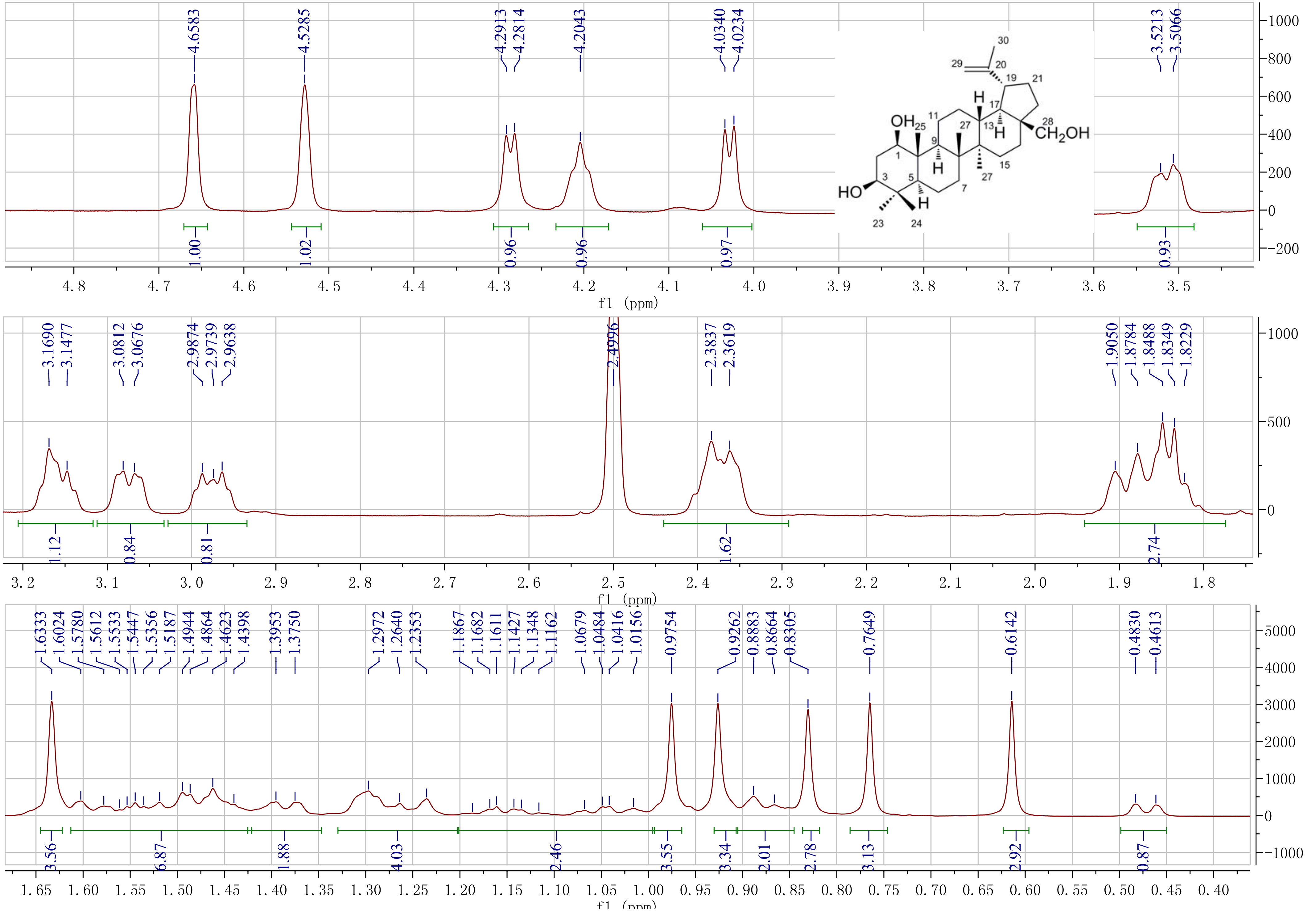

Figure 3S.The amplificatory ^{1}H-NMR spectrum of compound **1**

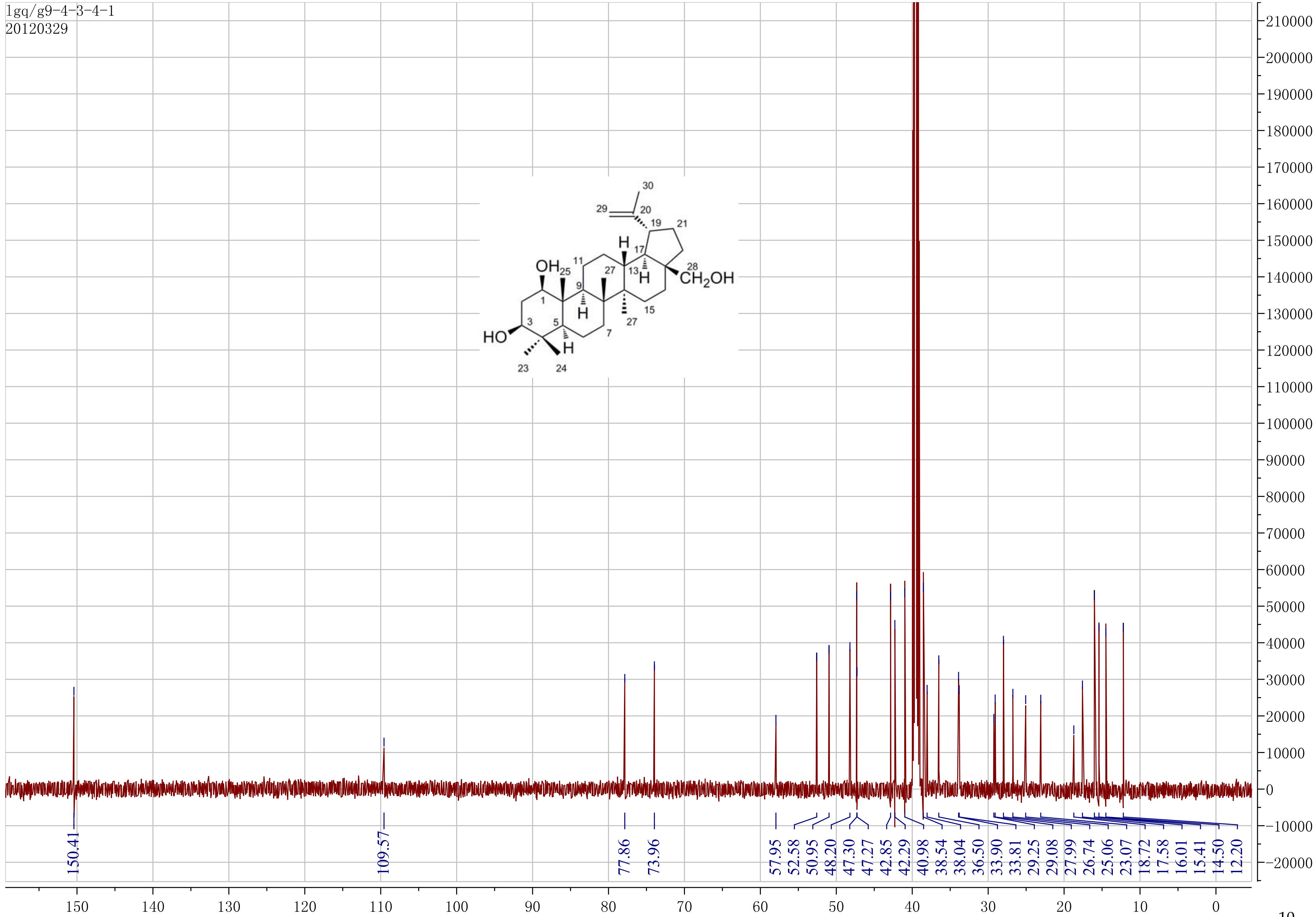

Figure 4S. ^{13}C-NMR (500M, DMSO) spectrum of compound **1**

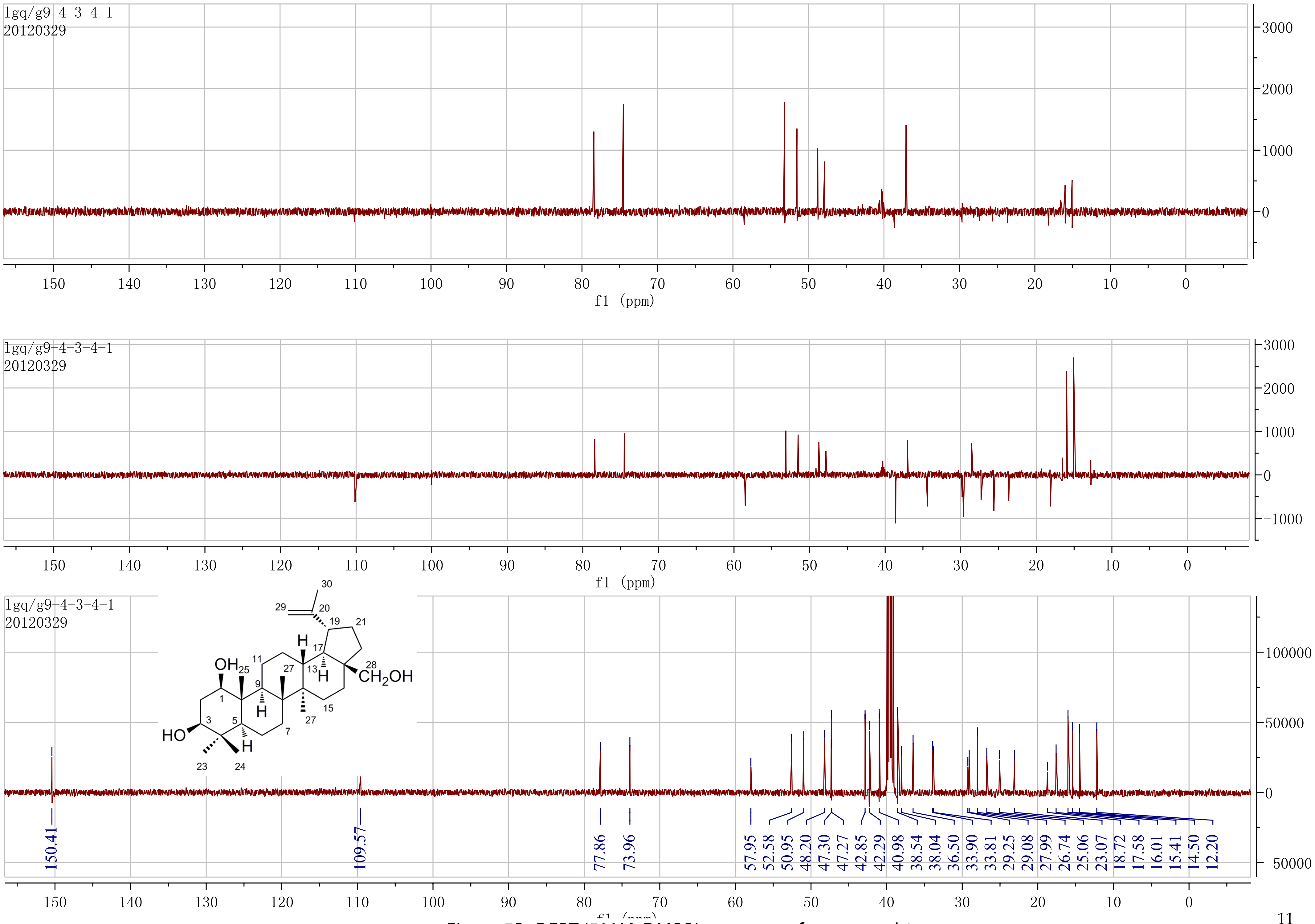

Figure 5S. DEPT (500M, DMSO) spectrum of compound 1

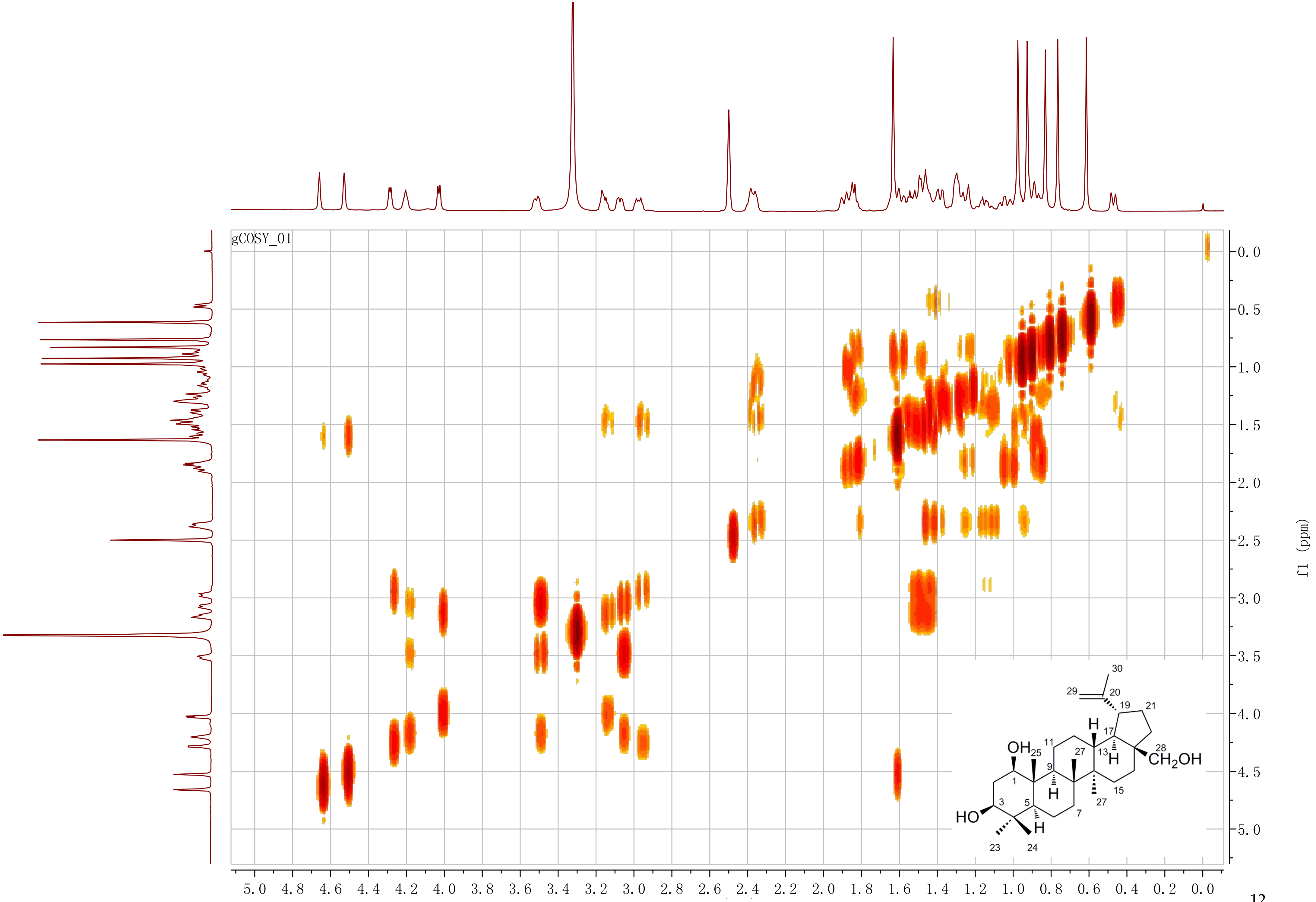

Figure 6S. ^{1}H -^{1}H COSY spectrum of compound 1

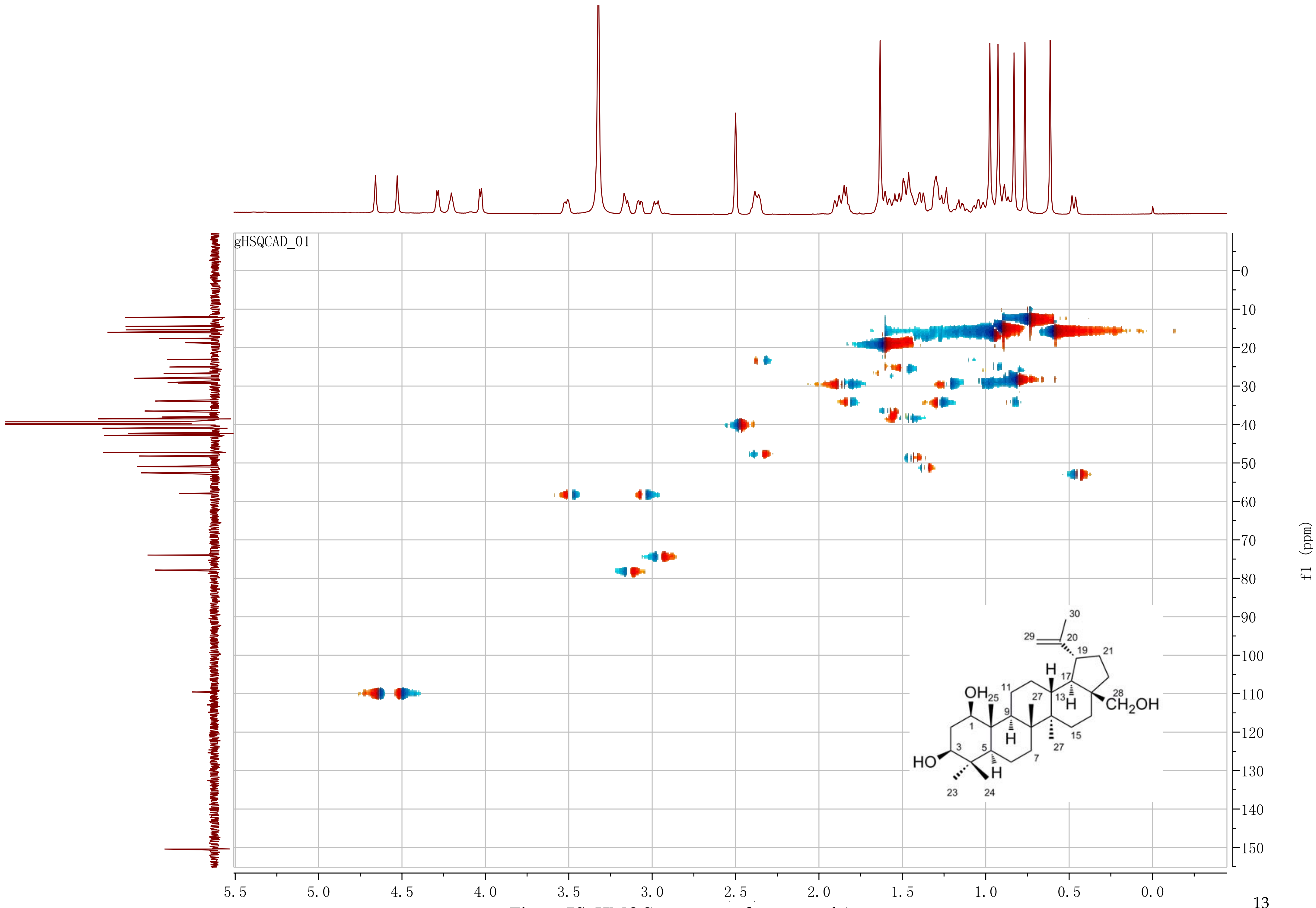

Figure 7S. HMQC spectrum of compound **1**

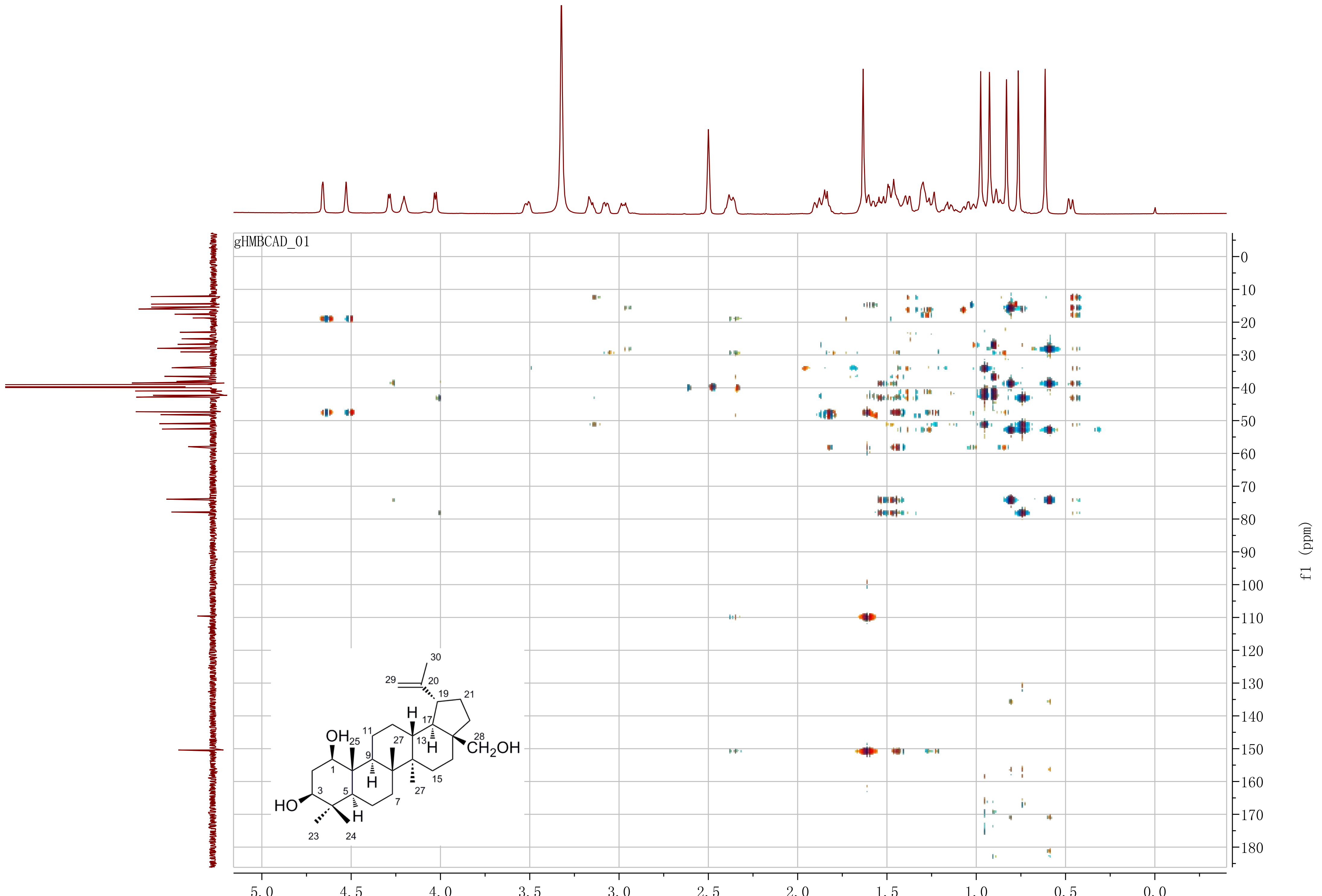

Figure 8S. HMBC spectrum of compound **1**

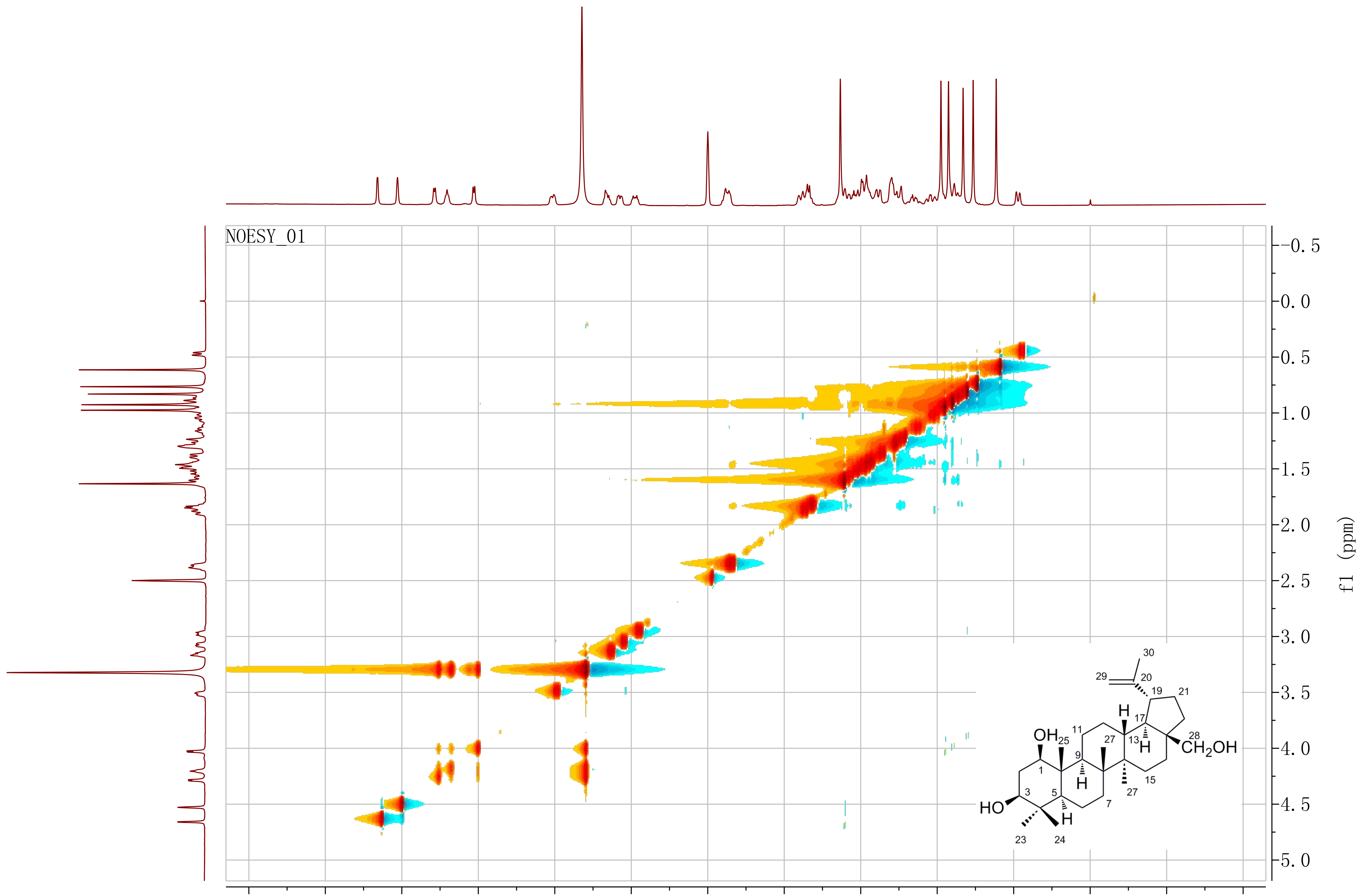

Figure 9S. NOESY spectrum of compound **1**

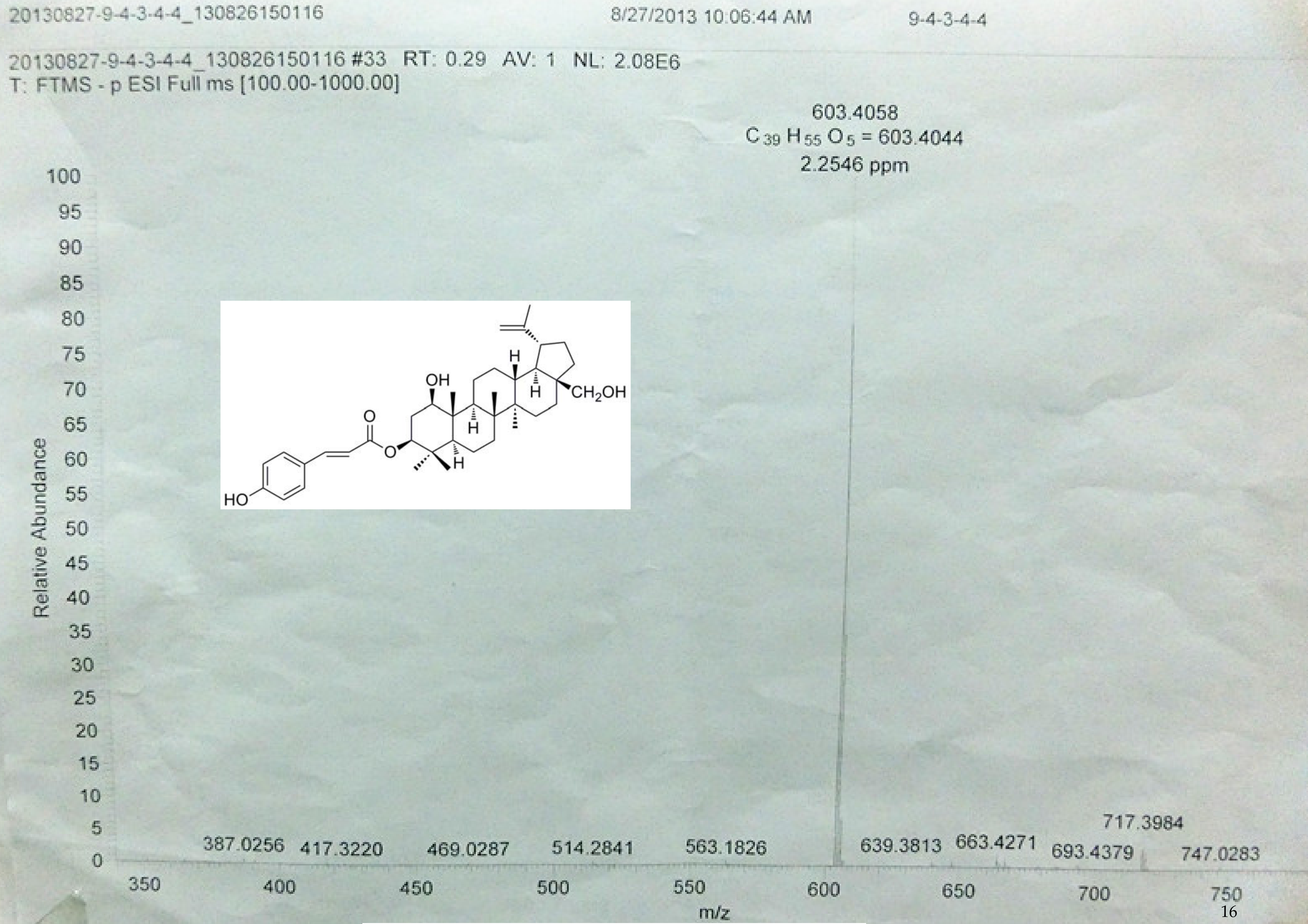

Figure 10S. The negative HRESIMS spectrum of compound 2

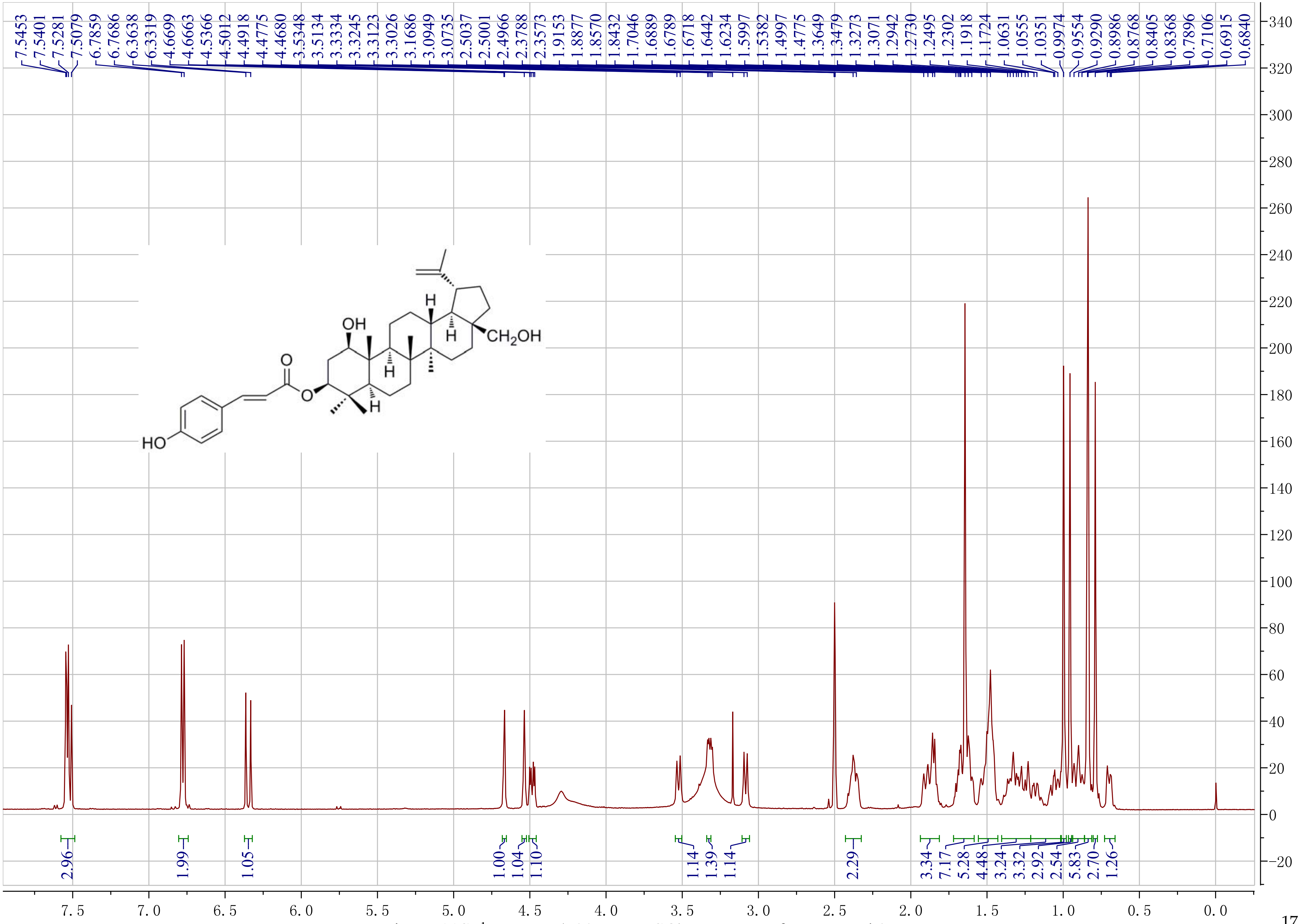

Figure 11S. ^{1}H-NMR (500M, DMSO) spectrum of compound **2**

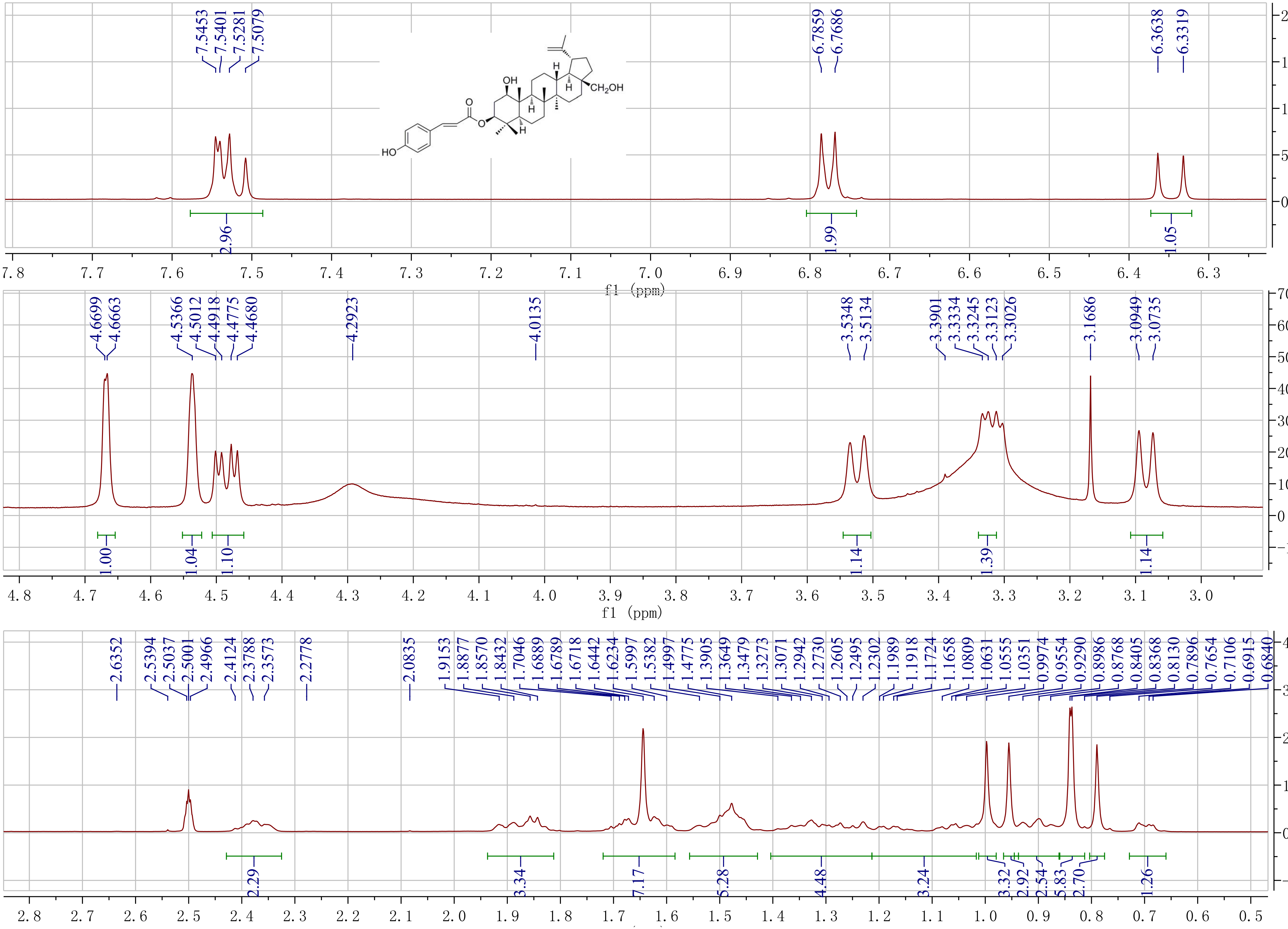

Figure 12S. The amplificatory ^{1}H-NMR (500M, DMSO) spectrum of compound **2**

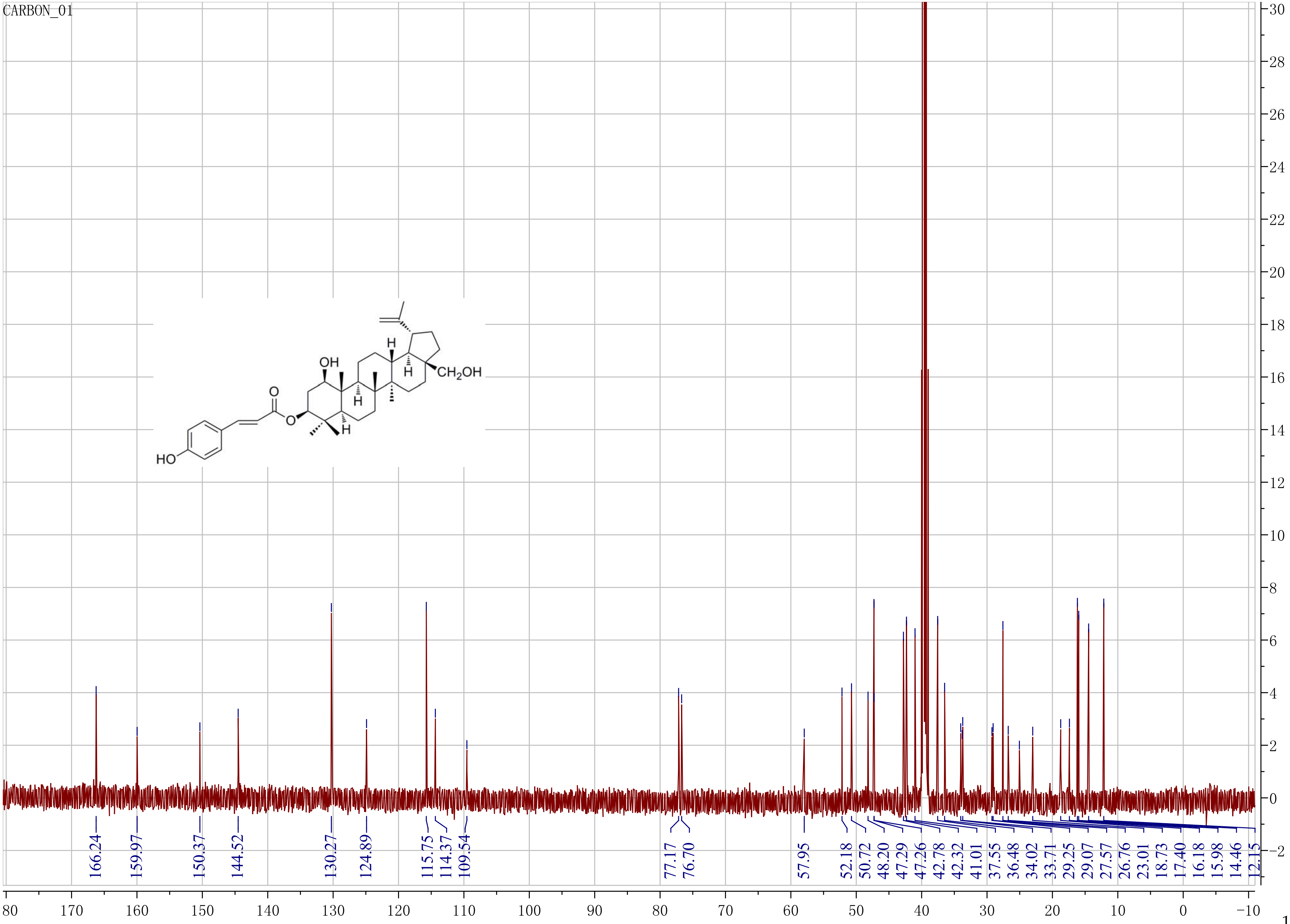

Figure 13S. ^{13}C-NMR (500M, DMSO) spectrum of compound **2**

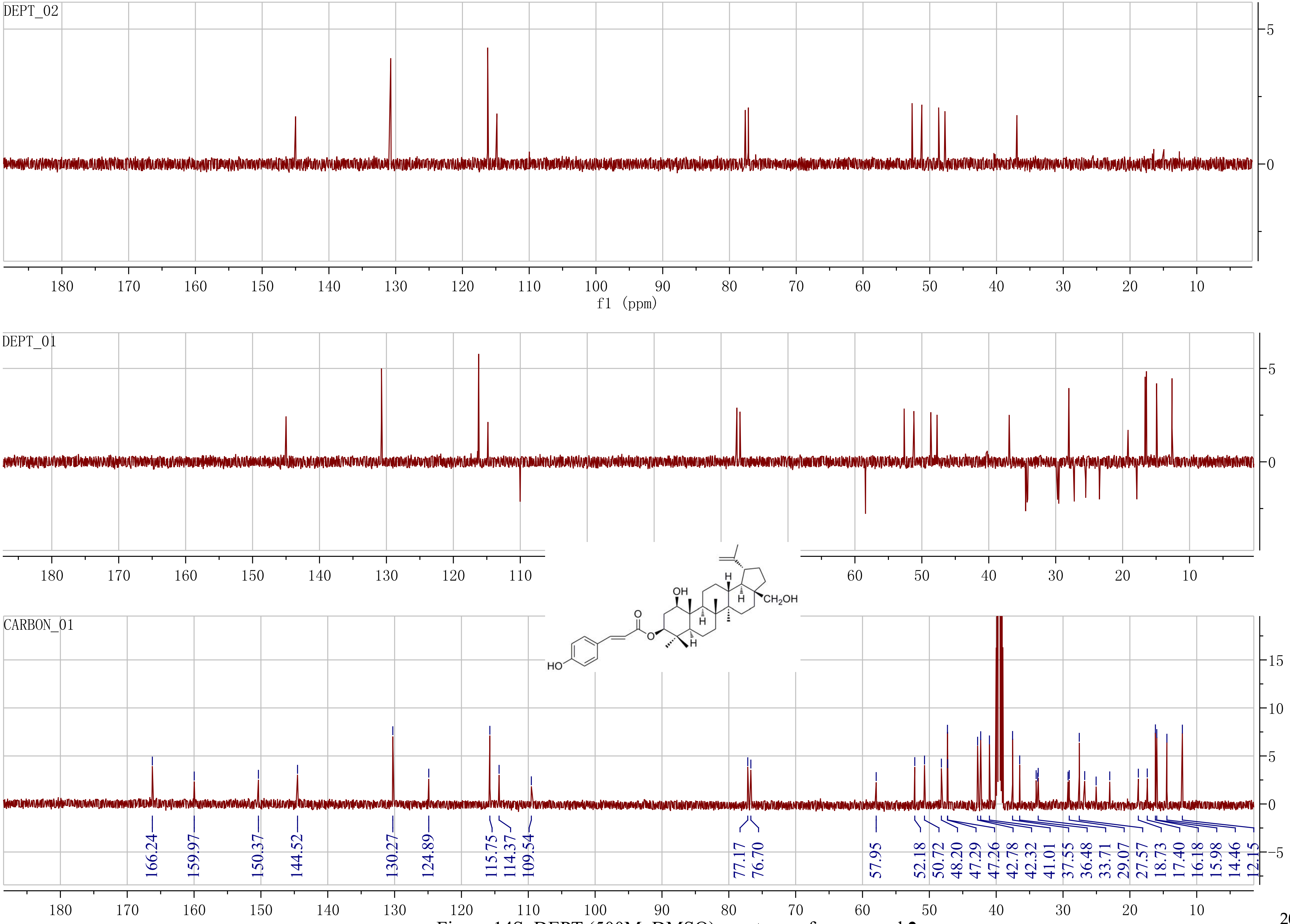

Figure 14S. DEPT (500M, DMSO) spectrum of compound **2**

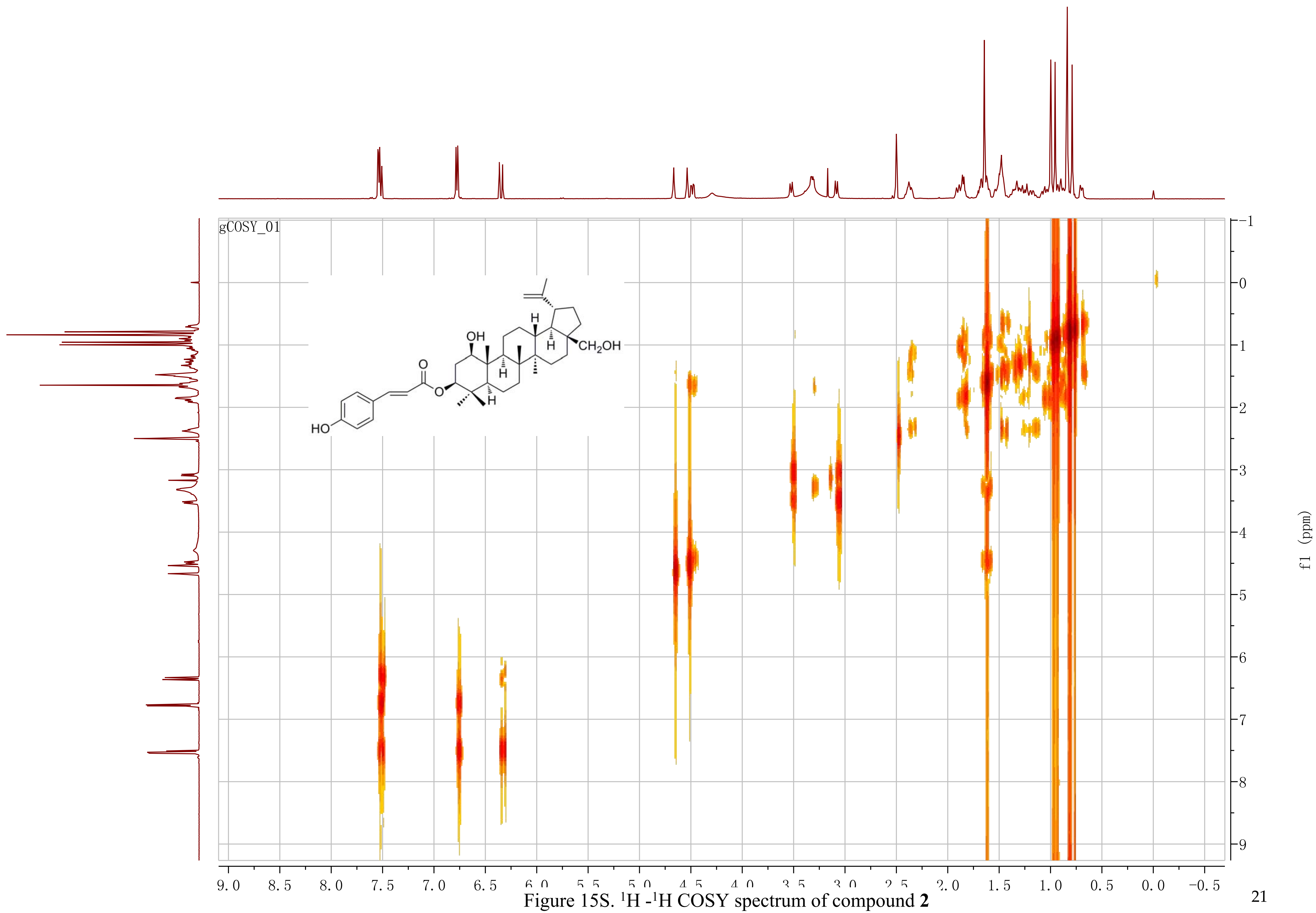

Figure 15S. ^{1}H -^{1}H COSY spectrum of compound **2**

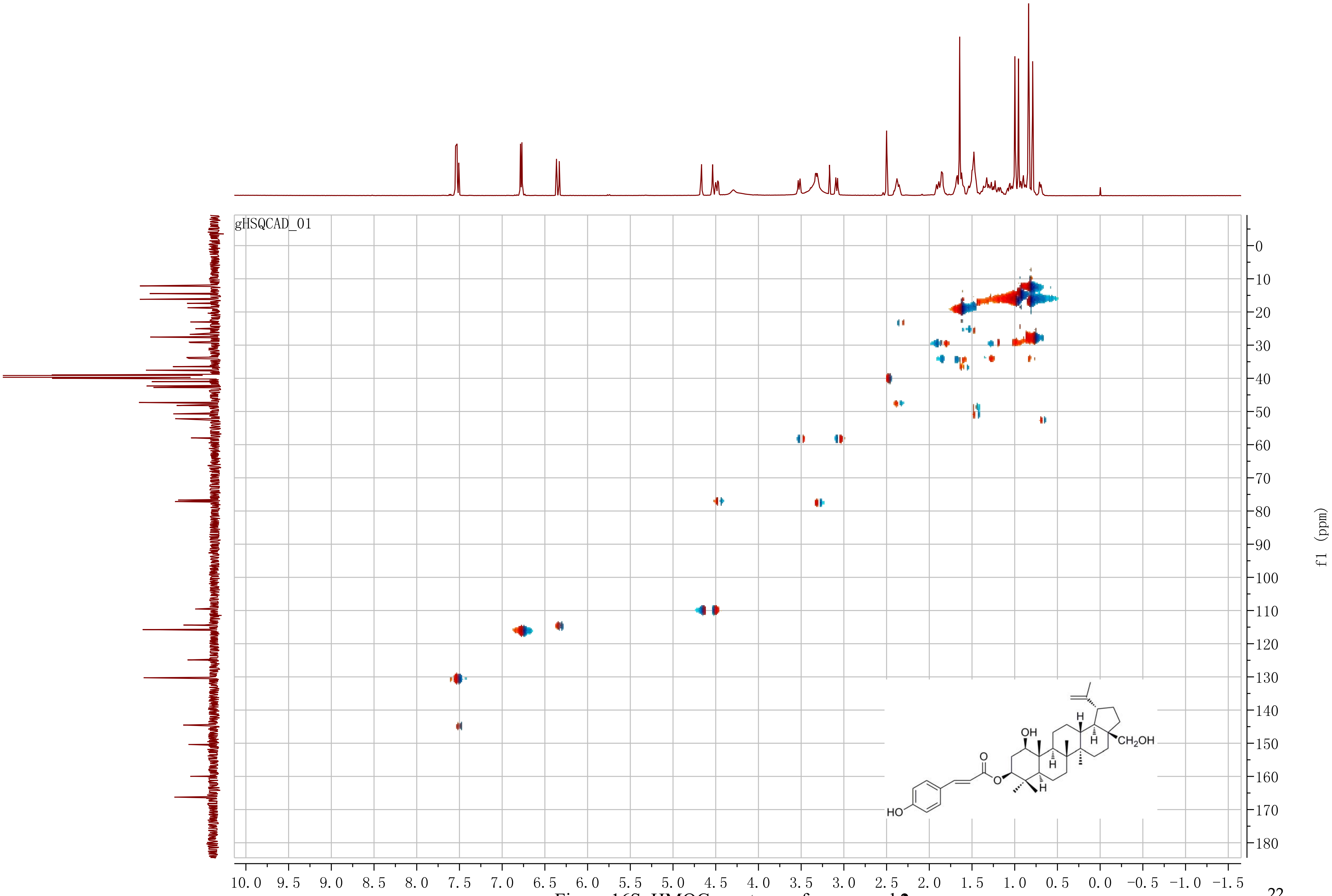

Figure 16S. HMQC spectrum of compound **2**

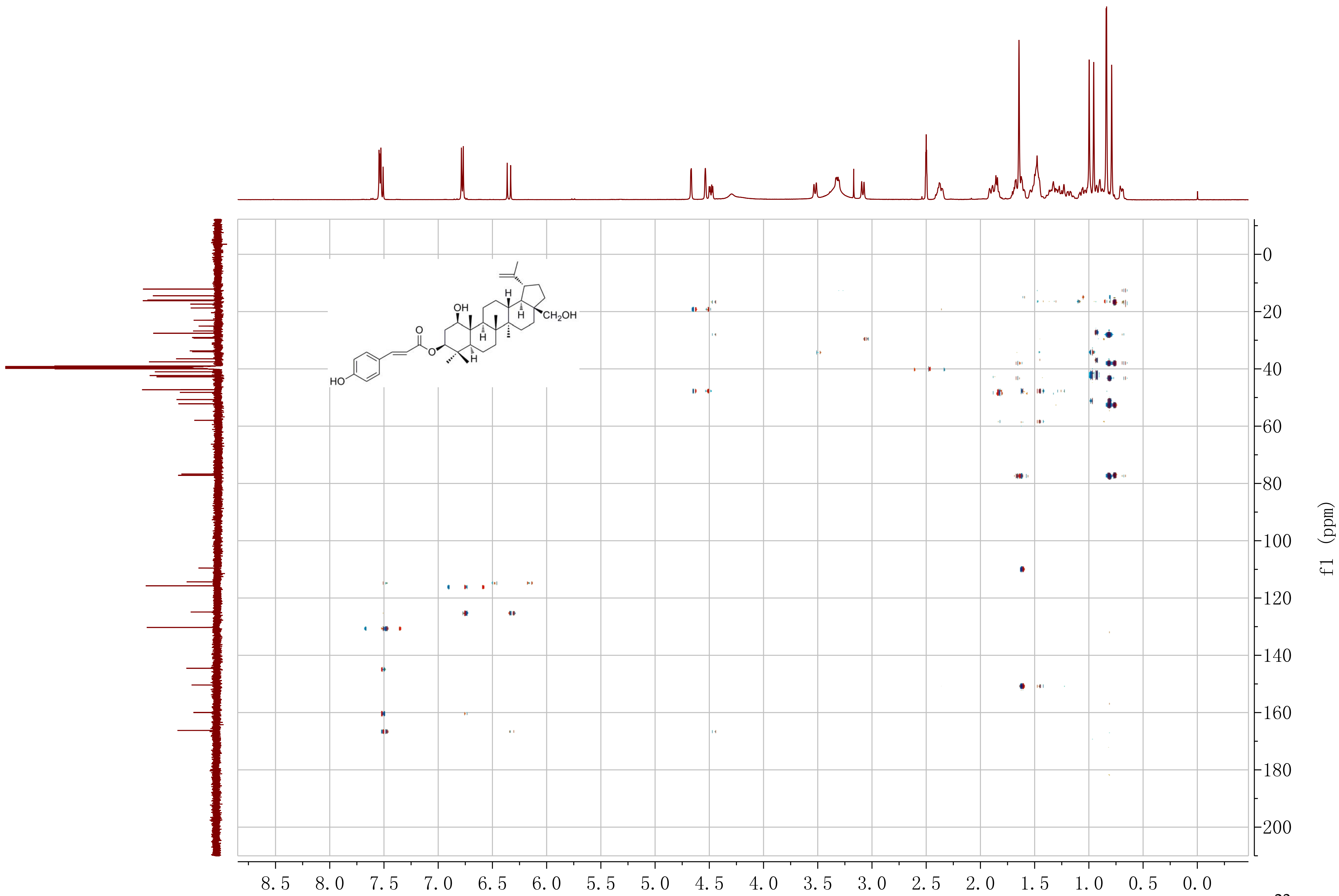

Figure 17S. HMBC spectrum of compound **2**

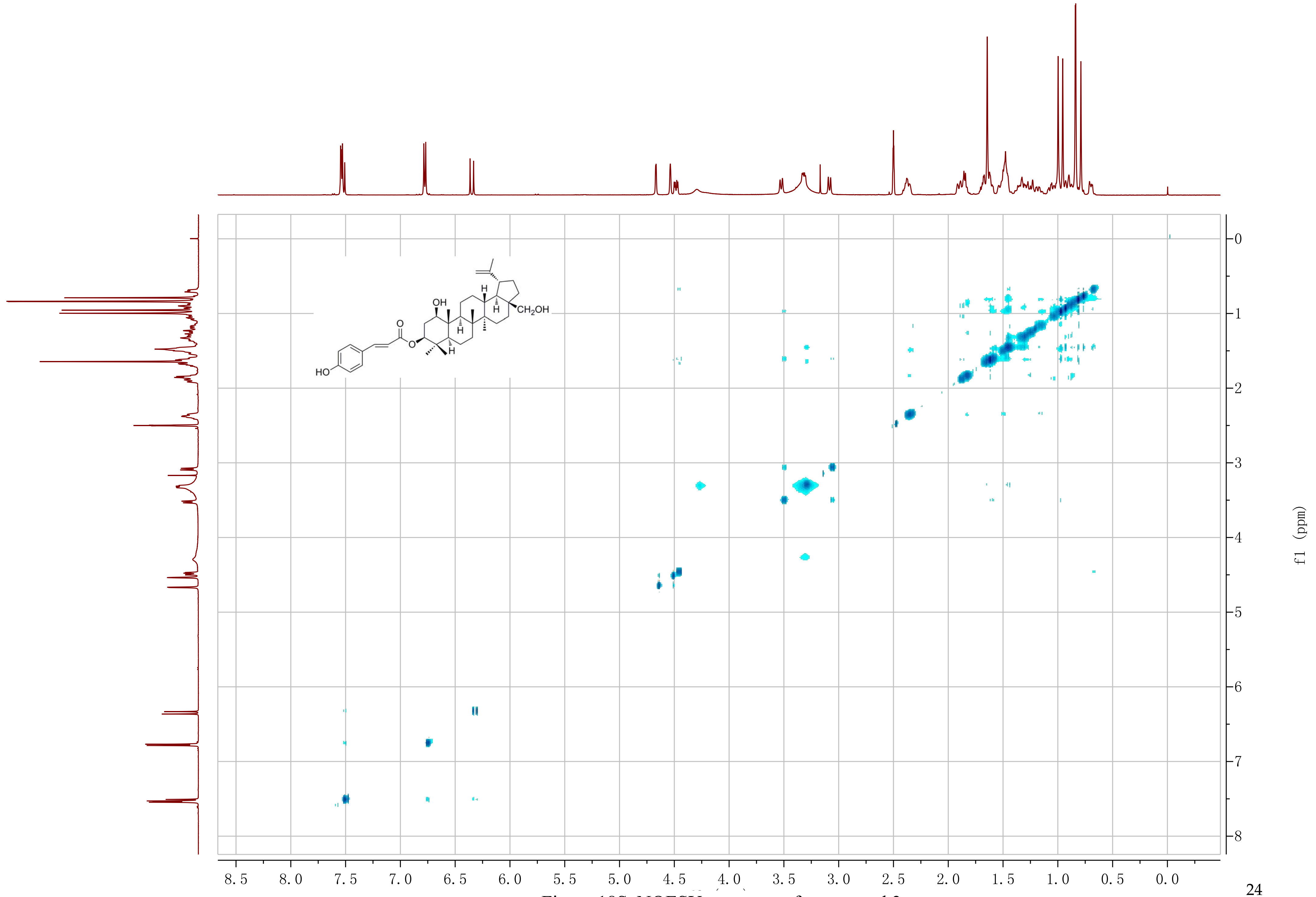

Figure 18S. NOESY spectrum of compound **2**

20130827-9-4-3-4-2_130826150116 8/27/2013 10:03:43 AM 9-4-3-4-2

20130827-9-4-3-4-2_130826150116 #35-36 RT: 0.32-0.33 AV: 2 NL: 2.30E6
T: FTMS - p ESI Full ms [100.00-1000.00]

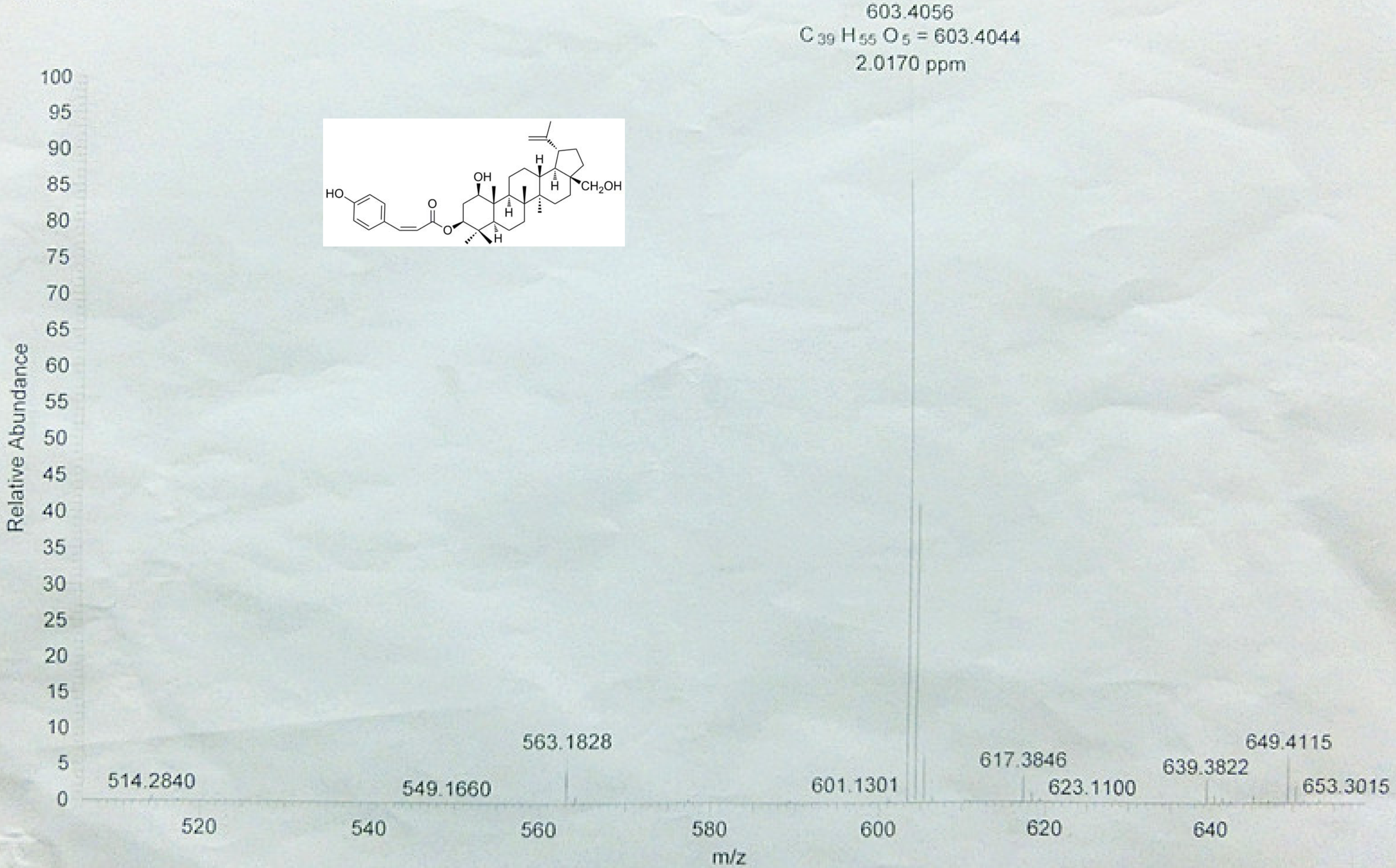

Figure 19S. The negtive HRESIMS spectrum of compound 3

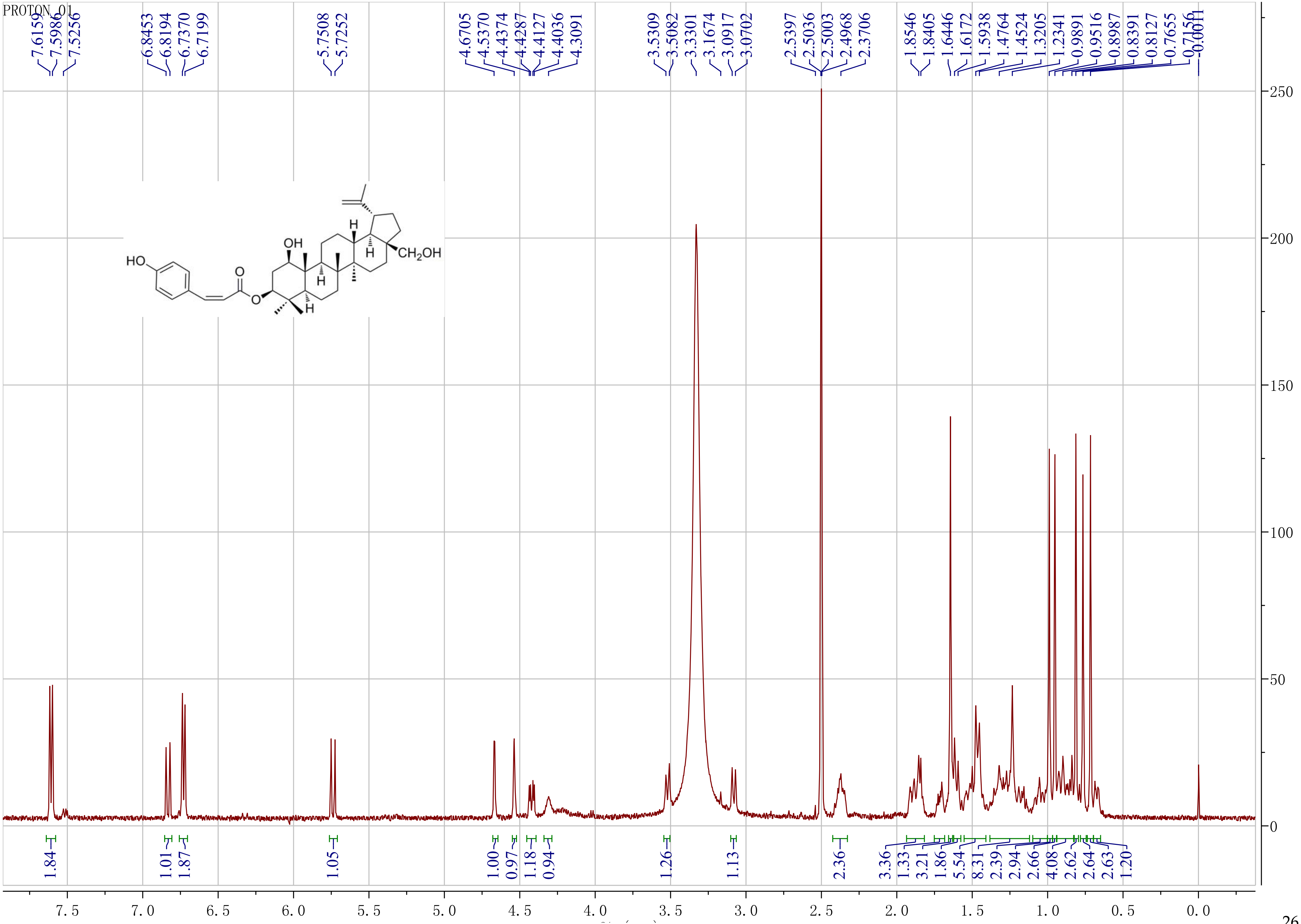

Figure 20S. ^{1}H-NMR (500M, DMSO) spectrum of compound **3**

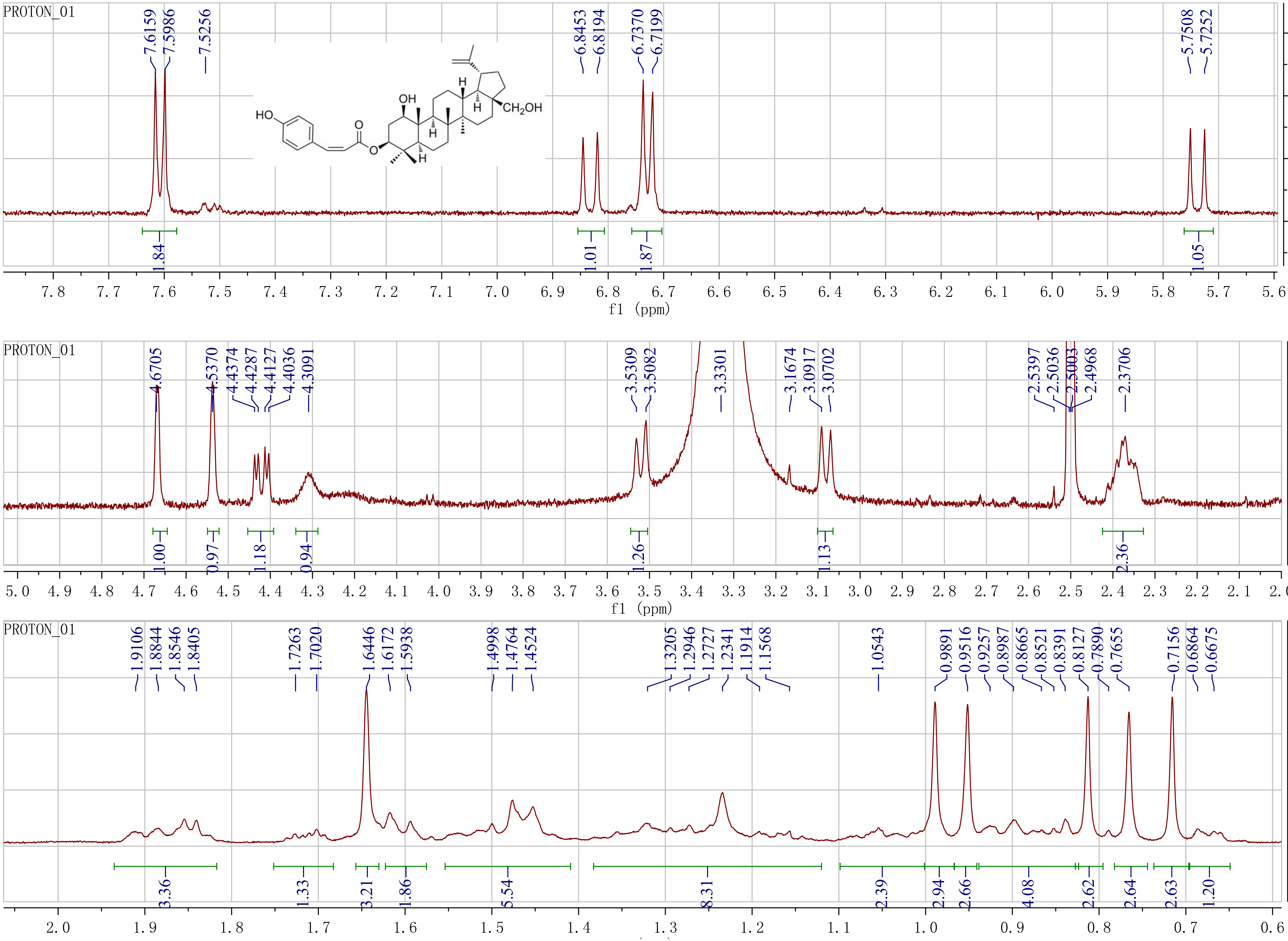

Figure 21S. The amplificatory ^{1}H-NMR (500M, DMSO) spectrum of compound **3**

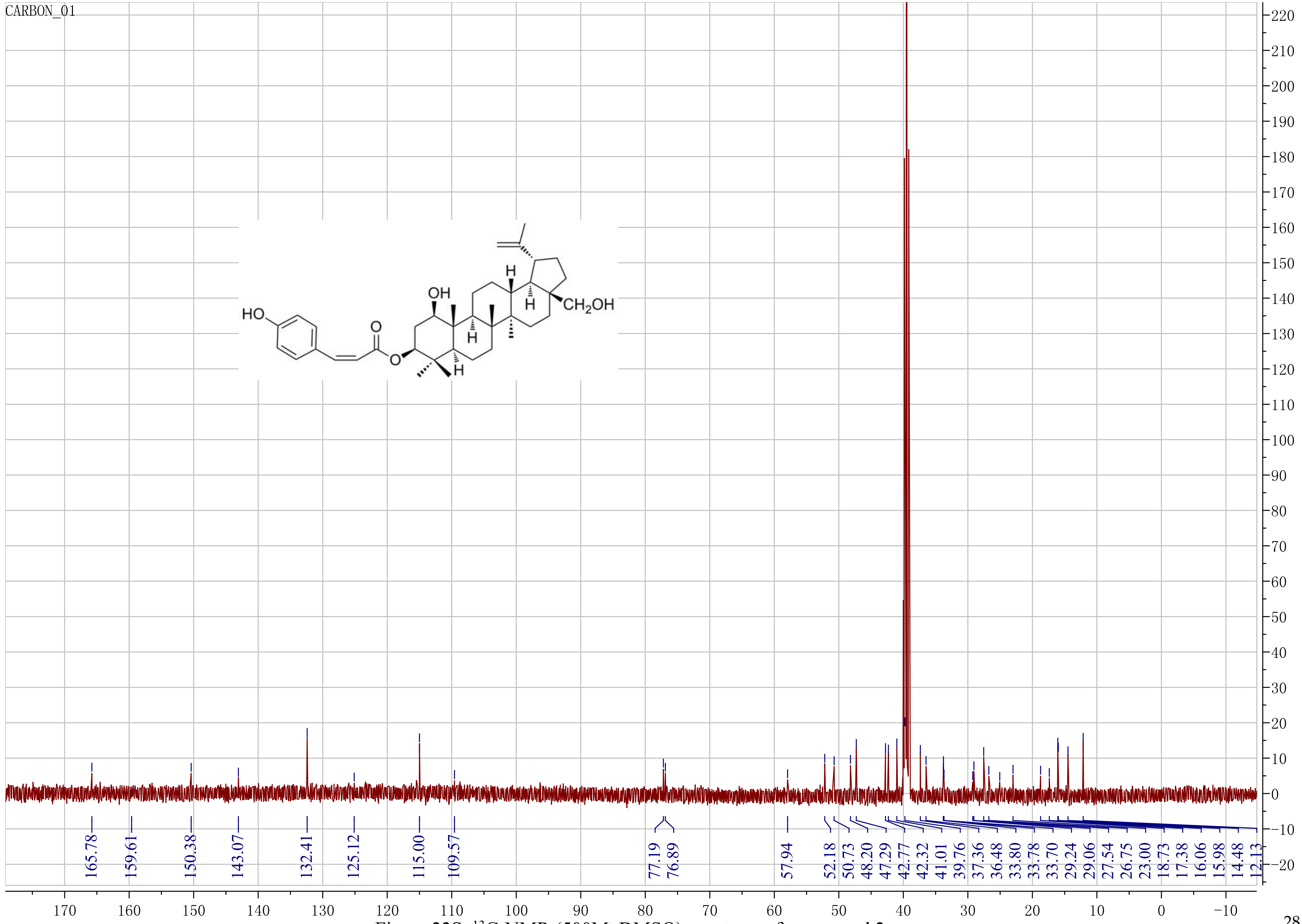

Figure 22S. ^{13}C-NMR (500M, DMSO) spectrum of compound **3**

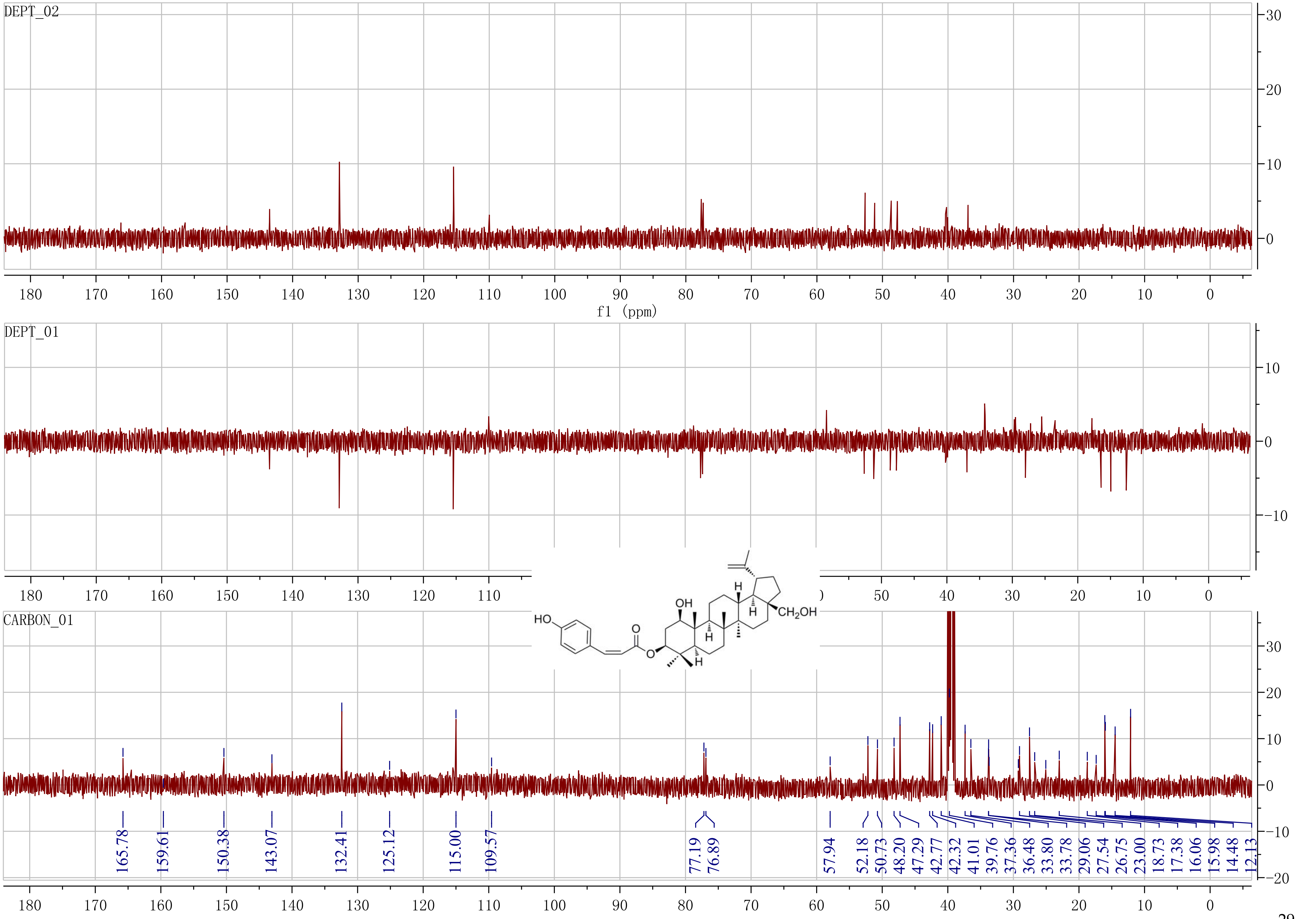

Figure 23S. DEPT (500M, DMSO) spectrum of compound **3**

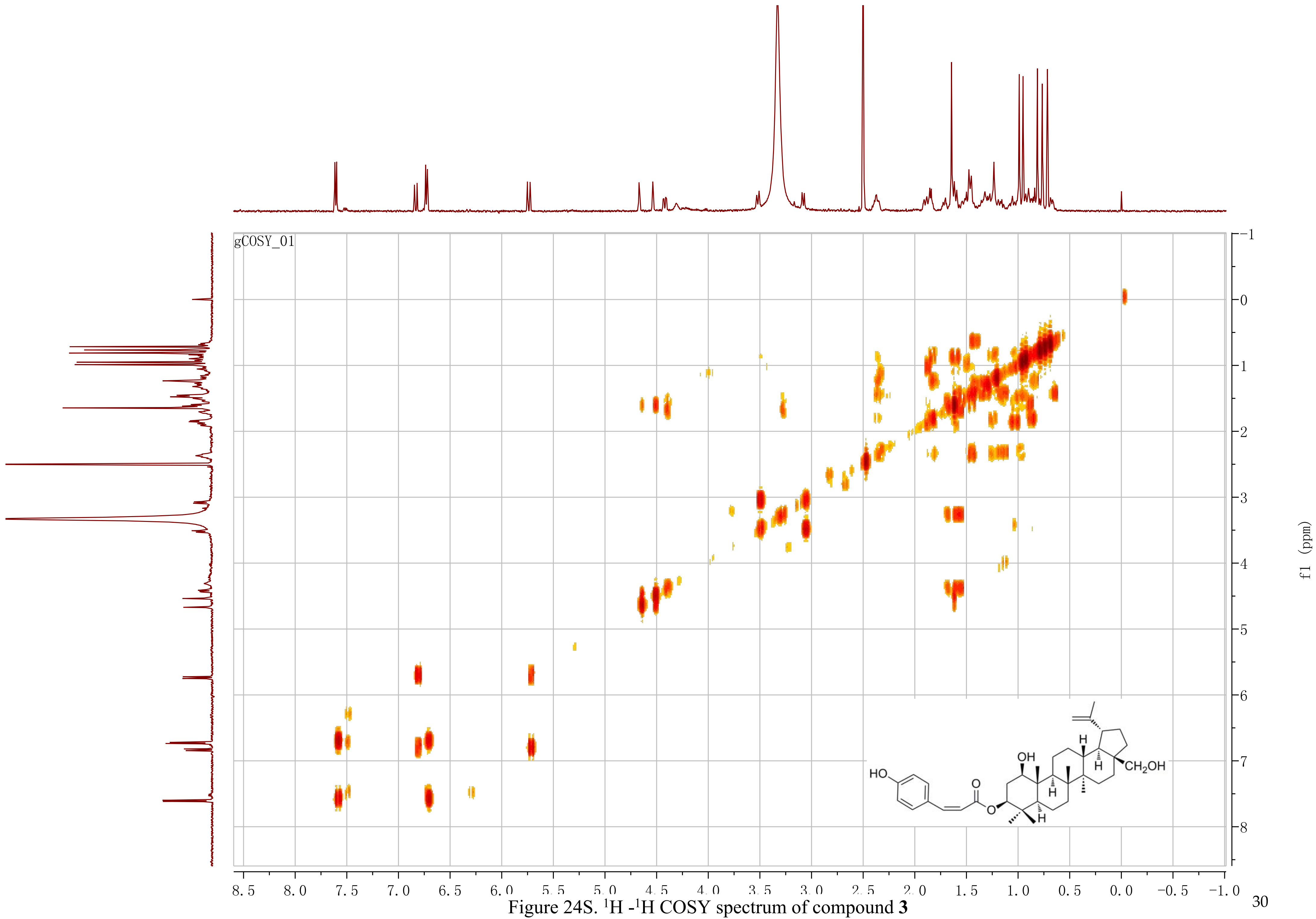

Figure 24S. ^{1}H -^{1}H COSY spectrum of compound **3**

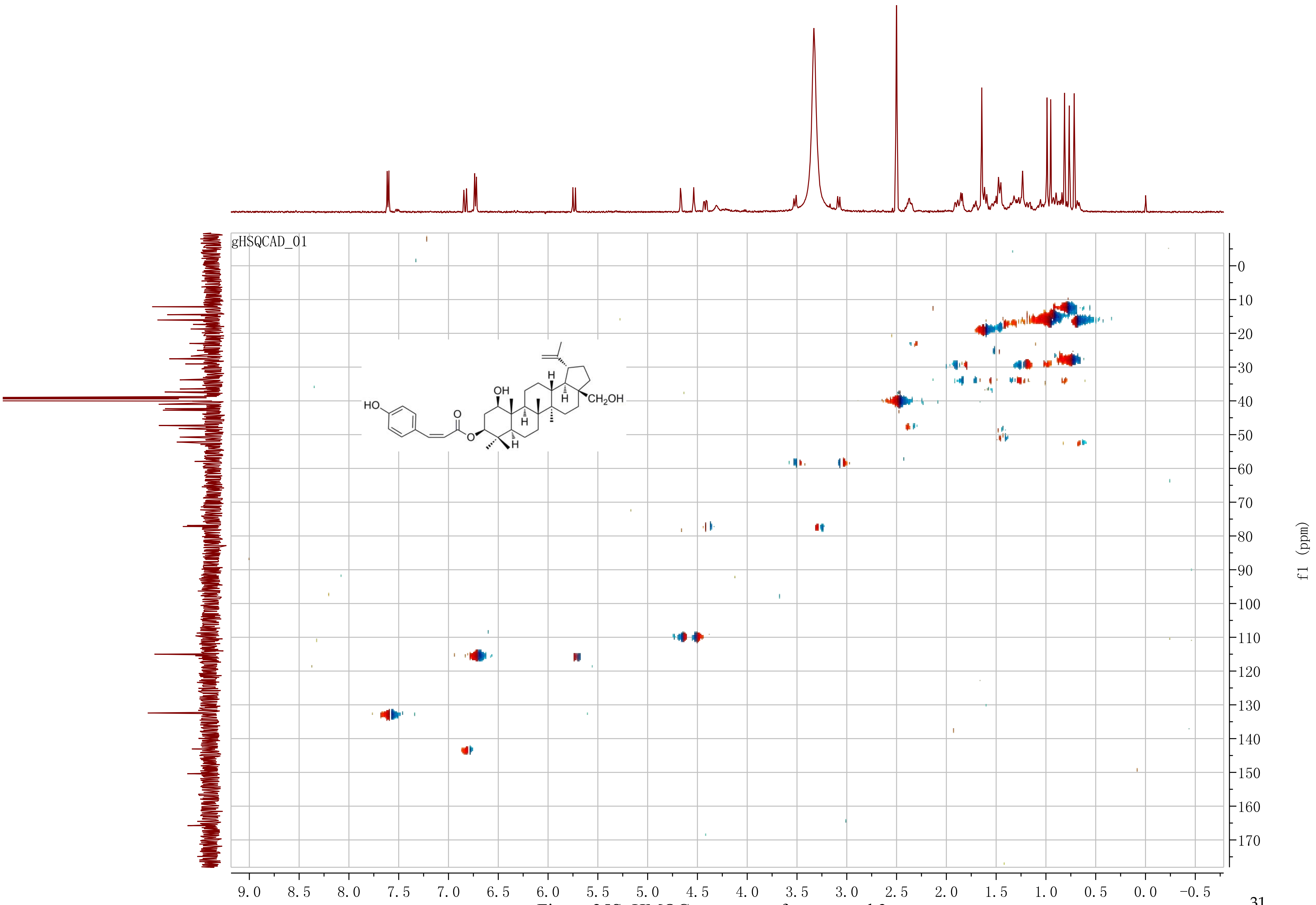

Figure 25S. HMQC spectrum of compound **3**

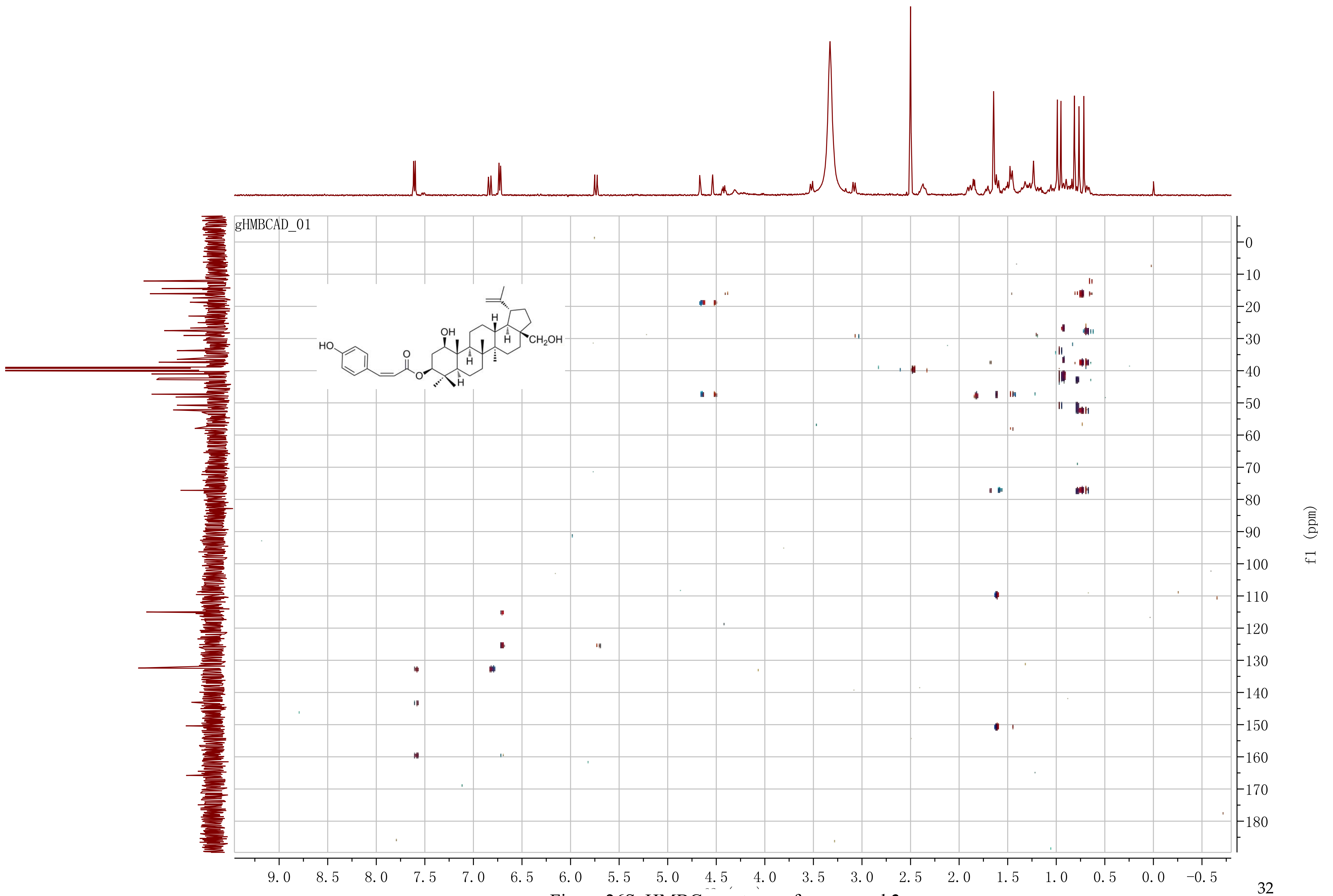

Figure 26S. HMBC spectrum of compound **3**

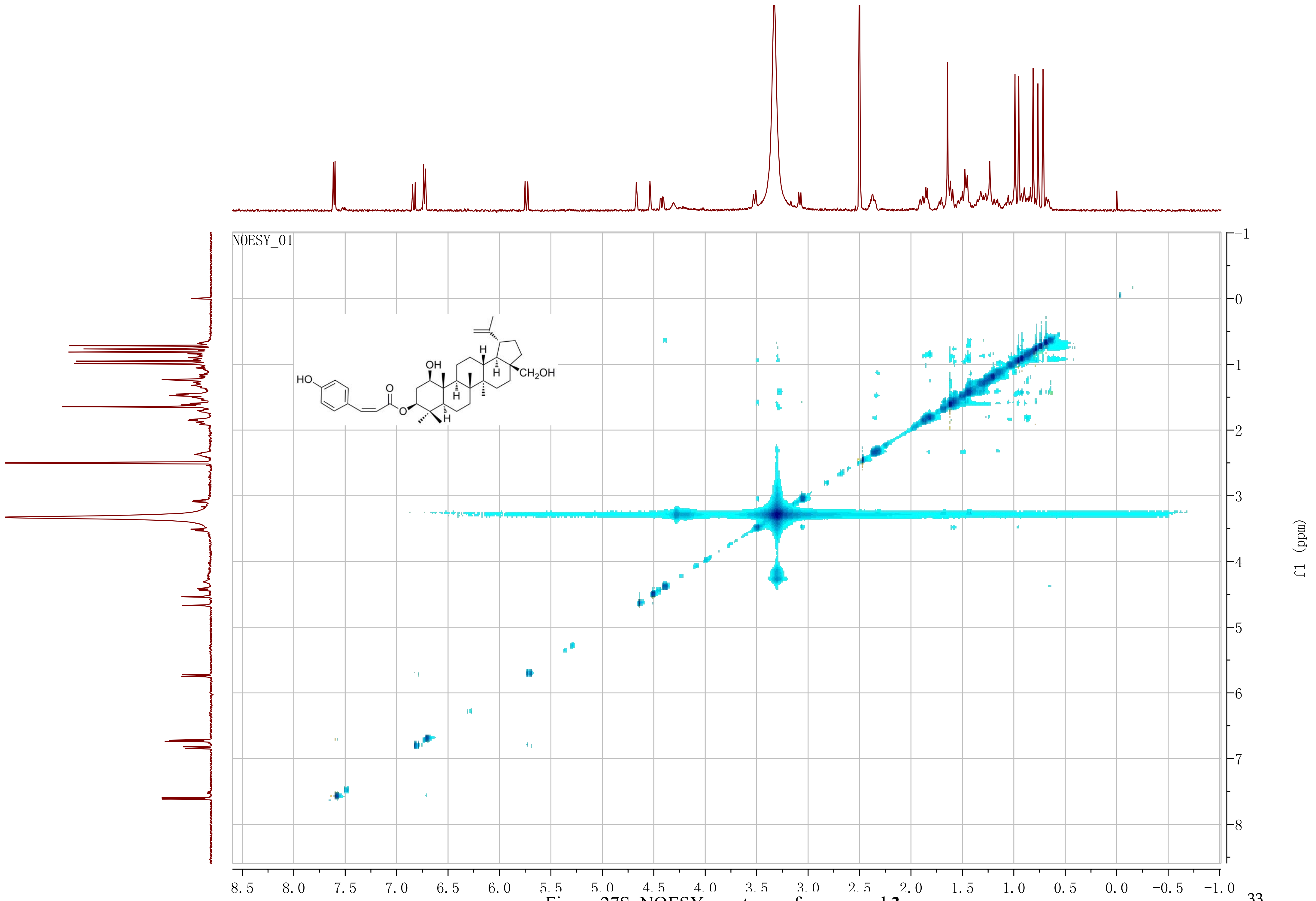

Figure 27S. NOESY spectrum of compound **3**

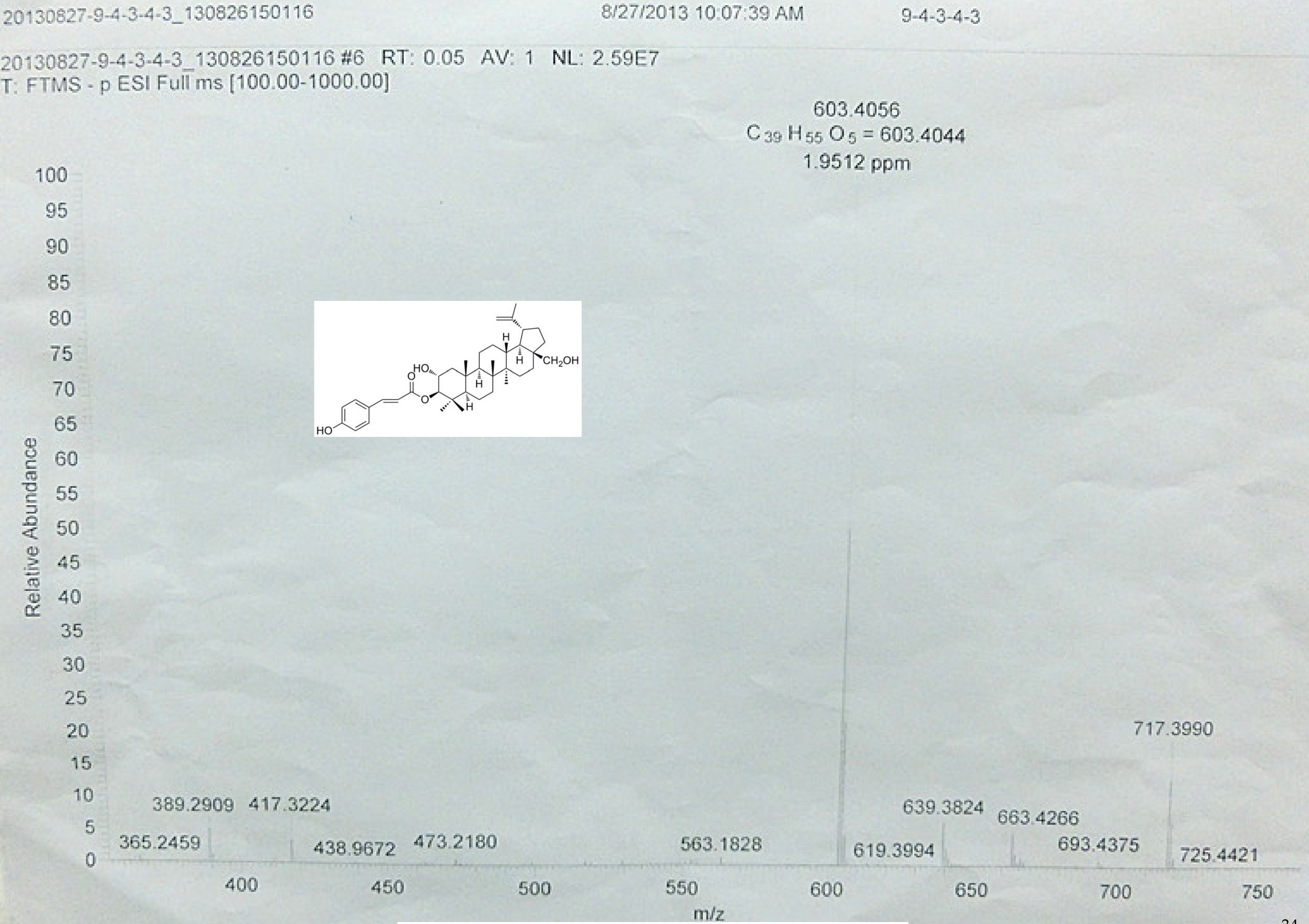

Figure 28S. The negtive HRESIMS spectrum of compound 4

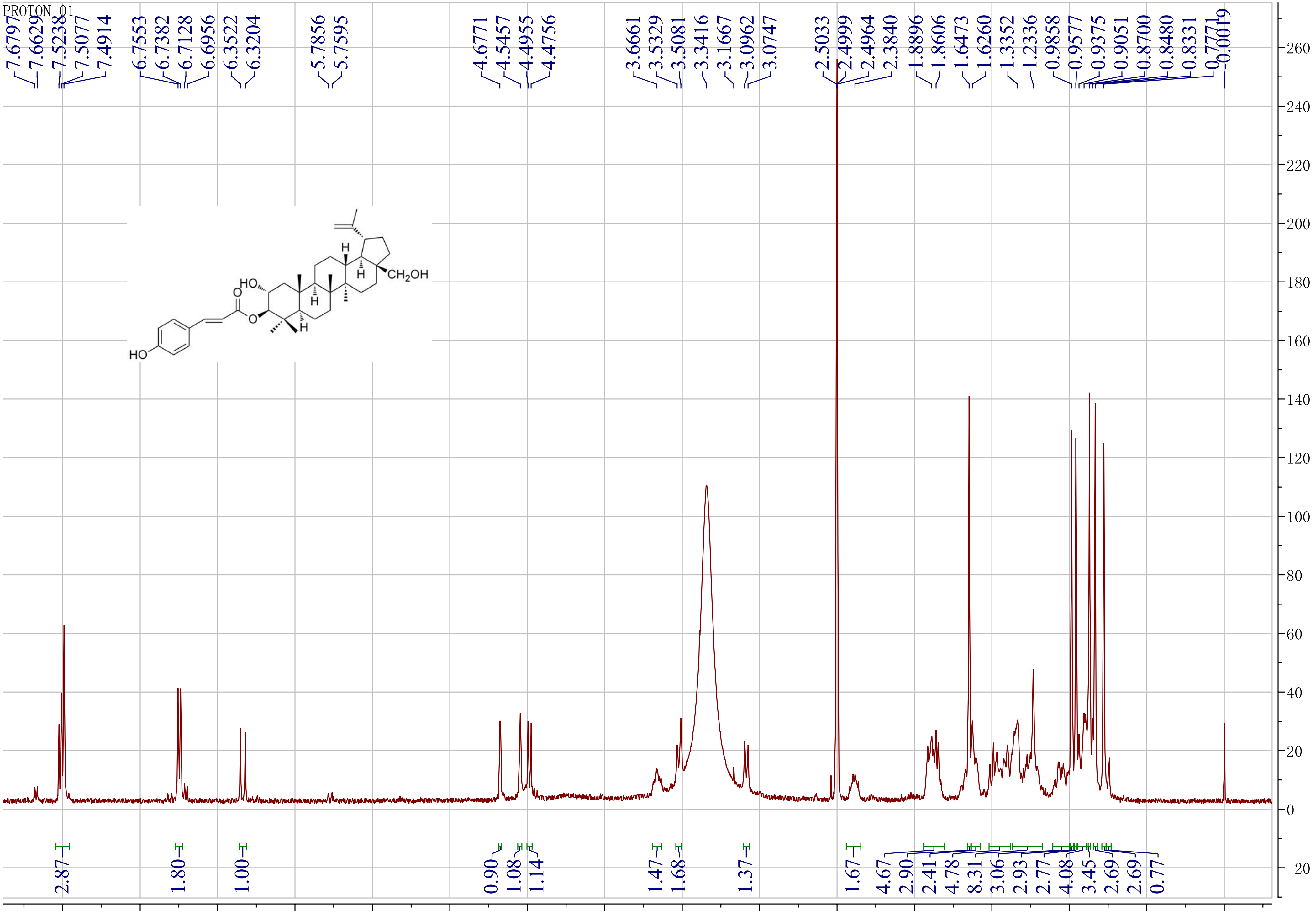

Figure 29S. ^{1}H-NMR (500M, DMSO) spectrum of compound **4**

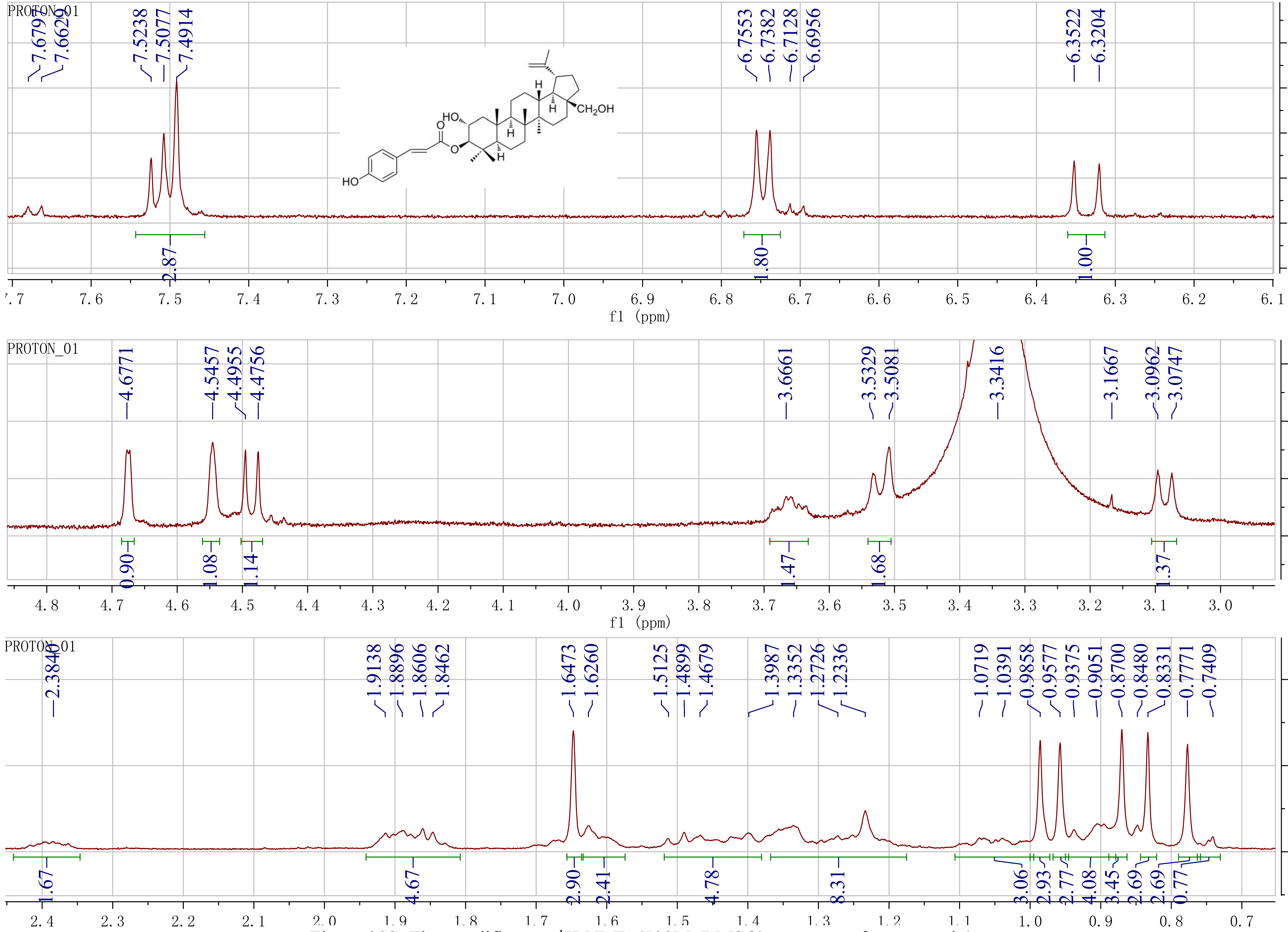

Figure 30S. The amplificatory ^{1}H-NMR (500M, DMSO) spectrum of compound **4**

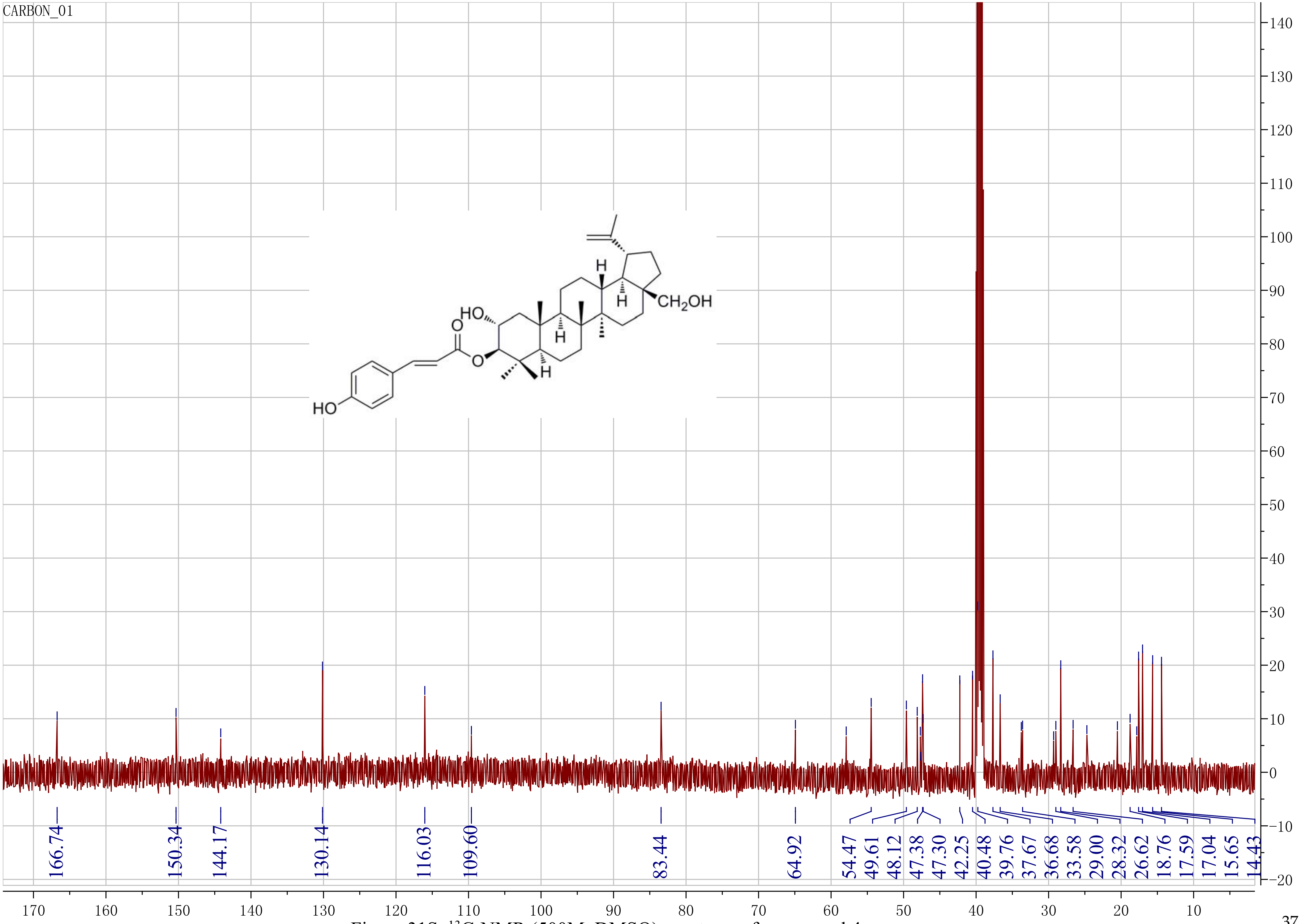

Figure 31S. ^{13}C-NMR (500M, DMSO) spectrum of compound **4**

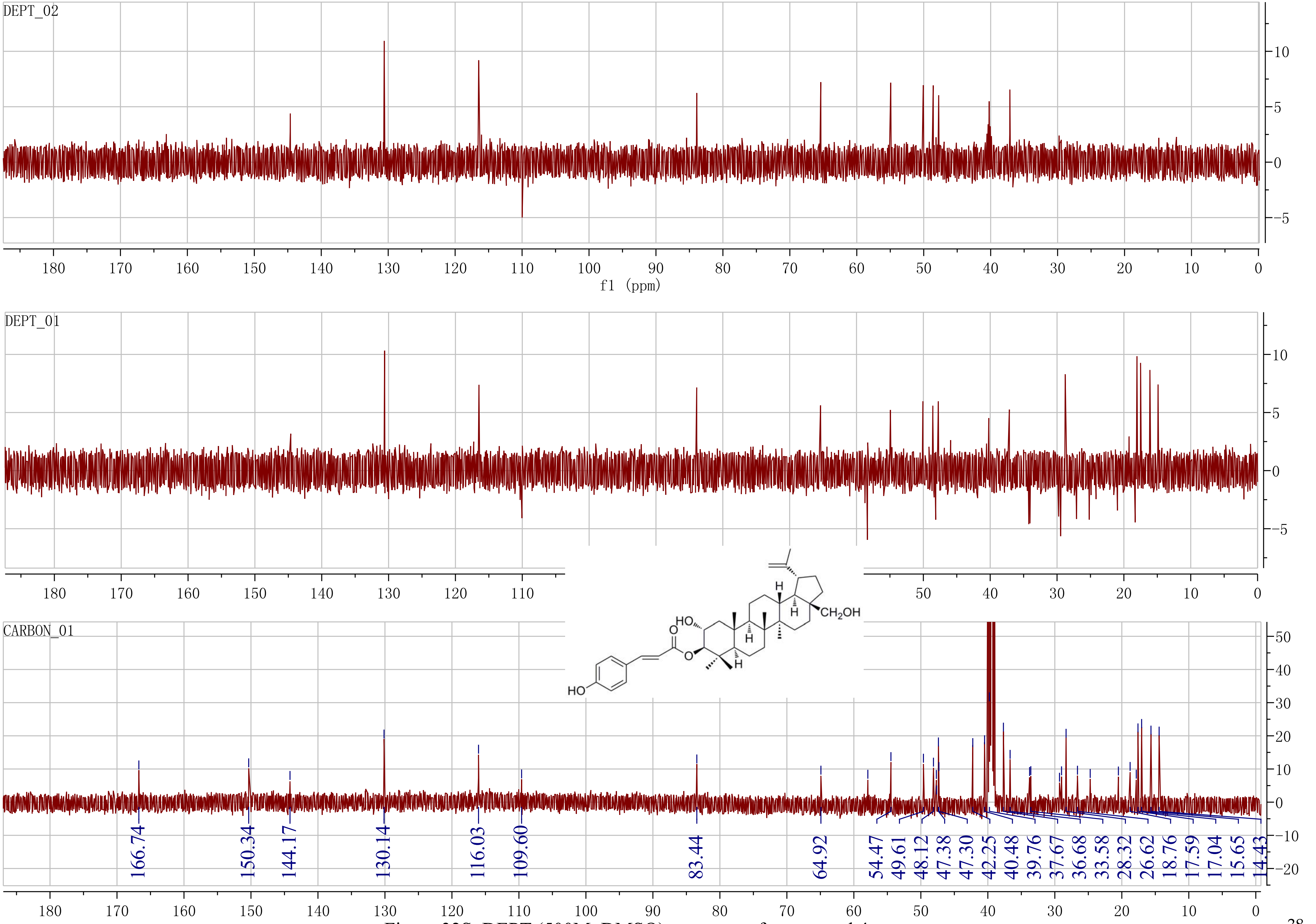

Figure 32S. DEPT (500M, DMSO) spectrum of compound **4**

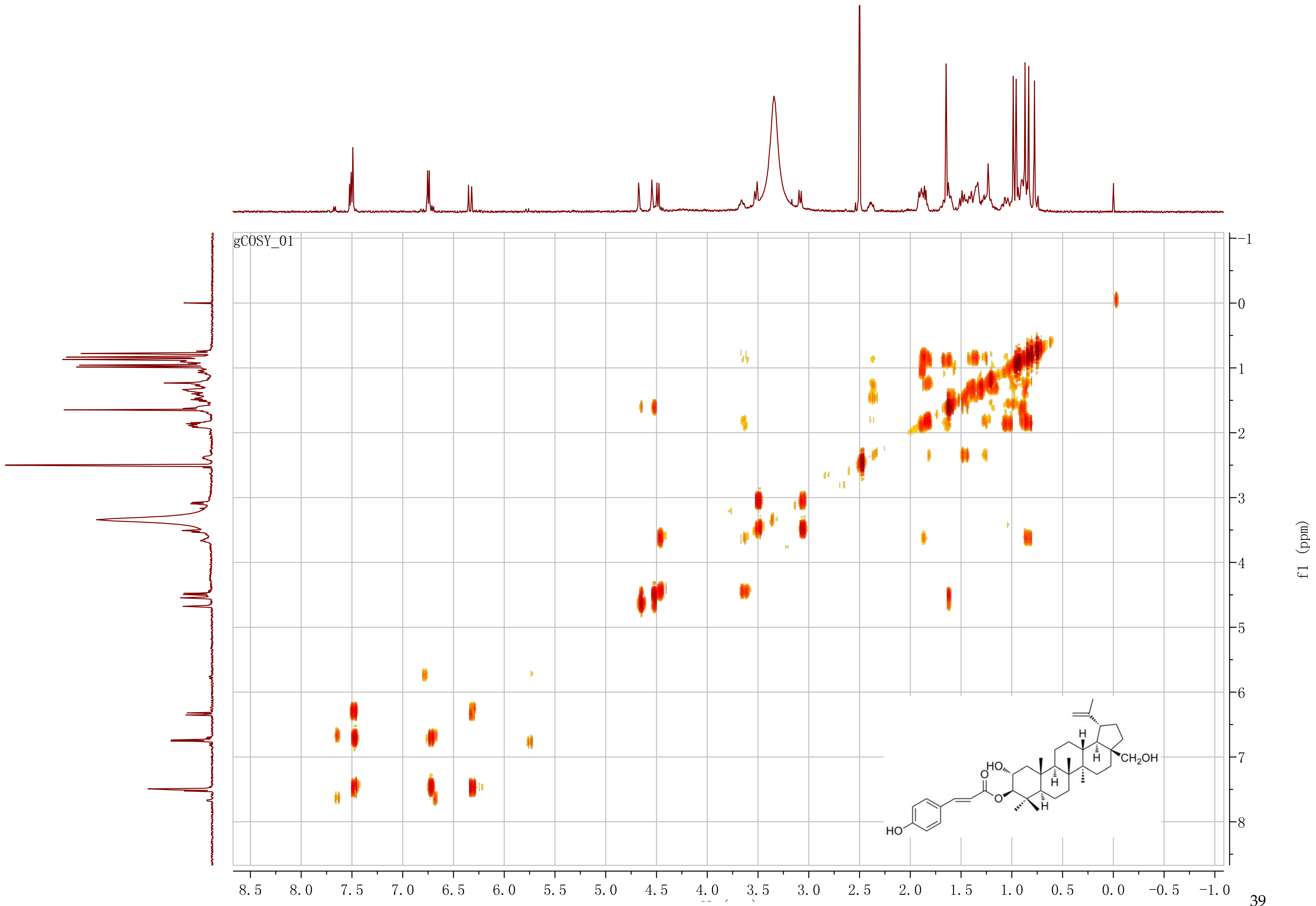

Figure 33S. ^{1}H -^{1}H COSY spectrum of compound **4**

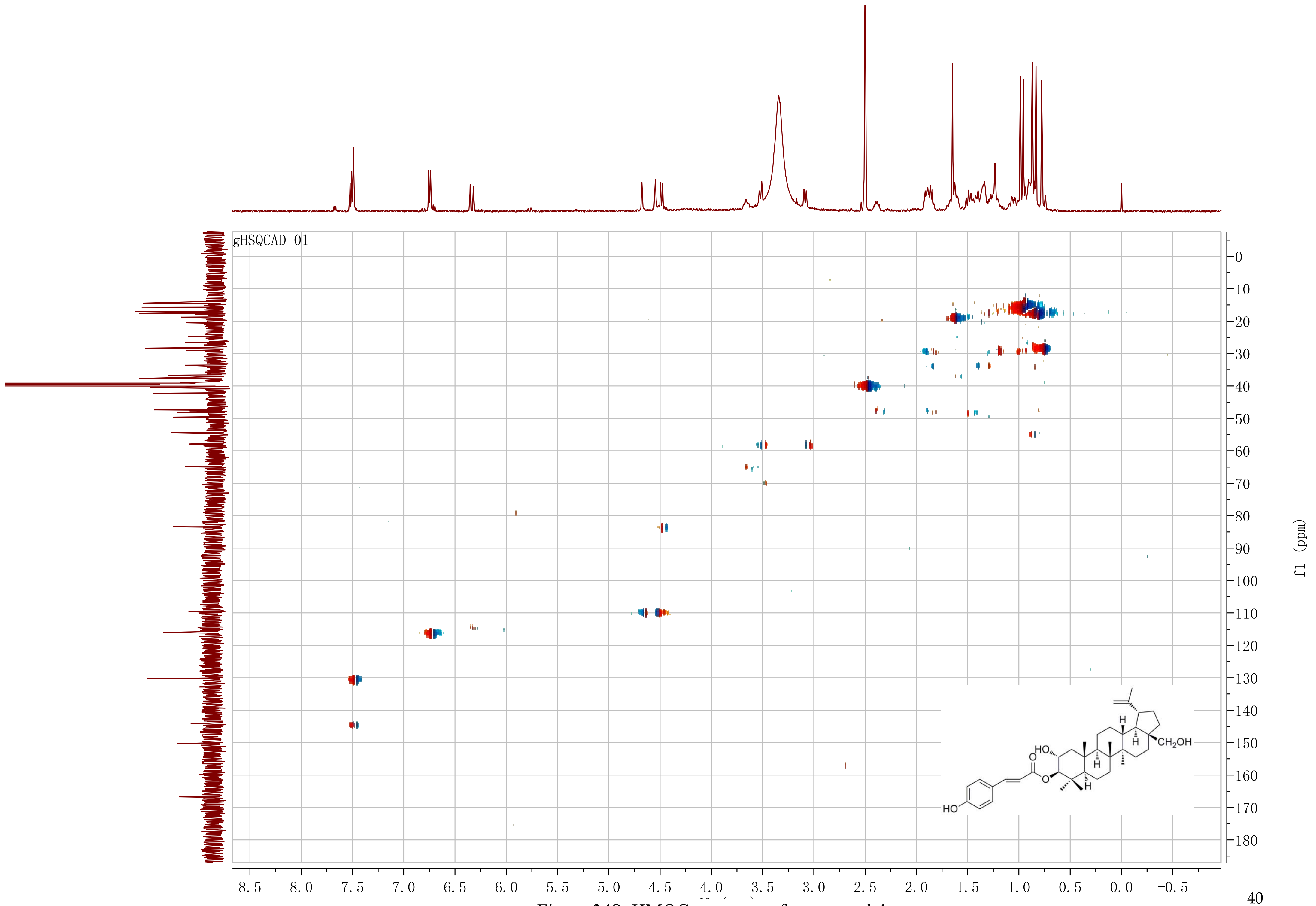

Figure 34S. HMQC spectrum of compound **4**

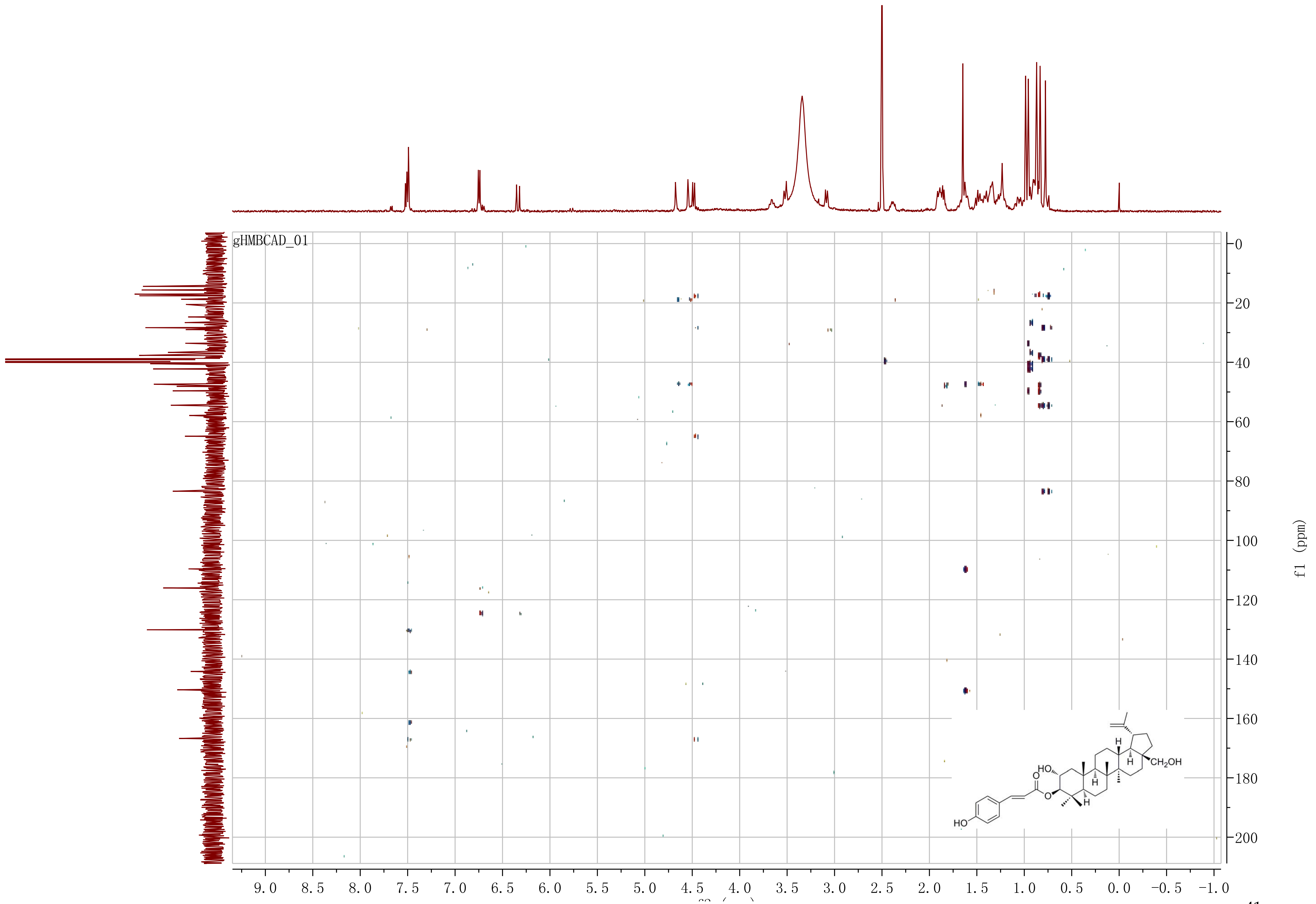

Figure 35S. HMBC spectrum of compound **4**

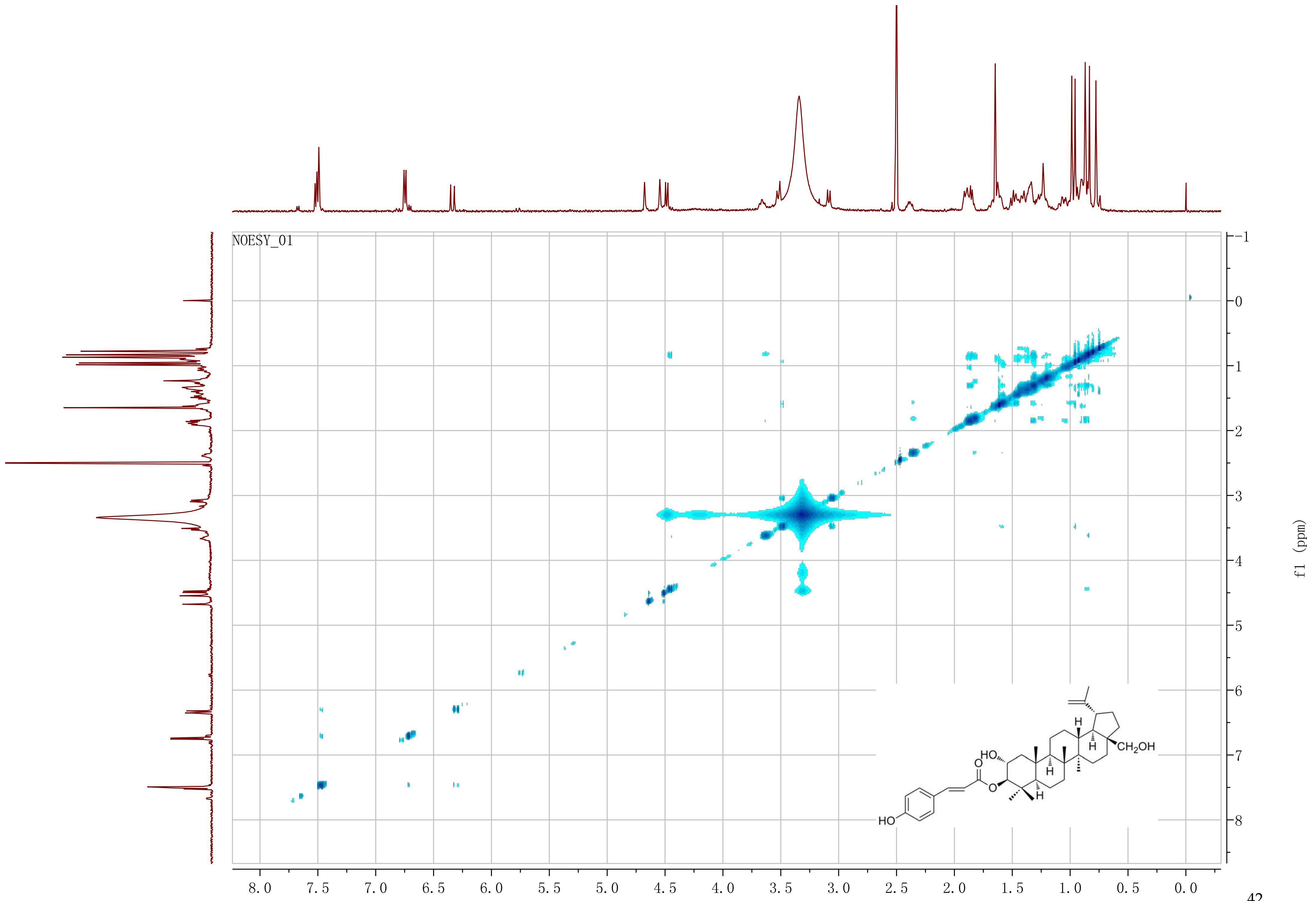

Figure 36S. NOESY spectrum of compound **4**

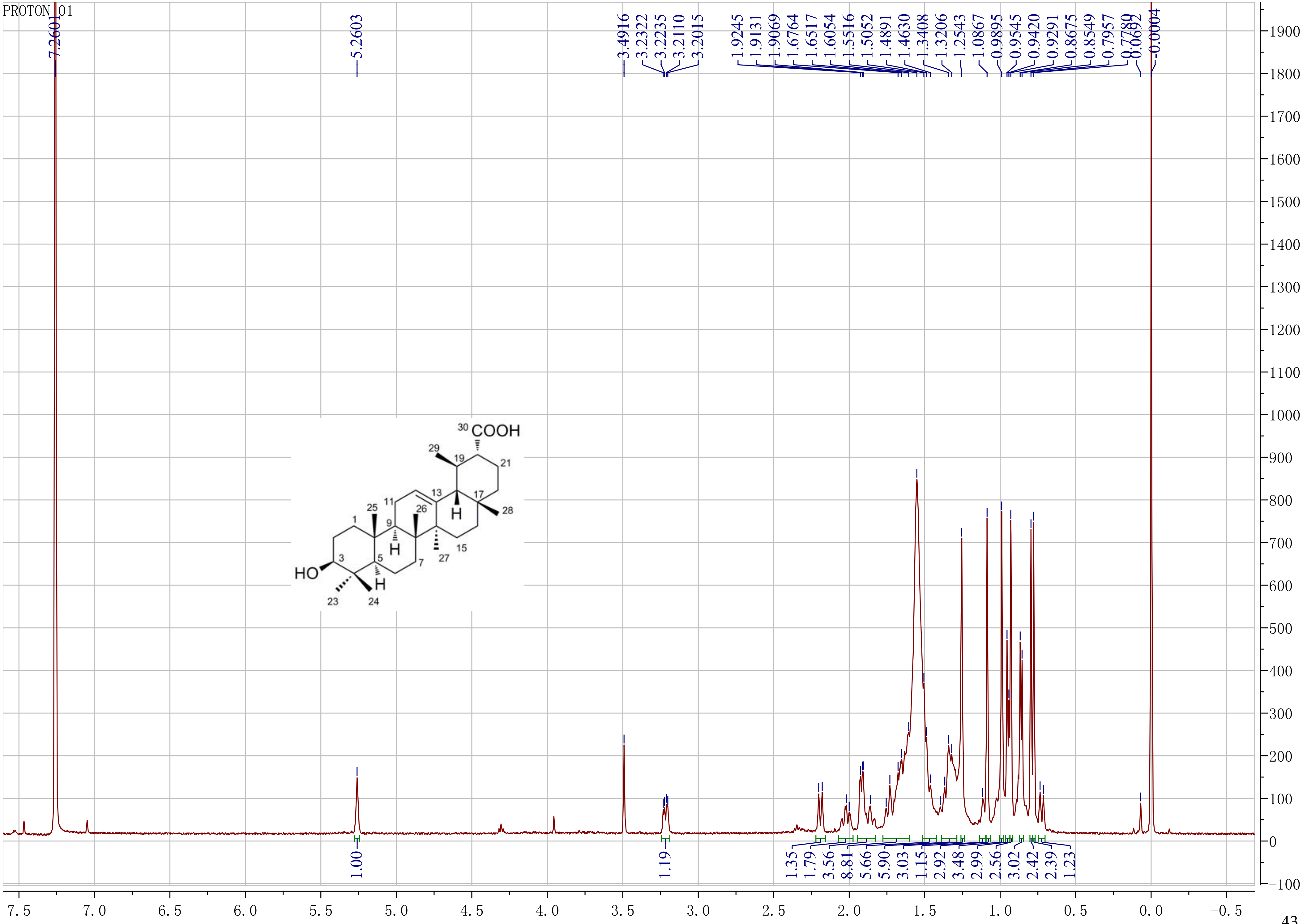

Figure 37S. ^{1}H-NMR (500M, $CDCl_3$) spectrum of compound **12**

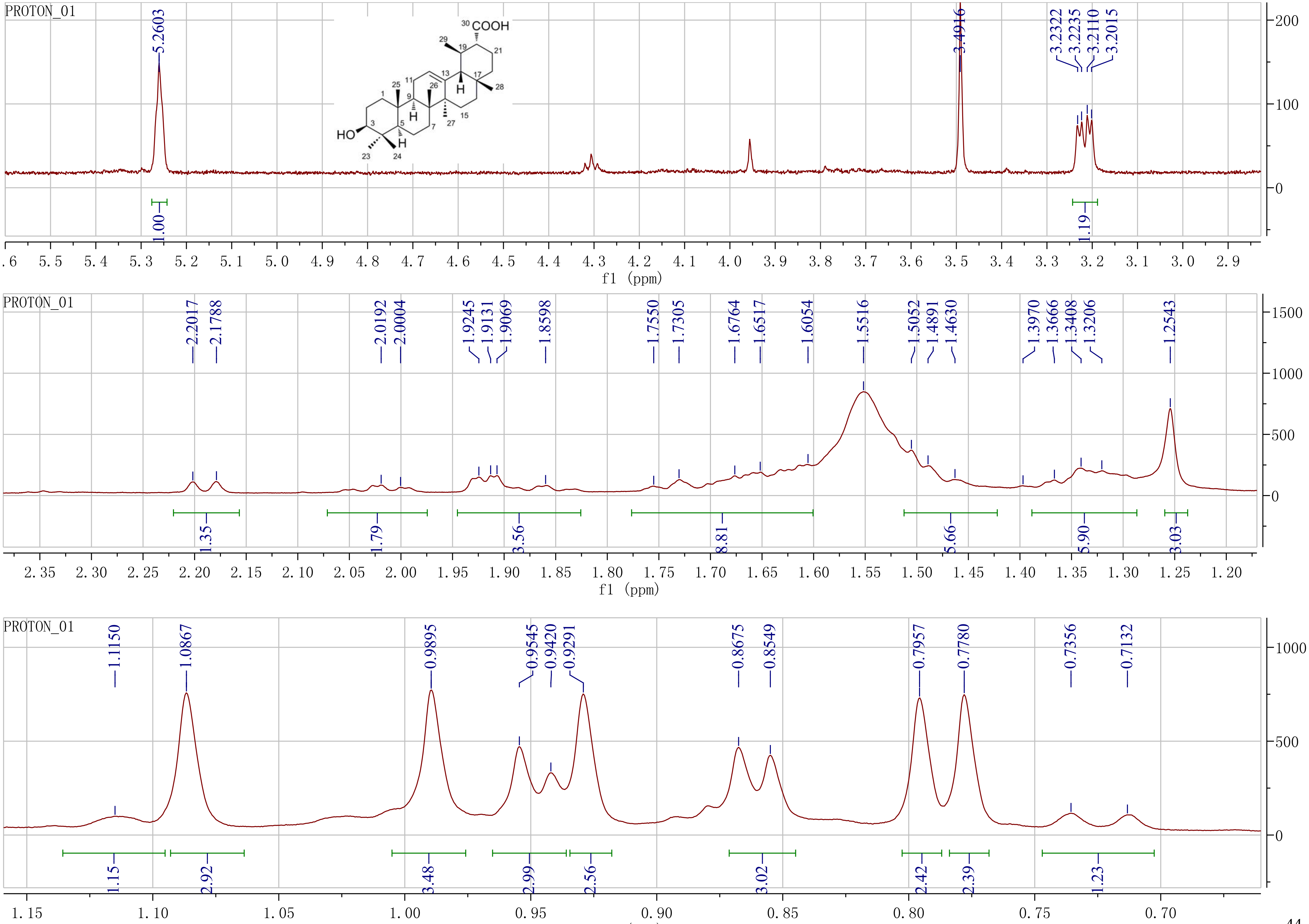

Figure 38S.The amplificatory ^{1}H NMR (500M, $CDCl_3$) spectrum of compound **12**

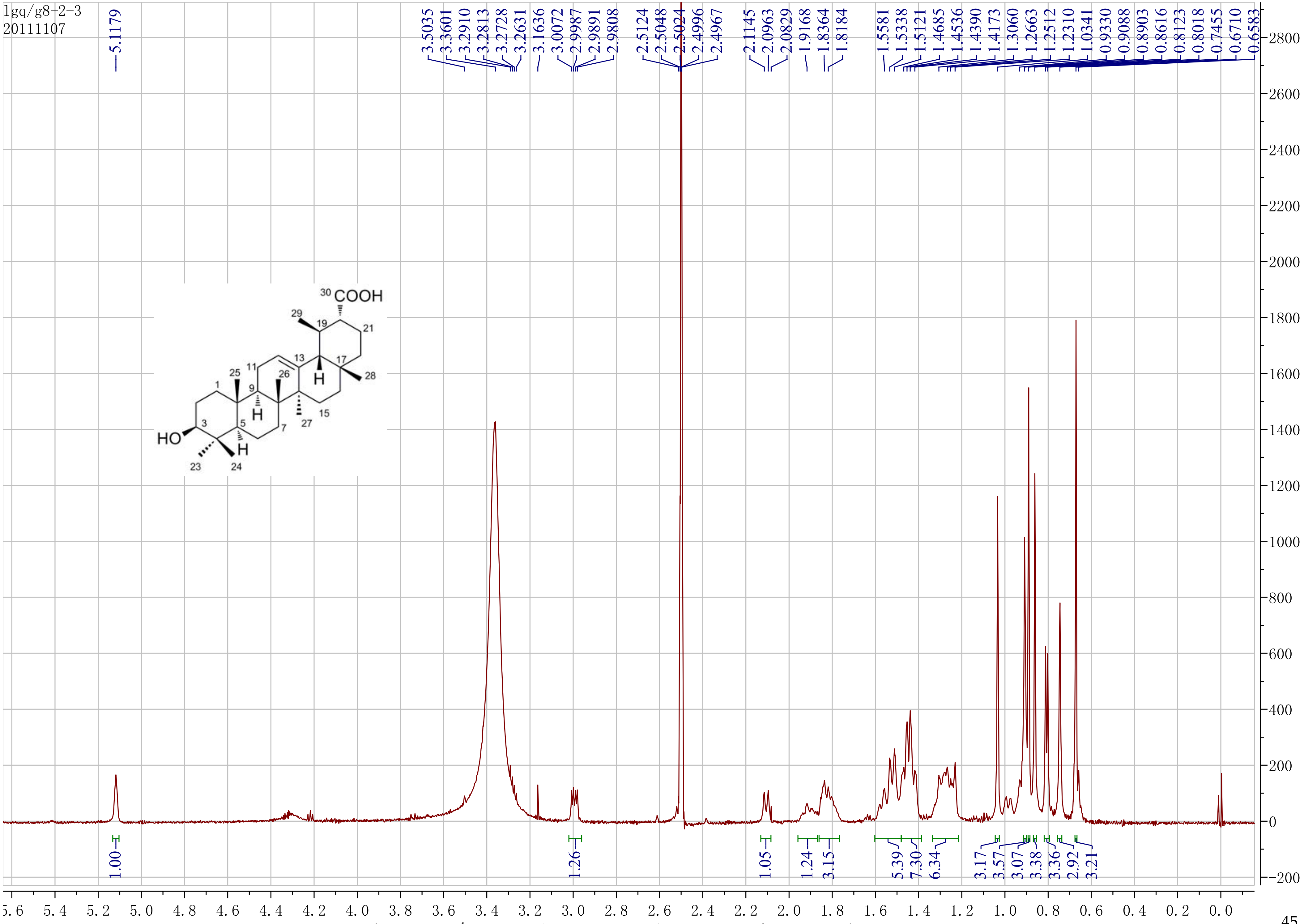

Figure 39S. ^{1}H-NMR (600M, DMSO) spectrum of compound **12**

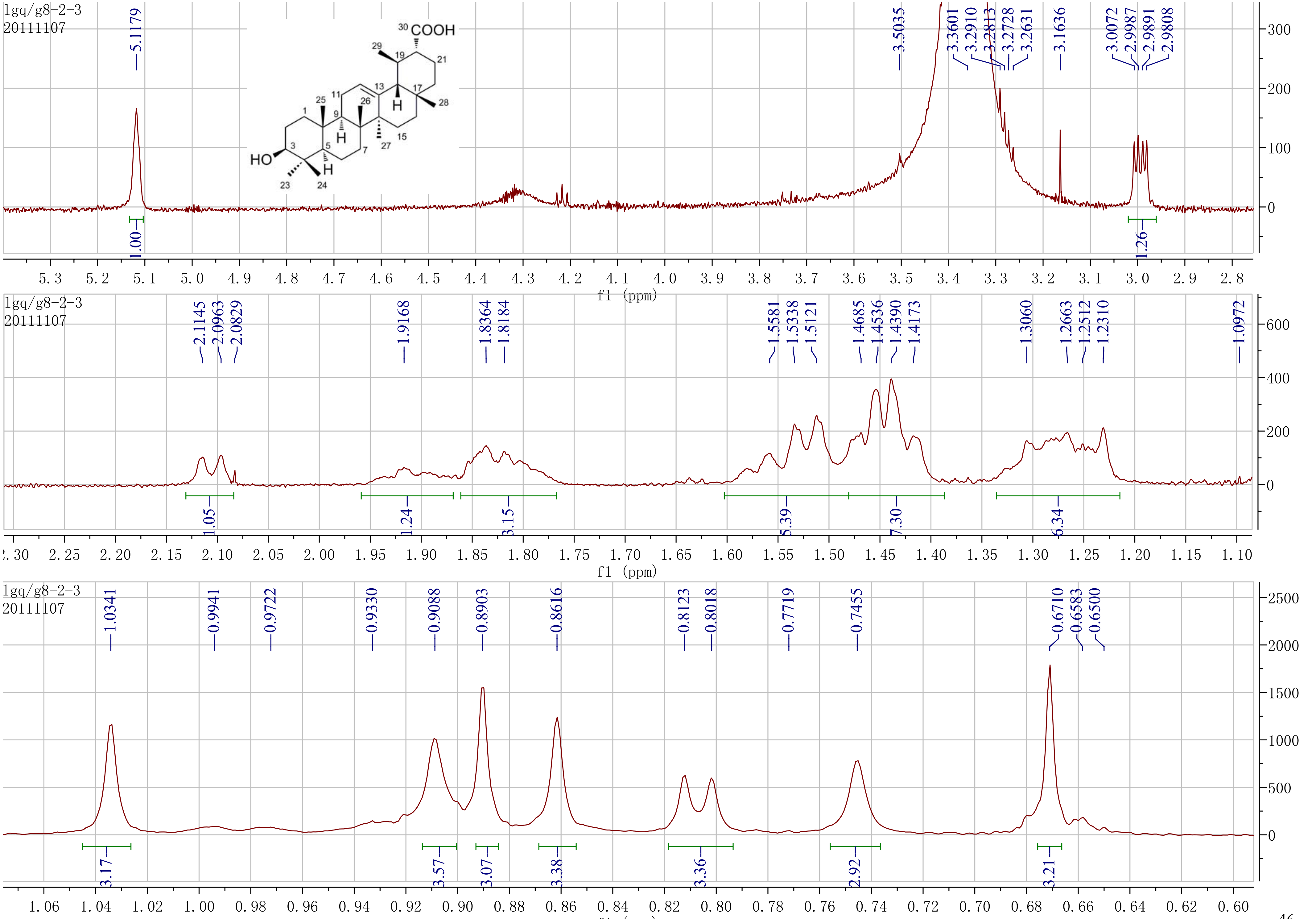

Figure 40S. The amplificatory ^{1}H NMR (600M, DMSO) spectrum of compound **12**

D:\MS-DATA\20120319-gq-1-3_120316133855 3/19/2012 11:29:53 AM gq-1-3

20120319-gq-1-3_120316133855 #201 RT: 1.88 AV: 1 NL: 1.64E6
T: FTMS - p ESI Full ms [100.00-1000.00]

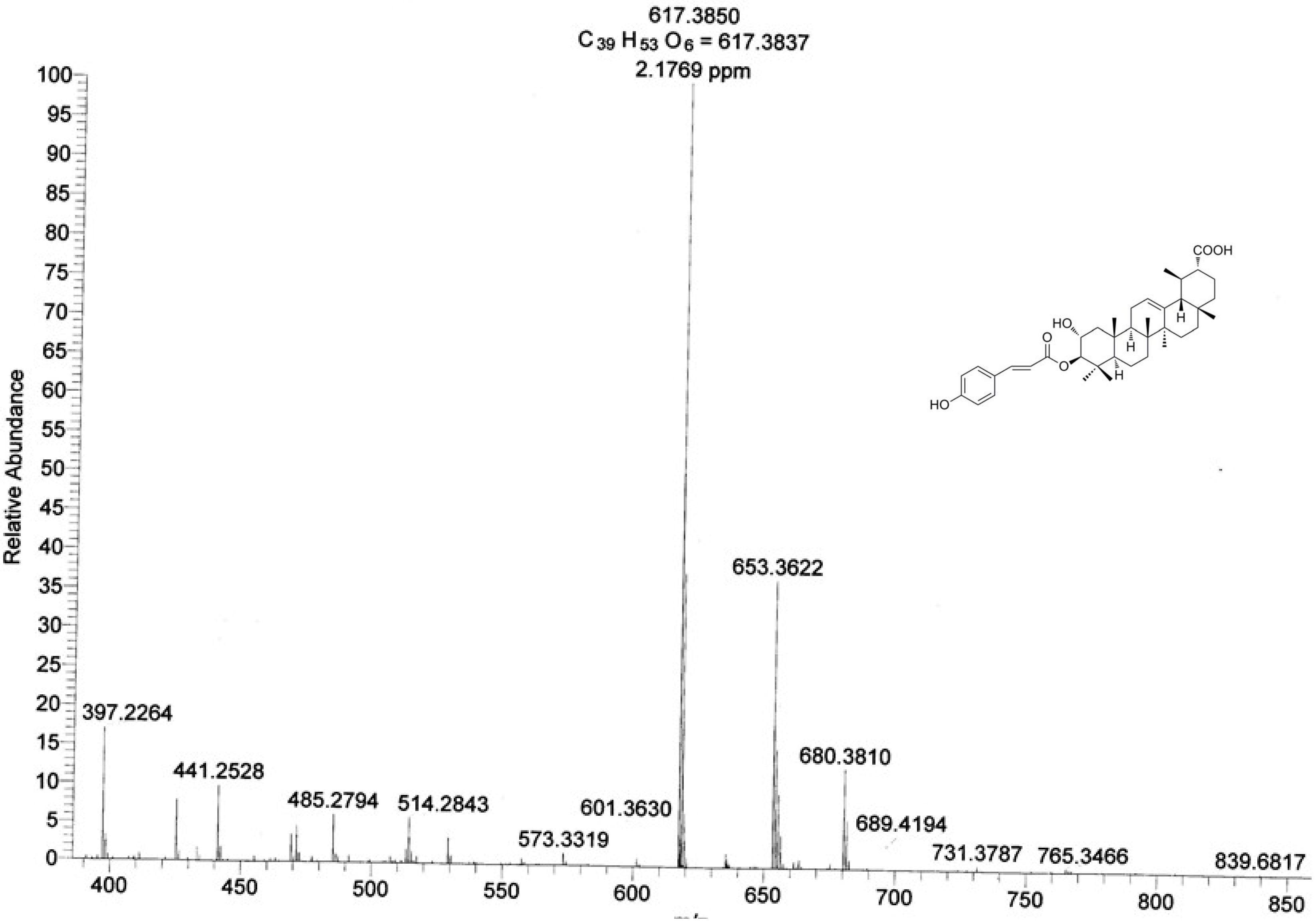

Figure 41S. The negtive HRESIMS spectrum of compound 11

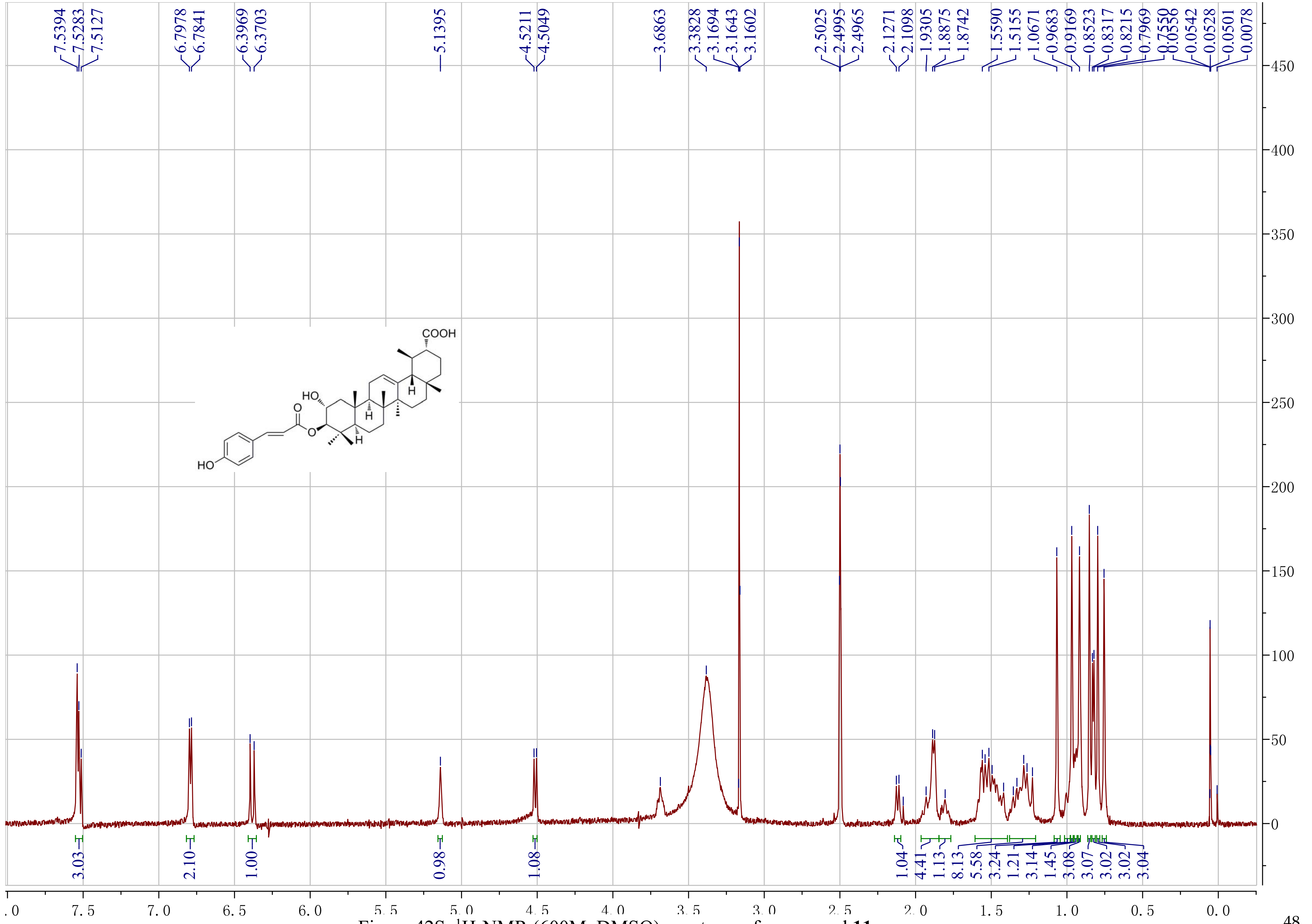

Figure 42S. ^{1}H-NMR (600M, DMSO) spectrum of compound **11**

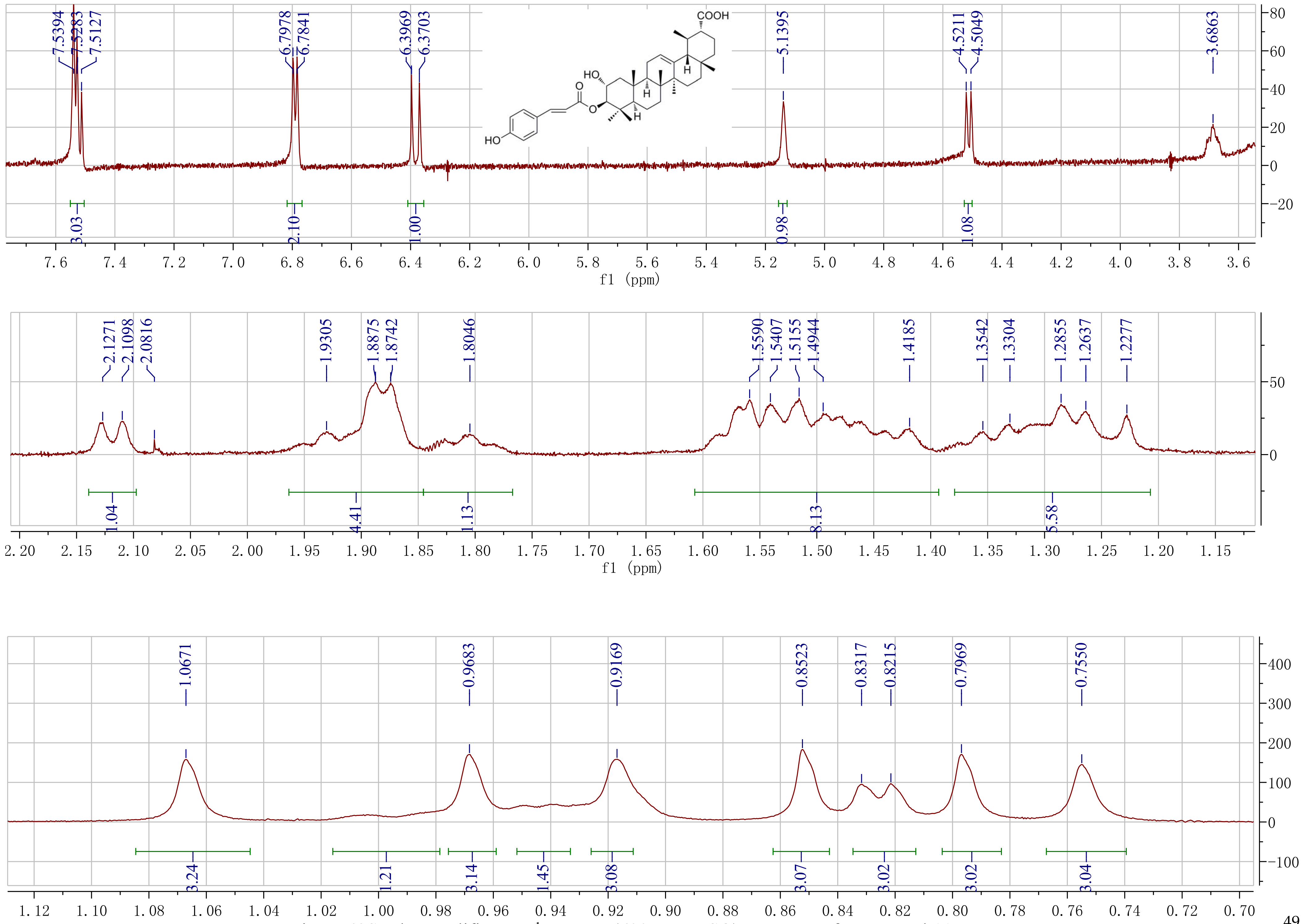

Figure 43S. The amplificatory ^{1}H-NMR (600M, DMSO) spectrum of compound **11**

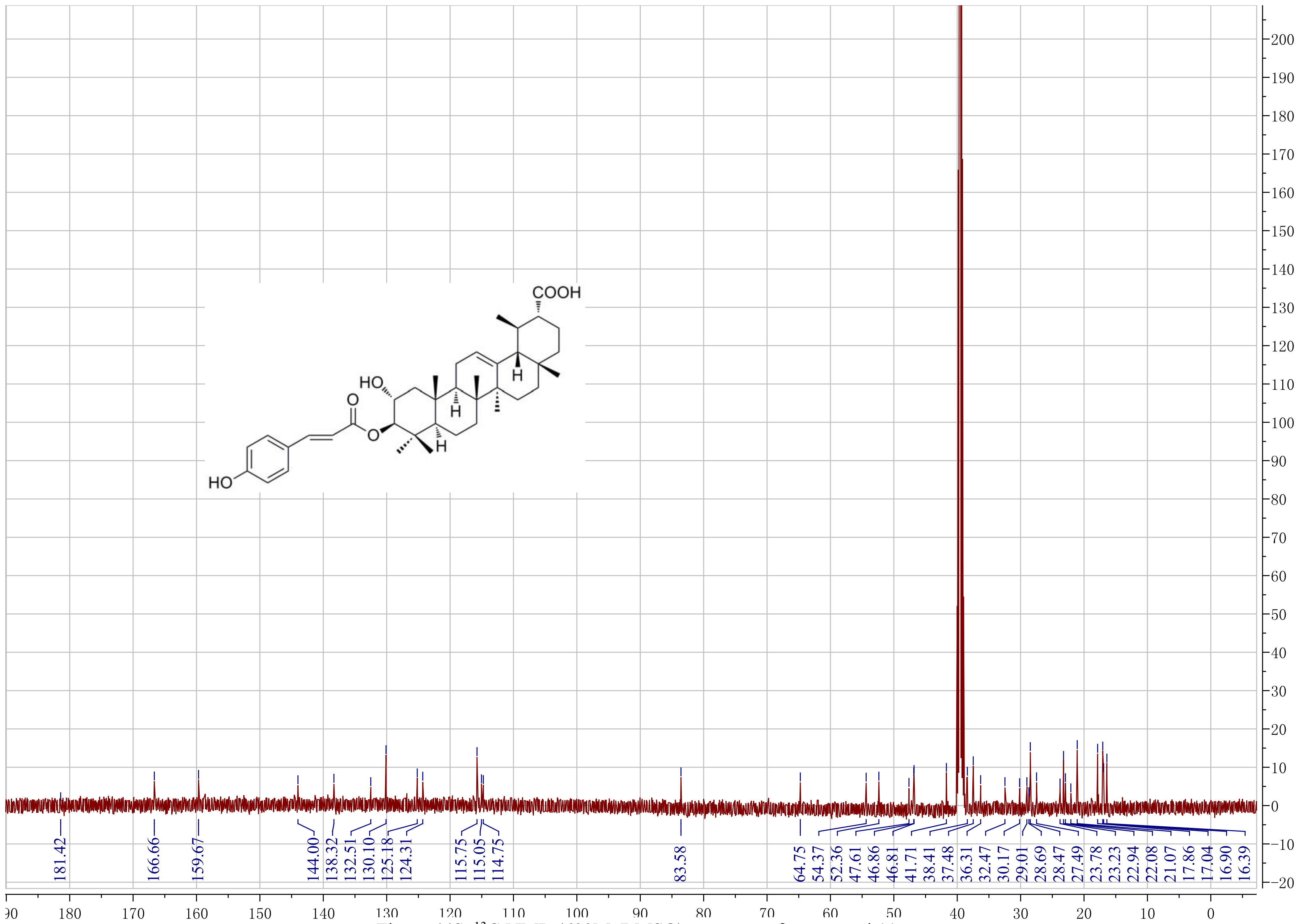

Figure 44S. ^{13}C-NMR (600M, DMSO) spectrum of compound **11**

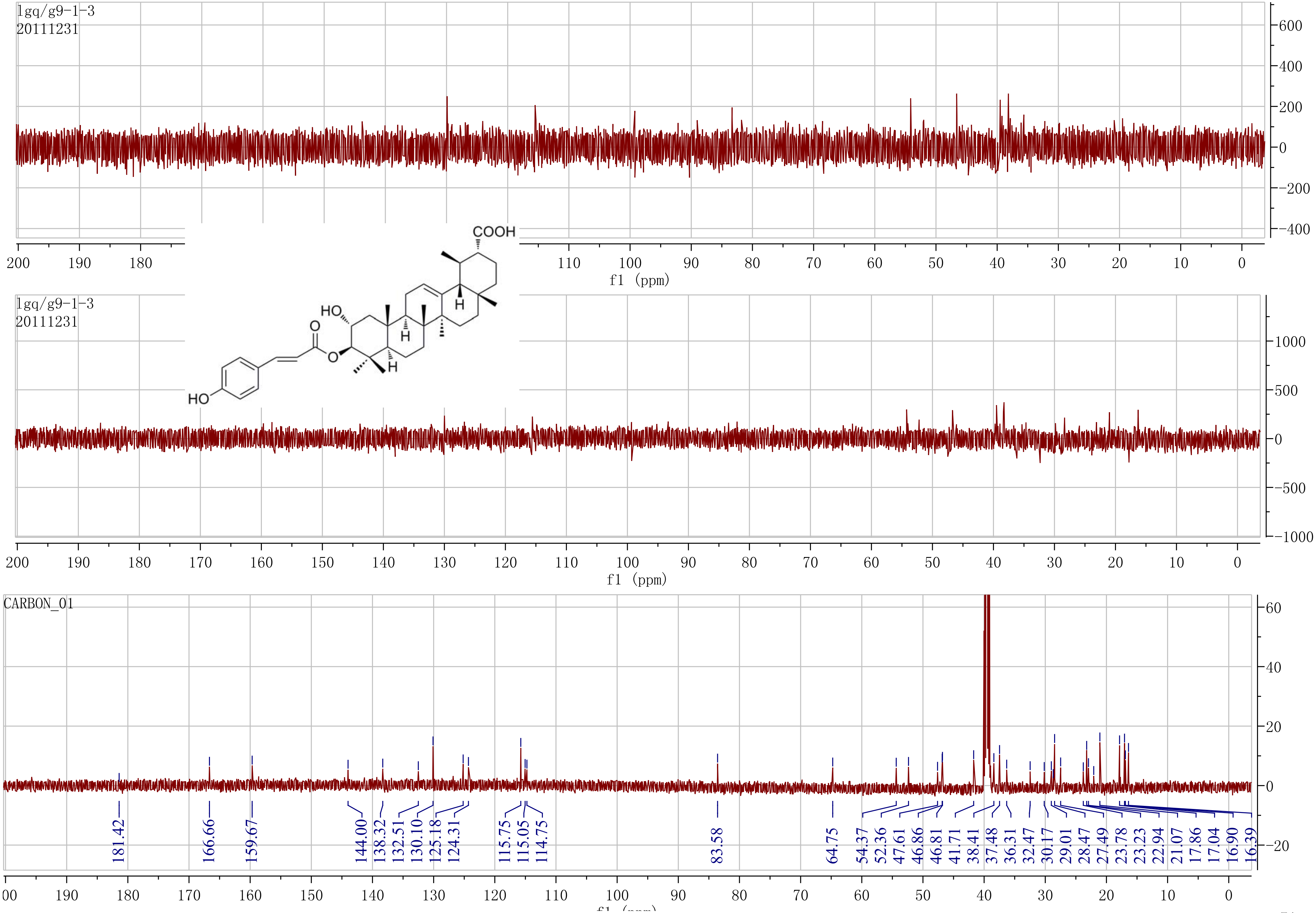

Figure 45S. DEPT (600M, DMSO) spectrum of compound **11**

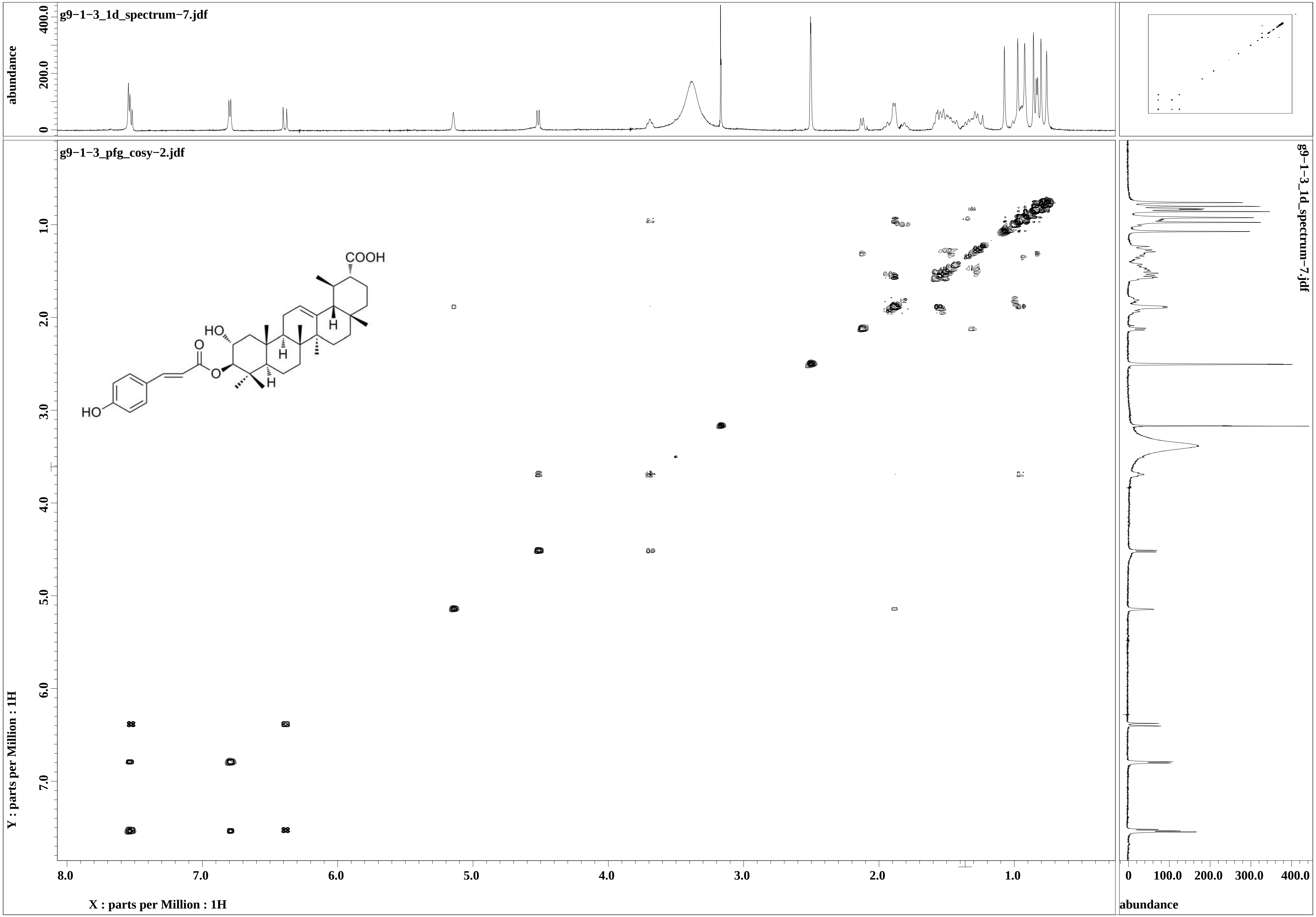

Figure 46S. ^{1}H -^{1}H COSY spectrum of compound **11**

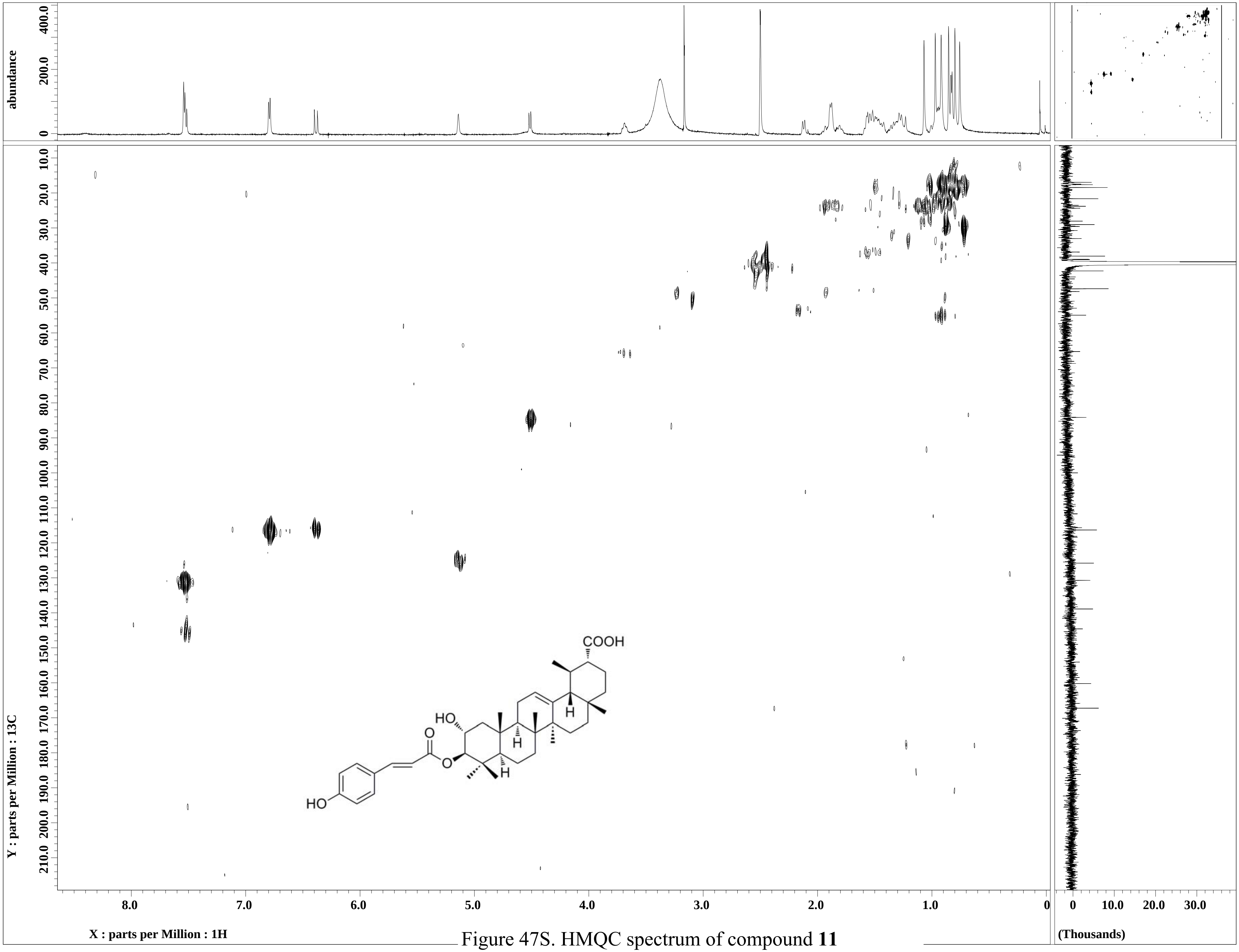

Figure 47S. HMQC spectrum of compound **11**

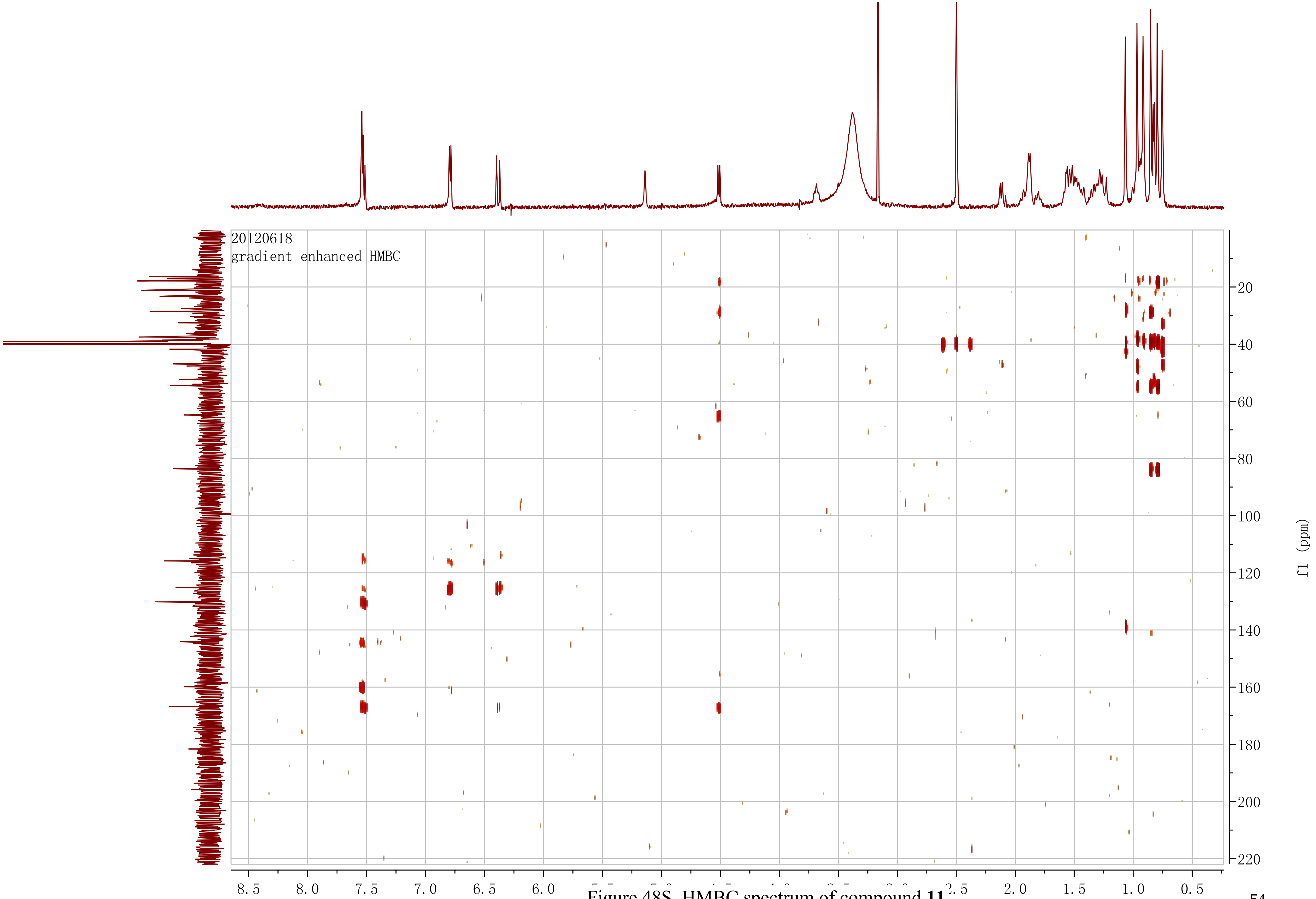

Figure 48S. HMBC spectrum of compound **11**

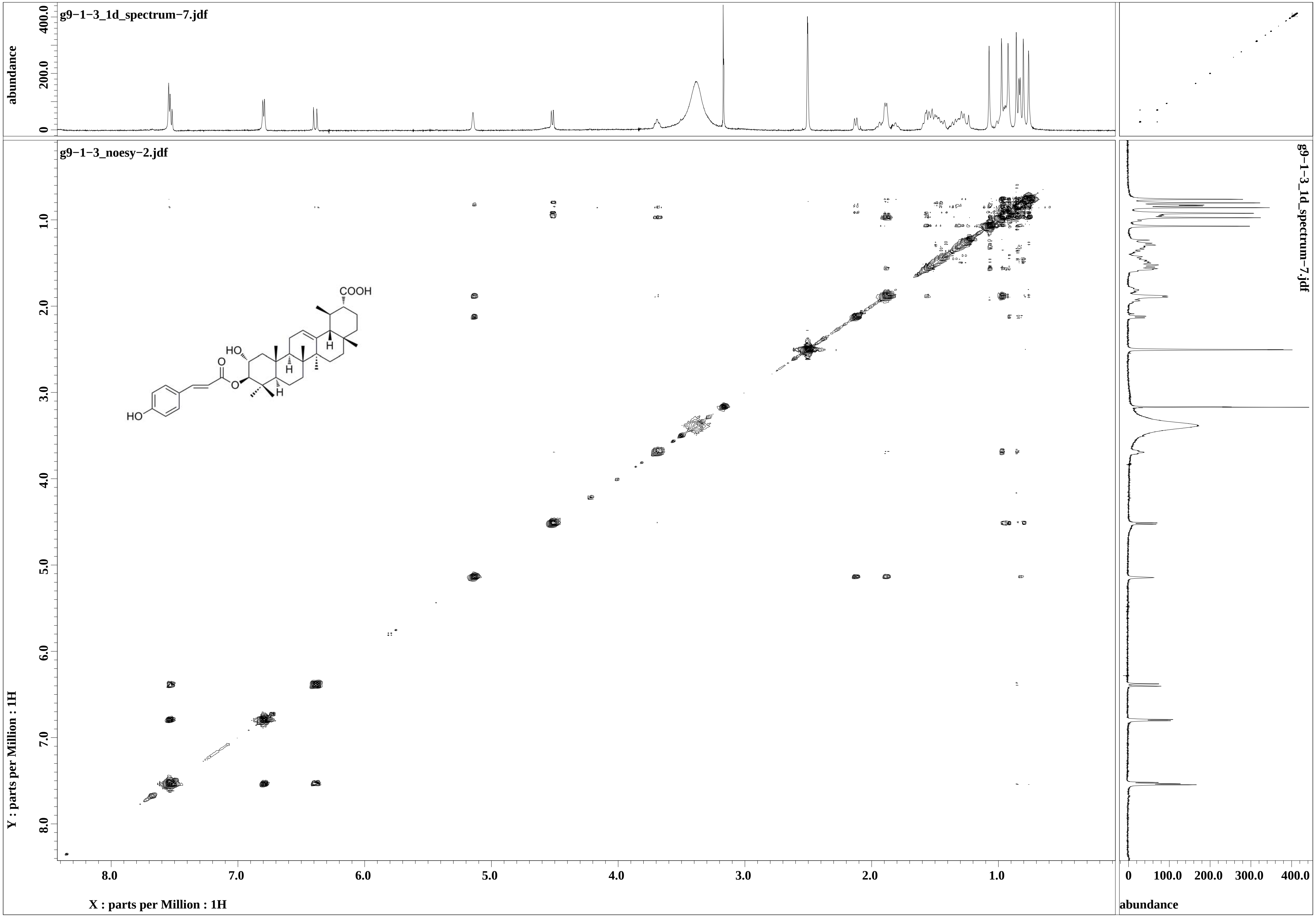

Figure 49S. NOESY spectrum of compound **11**
